# Dietary interventions in skin ageing: a systematic review and meta-analysis

**DOI:** 10.1186/s40101-025-00408-4

**Published:** 2025-10-31

**Authors:** Jun Yan Ng, Xuan Min, Gail Yan Ng, Qi Yi Ambrose Wong, Fook Tim Chew

**Affiliations:** 1 Department of Biological Sciences, Faculty of Science, National University of Singapore, 21 Lower Kent Ridge Rd, Singapore, 117543 Singapore; 2 Allergy and Molecular Immunology Laboratory, Lee Hiok Kwee Functional Genomics Laboratories, Block S2, Level 5, 14 Science Drive 4, Lower Kent Ridge Road, Singapore, 117543 Singapore

**Keywords:** skin aging, skin ageing, nutrition, diet, intervention, systematic review, meta-analysis, wrinkling, hydration, elasticity

## Abstract

**Background:**

Nutrition is a modifiable factor in skin ageing, but its effects remain inconsistently quantified. This meta-analysis assessed human studies from the Web of Science on dietary intake and skin ageing, using pooled standardised mean differences (pSMD). Interventions included carotenoids, collagen, lipids and fatty acids, polyphenols, prebiotics and probiotics, and vitamins. We included full-text English articles and excluded non-human, disease-focused, topical or in vitro studies. Publication bias was assessed using Egger’s test and funnel plots. Results are shown as forest plots.

**Main body:**

Sixty-one studies were meta-analysed. Collagen reduces wrinkles (pSMD = − 0.94 [− 1.39, − 0.49], *p* = 4.82 × 10^−5^). Lipids and fatty acids (pSMD = − 0.62 [− 0.92, − 0.31], *p* = 7.89 × 10^−5^) and polyphenols (pSMD = − 0.48 [− 0.74, − 0.21], *p* = 3.96 × 10^−4^) also reduce wrinkles without significant publication bias. Several interventions improve skin hydration, including collagen (pSMD = 0.66 [0.29, 1.04], *p* = 5.99 × 10^−4^), lipids and fatty acids (pSMD = 0.54 [0.28, 0.80], *p* = 4.36 × 10^−5^), polyphenols (pSMD = 0.59 [0.37, 0.80], *p* = 6.43 × 10^−8^), and prebiotics and probiotics (pSMD = 0.71 [0.25, 1.16], *p* = 2.64 × 10^−3^). Specific interventions target distinct ageing phenotypes. Carotenoids most effectively reduce redness (pSMD = − 0.53 [− 1.02, − 0.04], *p* = 3.39 × 10^−2^), and collagen reduces pigment spots (pSMD = − 0.16 [− 0.31, − 0.003], *p* = 4.56 × 10^−2^). Lipids and fatty acids improve elasticity (pSMD = 0.49 [0.14, 0.83], *p* = 5.45 × 10^−3^), while polyphenols strengthen barrier integrity (trans-epidermal water loss pSMD = − 0.50 [− 0.79, − 0.22], *p* = 6.39 × 10^−4^).

**Conclusion:**

Dietary components target specific skin ageing phenotypes. Carotenoids, collagen, lipids and fatty acids, and polyphenols are particularly effective for redness, pigment spots, elasticity, and barrier integrity, respectively. Lipids, fatty acids, and polyphenols show broad benefits across multiple phenotypes. Shared mechanisms may contribute to overlapping effects. Evidence gaps remain, especially regarding carotenoids and vitamins. Future studies could explore combinatorial dietary interventions. This research is primarily supported by a Singapore National Medical Research Council grant.

**Supplementary Information:**

The online version contains supplementary material available at 10.1186/s40101-025-00408-4.

## Introduction

### Background

The pursuit of healthy ageing has attracted much interest in recent years following a rise in average life expectancy and a growing emphasis on maintaining quality of life into older age. Consistent with this emphasis, the World Health Organization’s 2015–2030 framework for healthy ageing calls for the preservation of functional ability through preventive care, physical activity, and proper nutrition [1]. Among these, nutrition plays a key role not only in supporting general health but also in influencing how visibly and rapidly the skin ages.

The skin, as the body’s largest and most externally visible organ, has received growing attention due to its vulnerability to both intrinsic ageing processes and extrinsic environmental stressors [2]. This dual susceptibility makes the skin a visible indicator of biological ageing, which can be quantified by oneself or by trained investigators using photo-numeric scales and written descriptive scales [3–6].

Emerging evidence suggests that nutritional factors can modulate key skin ageing processes, influencing wrinkles, skin hydration, barrier integrity, and various other skin ageing parameters. As a result, nutrition is increasingly recognised as a key modifiable determinant of skin ageing.

In our previous work, we conducted three systematic reviews and meta‑analyses to quantify the key intrinsic and extrinsic risk factors of skin ageing [7–9]. They are age, sex, ethnicity, genetics, nutrition, smoking, air pollution, and Sun exposure. Effect sizes were expressed as Odds Ratios, reflecting how these outcomes were commonly reported in the literature.

Although our previous analyses identified that lifestyle differences like smoking and Sun exposure significantly increased the odds for skin ageing, we did not assess nutritional factors using the same method due to a methodological difference. Unlike other risk factors, most dietary studies report outcomes as percentage improvements or changes in physical measurements such as wrinkle depth or melanin intensity, rather than as Odds Ratios.

Given this difference in reporting format and recognising the growing relevance of nutrition-focused studies on skin health, we identified a need for a separate, targeted meta-analysis that applies a consistent and appropriate effect metric. Therefore, in the present review and meta-analysis, we focus exclusively on dietary interventions and their effects on skin ageing, using standardised mean differences (SMDs) to quantify intervention outcomes. This unified approach allows us to integrate findings across a wide range of dietary strategies. By doing so, we aim to clarify the relationship between diet and skin ageing, identify consistent patterns across interventions, and provide researchers, clinicians, and public health policymakers with an up-to-date, evidence-based resource to inform dietary recommendations for maintaining skin health throughout the ageing process.

### Definition of skin ageing

In alignment with our previous work [7], we define skin ageing as changes which occur to the skin with time. These changes result in the manifestation of skin ageing phenotypes on the skin. To date, fifty-six distinct skin ageing phenotypes have been identified [8], including forty-one commonly observed traits that can be grouped into ten key principal components [10].

### Aim of the review

This systematic review and meta-analysis aim to consolidate and synthesise findings from studies that investigate the effects of dietary interventions on skin ageing. By quantifying a wide range of outcomes across diverse nutritional strategies and identifying recurring patterns, we seek to clarify the relationship between diet and skin ageing. In addition to overall skin ageing outcomes, many of these studies also examined related skin parameters such as sebum production and skin thickness. Where applicable, these parameters were also included in our analysis and reported accordingly.

## Methodology

### Search strategy

This review was conducted according to the Preferred Reporting Item for Systematic Review and Meta-Analyses (PRISMA) guidelines (Additional Files 1 and 2). A standard protocol was used to perform the literature screening, similar to our previous literature reviews and meta-analyses [7–9].

**Fig. 1:**
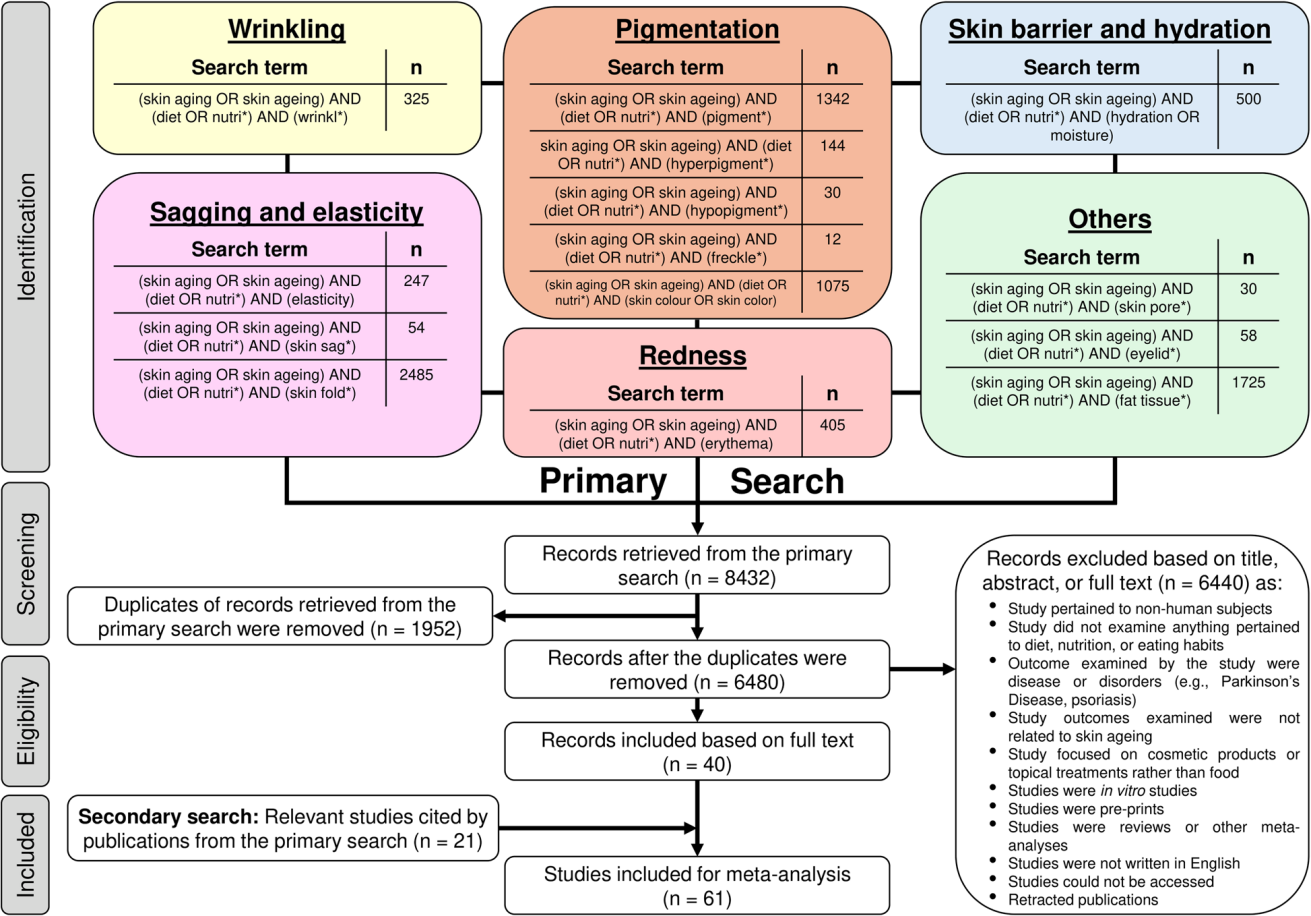
PRISMA flow diagram of study selection. The primary literature search was conducted on the Web of Science database. Filters applied included language restriction to English and exclusion of pre-print articles. Search terms were last updated and assessed on 3 June 2025

A comprehensive primary literature search was performed using the Web of Science database retrieving publications up to the end of 2024. The search strategy employed a broad set of terms related to skin ageing phenotypes such as wrinkles, sagging, skin elasticity, pigmentary changes, redness, skin barrier function, and hydration combined with keywords related to diet or nutrition, and skin ageing (Fig. 1).

Following removal of duplicate records, titles and abstracts were screened to identify articles meeting predefined inclusion criteria, which were then subjected to full-text review. Studies that fulfilled these criteria were included from the primary search.

Subsequently, a secondary, recursive search was conducted by screening reference lists of the eligible primary articles. This process continued until no further relevant studies were identified. All articles identified through this method were assessed against the same inclusion criteria.

Together, the primary and secondary search processes yielded a final set of eligible journal articles that formed the basis of this meta-analysis. Full details of the included studies are provided in Additional File 3.

### Eligibility criteria

Studies included in this review focused on the effects of dietary nutrient intake as a modifiable extrinsic factor influencing skin ageing outcomes in human subjects, assessed through non-invasive methods. Literature was manually screened by two reviewers (J.Y.N. and X.M.G.Y.N.) independently, followed by open discussion to resolve discrepancies. Only full-text journal articles published in English were considered. Exclusion criteria encompassed studies involving non-human subjects, investigations unrelated to diet, nutrition or eating habits, and studies examining disease states or disorders rather than skin ageing phenotypes. Research focusing on cosmetic or topical treatments rather than dietary interventions, in vitro studies, pre-prints, reviews, meta-analyses, retracted publications, and articles inaccessible for full review were also excluded. Full-text articles excluded from the final screening were predominantly other review papers or meta-analyses.

### Data extraction

The following study characteristics were extracted from the full-text articles included in the review: author(s), year of publication, article title, study design, study method, study period, and the country in which the study was conducted. Information on participants included racial background, sex, age range, mean age ± standard deviation where available, and final sample size, along with sex-specific subgroup counts. Details of the dietary interventions were recorded, including the type of intervention (e.g., collagen, polyphenols), a brief caption, and a detailed description specifying dosage, frequency, and active ingredients. For each outcome, skin ageing phenotypes or parameters were recorded according to the terminology used in the original article as well as unified labels used in this review. Definitions of each phenotype, measurement sites (e.g., photo-exposed vs. photo-protected areas), and the assessment methods (e.g., Corneometer, Sebumeter) were also captured, along with whether measurements were conducted by trained investigators or via instrumentation.

Information on baseline and post-intervention means and standard deviations was also extracted where available. These data form the basis for calculating the standardised mean differences (SMD), as described in the next section on ‘ Statistical analysis’. Full extracted details are presented in Additional File 4.

### Statistical analysis

Meta-analyses were conducted on R with the RStudio interface (R Foundation for Statistical Computing, 2010), using the metafor package (Viechtbauer, 2010). Standardised mean differences (Cohen’s $d$) were calculated to quantify the magnitude of change in skin ageing phenotypes or parameters in response to dietary interventions.

For paired study designs, SMDs were computed using the mean and standard deviation values of each skin phenotype before and after intervention, along with the final sample size. The pooled standard deviation was derived as the square root of the average of the variances across timepoints (Eq. 1). The 95% confidence intervals (95% CI) were constructed using a normal approximation in the form $d \pm 1.96 \times SE$, where the standard error (SE) was approximated as $\sqrt{\frac{1}{n}+\frac{{d}^{2}}{2n}}$ assuming a normal distribution of the effect size where $n$ is the sample size. *P* values were calculated based on *t*-statistics and degrees of freedom determined by the paired sample size. These computations were performed in R. The extracted metrics, SMD, 95% CI, and *p*-values, served as inputs for subsequent forest plot visualisations.

$$d = \frac{\overline x_2- \overline x_1}{\sqrt{\frac{s_1\ ^2\ +\ s_2\ ^2}{2}}}$$

Equation 1: Standardised mean difference (SMD) based on Cohen’s $d$ for paired samples where $d$ is SMD, $\overline x_1$ and ${s}_{1}$ are the sample mean and standard deviations before dietary intervention, and ${\overline{x} }_{2}$ and ${s}_{2}$ are the sample mean and standard deviations after dietary intervention respectively.

Random-effects meta-analyses were then conducted using the DerSimonian and Laird estimator. Studies were categorised according to dietary intervention type, and pooled estimates were computed only for categories that included at least two studies.

The forest plots, funnel plots, and illustrative figures were drawn using the metafor package in RStudio [11] and BioRender respectively.

Forest plots were constructed to visualise study-level effect sizes and pooled estimates. Each plot displayed the individual SMDs, confidence intervals, and relative weights for studies within a dietary category. For dietary groups with multiple studies, pooled SMDs (pSMDs) were depicted as diamonds. Heterogeneity was evaluated using Cochran’s *Q*, the degrees of freedom (df), the corresponding *p* value and the inconsistency index (I^2^). An I^2^ value of 50% or greater, together with a *p* value < 0.05, was considered indicative of substantial heterogeneity. I^2^ values reflect the percentage of variability in effect estimates attributable to between-study heterogeneity rather than chance, with higher values suggesting greater heterogeneity among studies.

Funnel plots were generated to visually assess the presence of small-study effects and potential publication bias (Additional Files 5 and 6). These plots were constructed only for dietary categories with at least three studies to ensure sufficient power. Egger’s test was used to evaluate asymmetry in the funnel plots and the associated *p* values were reported. A *p* value > 0.05 was interpreted as insufficient statistical evidence for publication bias. For dietary groups with only two studies, Begg’s funnel plots were produced without Egger’s test, while groups with fewer than two studies were excluded from this analysis. Asymmetrical funnel plots may indicate small-study effects or bias, whereas symmetrical plots suggest more robust and unbiased results.

For unpaired study designs comparing intervention and control groups, the SMDs were calculated using the difference in group means, standardised by the root mean square of the group variances. Cohen’s $d$ was calculated using the mean difference between intervention and control groups, standardised by the root mean square of the group variances (Eq. 2).

$$d = \frac{{\overline{x} }_{2}- {\overline{x} }_{1}}{\sqrt{\frac{{{s}_{1}}^{2} + {{s}_{2}}^{2}}{2}}}$$

Equation 2: Standardised mean difference (SMD) based on Cohen’s d for unpaired samples without assuming equal variance where $d$ is SMD, $\overline x_1$ and ${s}_{1}$ are the sample mean and standard deviations of the non-interventional group and ${\overline{x} }_{2}$, and ${s}_{2}$ are the sample mean and standard deviations of the interventional group.

The SE was approximated as $\sqrt{\frac{\frac{{{s}_{1}}^{2}}{{n}_{1}}+\frac{{{s}_{2}}^{2}}{{n}_{2}}}{\frac{{{s}_{1}}^{2}+{{s}_{2}}^{2}}{2}}+\frac{{d}^{2}}{2({n}_{1}+{n}_{2})}}$ assuming a normal distribution of the effect size where $d$ is the SMD, ${n}_{1}$ and ${s}_{1}$ are the sample size, and standard deviations of the non-interventional controls while ${n}_{2}$ and ${s}_{2}$ are the sample size and standard deviations of the dietary intervention group. Standard errors and 95% confidence intervals for the standardised mean difference were computed, and statistical significance was assessed using Welch’s *t* test, applying the Welch–Satterthwaite approximation for degrees of freedom to account for unequal group variances. These analyses were also conducted in R using the same procedures described above to calculate SMDs, confidence intervals, and *p* values, which were subsequently used in the meta-analyses and forest plots.

## Results

### Literature search

This meta-analysis aimed to evaluate the role of diet as a risk factor for skin ageing. A total of 61 eligible journal articles were included, comprising 40 articles identified through the primary literature search and screening process, and an additional 21 articles obtained through a recursive secondary search of reference lists.

The primary search was conducted using the Web of Science database using a comprehensive set of search terms targeting skin ageing phenotypes such as wrinkles, sagging, skin elasticity, age-related pigmentary changes, redness, skin barrier integrity, and hydration (Fig. 1). Search results must include mentions of diet or nutrition and the term skin ageing and may additionally include a specific, named skin ageing phenotype.

This initial search yielded 8432 records. After removing duplicate entries, 6480 unique journal articles remained. Titles and abstracts were screened, and articles meeting the inclusion criteria were selected for full-text review. The full inclusion criteria can be found in Fig. 1 and was described earlier in the section on *Eligibility criteria*. Briefly, included articles must be primary research studies written in English that investigate the effect of ingested food items on skin ageing in disease-free humans. Based on these criteria, 40 journal articles were included from the primary search.

A secondary, recursive search was then conducted by screening the reference lists of these 40 eligible articles. References from primary articles were first screened, followed by references of those references (i.e., secondary, tertiary sources, and beyond), continuing iteratively until no new articles were identified. All articles obtained through this process were subjected to the same inclusion criteria. This secondary search returned an additional 21 relevant articles.

In total, 61 journal articles met the eligibility criteria and were included in this meta-analysis.

### Overview of study characteristics

Characteristics of the eligible articles on skin ageing and dietary interventions included in the meta-analyses of this review are summarised in Table 1.

**Table 1:** Demographic information of the sixty-one publications on the effect of dietary interventions on skin ageing parameters used in the meta-analysis

Characteristic	Publications included in the meta-analysis, *n* (%)
**Study design**	
Randomized, controlled trials	47 (77.0%)
Uncontrolled trials	8 (13.1%)
Open-label, no placebo-controlled trial	6 (9.9%)
**Sex**	
Females only	38 (62.3%)
Males only	3 (4.9%)
Both females and males	20 (32.8%)
**Ethnicity**	
Asians	30 (49.2%)
Whites	22 (36.1%)
Multiple ethnicities	2 (3.3%)
Not reported	7 (11.5%)
**Skin ageing phenotypes studied**	
Wrinkling	30 (49.2%)
Hydration	41 (67.2%)
Redness	18 (29.5%)
Pigment spots	17 (27.9%)
Sagging/elasticity	13 (21.3%)
Skin barrier	24 (39.3%)
Sebum	8 (13.1%)
Thickness	8 (13.1%)
Others ^1^	37 (60.7%)
**Dietary interventions**	
Carotenoids	8 (13.1%)
Collagen	17 (27.9%)
Lipids and fatty acids	11 (18.0%)
Polyphenols	15 (24.6%)
Prebiotics and probiotics	7 (11.5%)
Vitamins	3 (4.9%)

^1 ^Other phenotypes refer to blemishes, brightness, clearness, dark eye circles, density, distensibility (R0), edema, fatigue resistance (R9), gloss, immediate elastic skin recovery ratio (R7), laxity, net elasticity (R5), pores, radiance, skin biological age, skin carotenoid concentrations, skin pH, skin scaling, skin tone, swelling, and visco-elasticity (R2). R0, R2, R5, R7, and R9 are R-parameters in Cutometer measurements, representing various aspects of skin elasticity

Among the 61 studies included in this meta-analysis, the majority were randomised controlled trials (*n* = 47, 77.0%). A minority of studies were either uncontrolled trials (*n* = 8, 13.1%) or open-label studies without placebo controls (*n* = 6, 9.9%). Given the methodological heterogeneity across studies, including differences in trial design, population characteristics, and outcome assessment, a random effects model was employed to account for between-study variance and to generate pooled estimates that reflect both within- and between-study variability. This allows for more generalisable estimates of the dietary effects on skin ageing.

Most studies focused on female participants (*n* = 38, 62.3%), while only three studies (4.9%) examined males exclusively [12–14]. The remaining studies included both females and males (*n* = 20, 32.8%). Most studies were conducted on Asian (*n* = 30, 49.2%) or White (*n* = 22, 36.1%) participants. A small number of studies included participants from multiple racial groups (*n* = 2, 3.3%). In 7 studies (11.5%), the racial composition of the study group was not reported. The youngest participants were 18 years old [15] and the oldest participants were 84 years old [16].

Across the included studies, a diverse range of skin ageing phenotypes was investigated. Wrinkling (*n* = 30, 49.2%) and skin hydration (*n* = 41, 67.2%) were the most frequently assessed outcomes, followed by skin redness (*n* = 18, 29.5%) and age-related pigmentary changes (*n* = 17, 27.9%). Measures of skin sagging and elasticity (*n* = 13, 21.3%), skin barrier function (*n* = 24, 39.3%), and skin thickness (*n* = 8, 13.1%) were also commonly reported. In addition, a considerable number of studies (*n* = 37, 60.7%) reported other skin ageing parameters such as blemishes, brightness, clearness, gloss and biological skin age within the same publication. However, each of these additional phenotypes was examined in no more than three individual studies, limiting the ability to draw meaningful conclusions regarding their response to dietary interventions.

As many publications examined multiple skin ageing parameters within a single study, the total number of phenotype reported exceeds the number of included articles (*n* = 61). Further details and a more in-depth discussion of the phenotypes and parameters investigated can be found in the following section titled *Overview of Skin Ageing Phenotypes*.

The dietary interventions examined were wide-ranging. Collagen-based interventions were the most common (*n* = 17, 27.9%), followed by polyphenols (*n* = 15, 24.6%), lipids and fatty acids (*n* = 11, 18.0%), carotenoids (*n* = 8, 13.1%), prebiotics and probiotics (*n* = 7, 11.5%) and vitamins (*n* = 3, 4.9%). The shortest intervention spanned 2 weeks [13] and the longest intervention lasted 6 months [17–19]. A detailed breakdown of the dietary interventions can be found two sections later, in the section titled *Overview of Dietary Interventions*.

Together, these studies represent a diverse set of experimental evidence exploring the effect of dietary interventions on skin ageing phenotypes and parameters.

### Overview of Skin Ageing Phenotypes

**Fig. 2:**
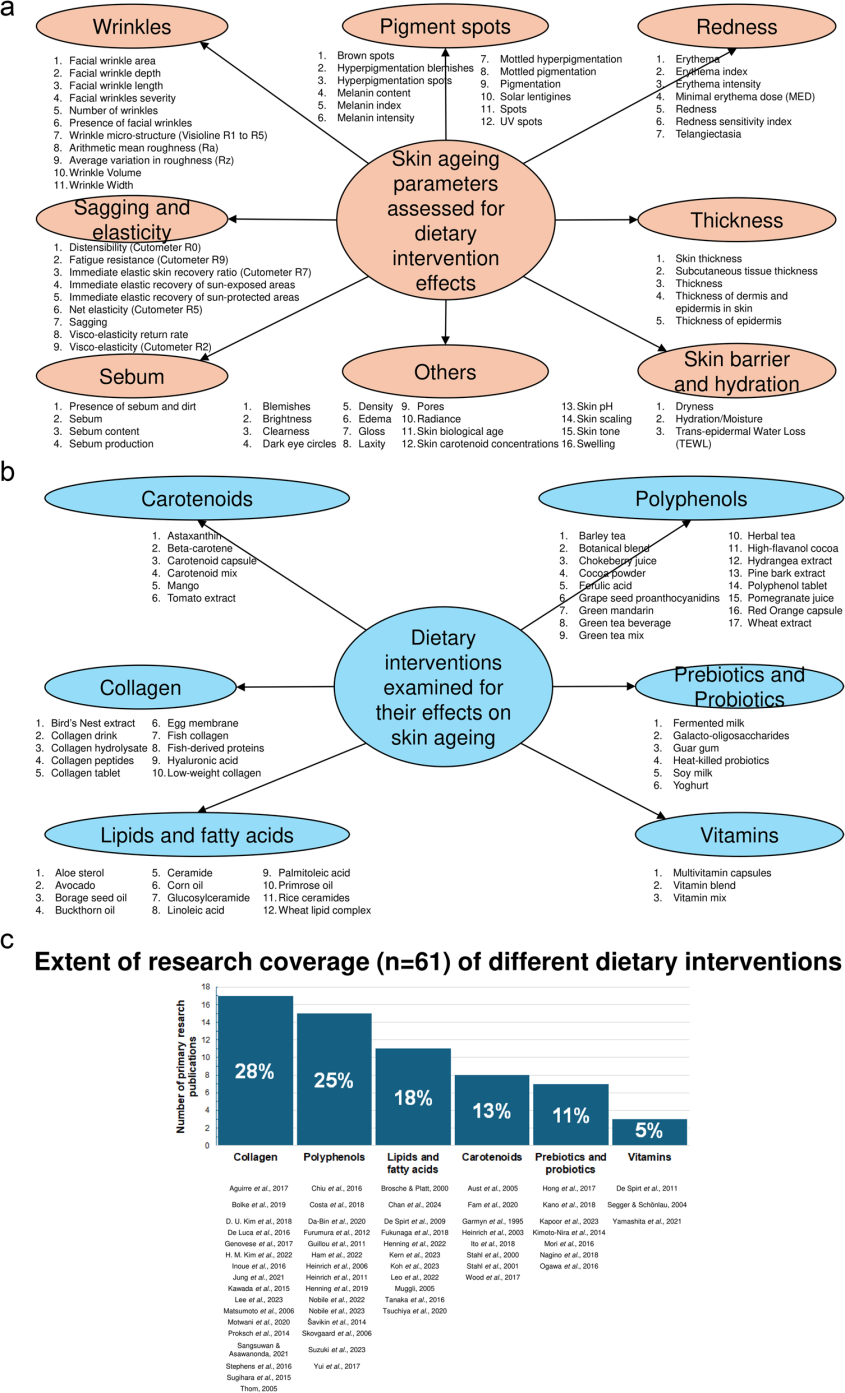
**a** Skin ageing outcome measures reported in the published literature on dietary interventions. All outcome measures have been grouped into the following categories: wrinkling, pigment spots, redness, sagging and elasticity, thickness, sebum, skin barrier, hydration, and others. Specific outcome measures reported in individual studies are listed below each corresponding category. **b** Dietary interventions reported in the published literature for their effects on skin ageing outcomes. Interventions have been categorised into the following groups: carotenoids, polyphenols, collagen, prebiotics and probiotics, lipids and fatty acids, and vitamins. Specific skin ageing outcome measures assessed in individual studies are listed below each corresponding category. **c** Research coverage of dietary interventions studied for their effects on skin ageing phenotypes. Dietary interventions have been categorised into the following groups: carotenoids, polyphenols, collagen, prebiotics and probiotics, lipids and fatty acids, and vitamins. Individual studies examining each intervention are listed below the corresponding category

A range of skin ageing outcome measures has been reported in the literature examining the effects of dietary interventions. These outcomes include skin ageing phenotypes such as wrinkles, pigment spots, redness, sagging, elasticity, skin barrier function, and hydration. In addition to these skin ageing phenotypes, several studies have concurrently examined related skin parameters, including sebum production and skin thickness. These parameters were therefore included in our analysis using the same methodological framework (Fig. 2a).

The outcomes listed above represent the most studied parameters in the context of skin ageing. Other parameters, such as blemishes [15], gloss [20, 21], skin pH [12, 22, 23], and skin tone [14, 24], have also been reported; however, each of these has been investigated in no more than three studies and thus could not be meaningfully pooled for meta-analytic synthesis. A comprehensive overview of the diversity and scope of studies assessing dietary interventions on skin ageing is provided in Additional File 7.

### Overview of Dietary Interventions

Dietary interventions investigated in the literature can be broadly classified into six groupings: carotenoids, collagen, lipids and fatty acids, polyphenols, prebiotics and probiotics, and vitamins (Fig. 2b). The rationale, sources, and supporting references for this classification are provided in Additional File 3.

Collagen is the most extensively studied compound in relation to skin ageing. It has been administered either as tablets [19, 24–26] or as isolated peptides [21, 23, 27–37], and is derived from a range of sources such as Edible Bird’s Nest [31], eggs [27], and fish [23, 34]. Lipids and fatty acids is a heterogeneous group, consisting of essential polyunsaturated fatty acids [16, 38–41], monounsaturated fatty acids [39, 42], sphingolipids [22, 43, 44], esterified lipids [38], aloe sterols [45], and glycolipids [46]. Polyphenols are similarly diverse in origin and consist of plant-derived compounds broadly classified into flavonoids [17, 47–53], phenolic compounds [13, 20, 46, 54], and condensed tannins [18, 55].

Of the 61 primary research articles included in our review, collagen-based interventions are the most well-represented, accounting for 17 studies (28%) (Fig. 2c), followed closely by polyphenols, with 15 studies (25%). In contrast, vitamins are the least studied dietary intervention category, with only three (5%) published articles identified [56–58]. A two-by-two matrix summarising the diversity and scope of publications investigating dietary interventions, stratified by both skin ageing parameter and type of dietary intervention, is presented in Additional File 8.

Notably, there appears to be a disproportionate research focus on the effects of carotenoids on skin redness, with eight publications addressing this outcome [59–66]. In contrast, other skin ageing phenotypes, such as elasticity [66], hydration [63], and skin barrier function [66], have received considerably less attention, each being the focus of only one publication. No studies were identified that examined the effects of carotenoids on pigment spots.

### Wrinkling

**Fig. 3:**
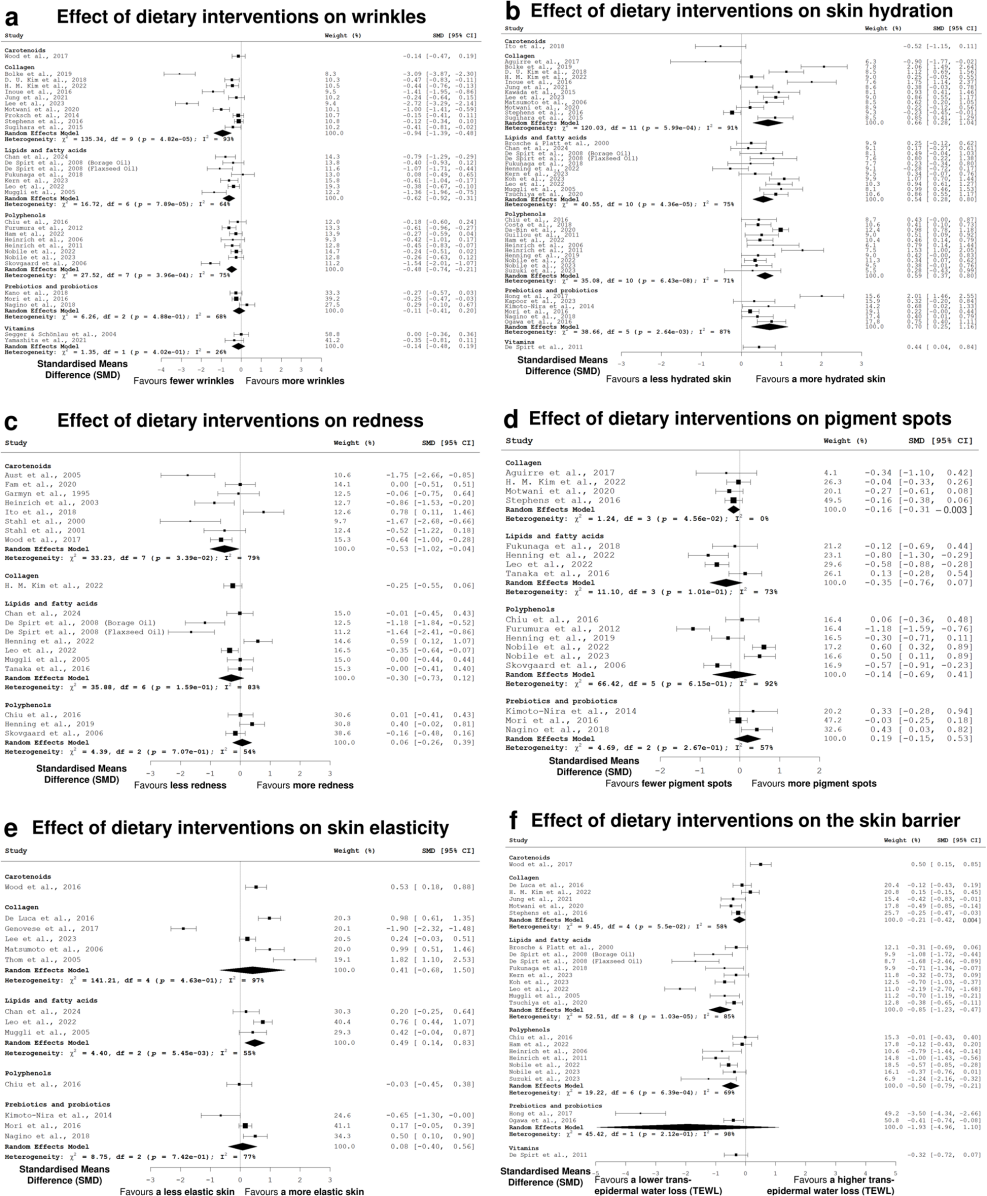
**a**–**f** Forest plot summarising the effect sizes, quantified as standardised means difference (SMD), for studies assessing the impact of dietary interventions on **a** wrinkles, **b** hydration, **c** redness, **d** pigment spots, **e** elasticity, and **f** the skin barrier compared to before the dietary intervention. Each circle represents a study's effect size. The size of each circle is proportional to its weight in the meta-analysis. Horizontal lines denote 95% confidence intervals. SMD for each study was calculated using Cohen’s d for paired samples (i.e., before vs. after dietary intervention). The vertical dotted line indicates the line of no effect (SMD = 0). A negative SMD indicates that the dietary intervention favours less of the studied phenotype (e.g., less wrinkles) when compared to before the dietary intervention. A positive SMD indicates that the dietary intervention favours more of the studied phenotype (e.g., more skin hydration) when compared to before the dietary intervention. The pooled effect estimate and pooled 95% confidence interval (CI) are computed based on a random effects model and shown as a diamond, in which the diamond’s width represents the range of the 95% CI. The I^2^ statistic quantifies the proportion of total variation in results across studies investigating the same dietary intervention that is due to heterogeneity rather than chance. An I^2^ value of 0% indicates no observed heterogeneity; the group of studies examining this dietary intervention are relatively homogeneous. Larger I^2^ values indicate greater heterogeneity. *SMD* standardised means difference, *CI* confidence interval, χ^2^ chi-square, *df* degrees of freedom, *p* chi-square test *p* value, *I*^2^ heterogeneity statistic

Dietary interventions were associated with fewer wrinkles, with the most consistent and statistically robust effects observed for lipids and fatty acids, as well as polyphenols (Fig. 3a).

Collagen, lipids and fatty acids, and polyphenols each produced significant pooled standardised mean differences (pSMDs), indicating reductions in wrinkle appearance. Collagen supplementation yielded a significant reduction in wrinkles (pSMD [95% CI] = − 0.94 [− 1.39, − 0.49], *p* value = 4.82 × 10^−5^); however, strong evidence of publication bias (Egger’s test *p* value = 2.92 × 10^−12^) and the presence of substantial heterogeneity (I^2^ = 93.4%) limit confidence and generalisability in this finding. An I^2^ value of 93.4% indicates that the studies reported in the literature were very heterogenous, suggesting that variations in study design, participant groups, collagen type, or dosage and outcome measurements may strongly influence the observed effects. A low Egger’s test *p* value suggests that there may be publication bias in that smaller studies with positive results are more likely to have been published than those showing no effect. Taken together, these measures indicate that while collagen supplementation may confer some benefit for wrinkle reduction, further large-scale, high-quality trials are required to establish its true efficacy.

In contrast to collagen, the effect of lipids and fatty acids (pSMD [95% CI] = − 0.62 [− 0.92, − 0.31], *p* value = 7.89 × 10^−5^; Egger’s test *p* value = 0.2881; I^2^ = 64.1%) and polyphenols (pSMD [95% CI] = − 0.48 [− 0.74, − 0.21], *p* value = 3.96 × 10^−4^; Egger’s test *p* value = 0.2469; I^2^ = 74.6%) remained statistically significant and were not associated with publication bias. Although some heterogeneity was present, the direction and consistency of the effects, together with their statistical significance, suggest that these findings are reasonably generalisable and may reflect meaningful benefits across a range of populations and study designs.

The effects of prebiotics and probiotics (pSMD [95% CI] = − 0.11 [− 0.41, 0.20], *p* value = 0.488) and vitamins (pSMD [95% CI] = − 0.14 [− 0.48, 0.19], *p* value = 0.402) did not reach statistical significance. The carotenoid category could not be evaluated meta-analytically, as only one study was available [66].

### Hydration

Dietary interventions were positively associated with improved skin hydration, with significant and directionally consistent effects observed for collagen, lipids and fatty acids, polyphenols and prebiotics and probiotics. None of these groups showed significant evidence of publication bias (Fig. 3b).

Collagen supplementation improved skin hydration (pSMD [95% CI] = 0.66 [0.29, 1.04], *p* value = 5.99 × 10^−4^; Egger’s test *p* value = 0.5082), with high heterogeneity (I^2^ = 90.8%) suggesting variability in effect size, though the direction of effect was consistent across 12 studies. Lipids and fatty acids also showed a significant benefit (pSMD [95% CI] = 0.54 [0.28, 0.80], *p* value = 4.36 × 10^−5^; Egger’s test *p* value = 0.5197), with moderate heterogeneity (I^2^ = 75.3%) indicating variation in magnitude but a consistent positive direction.

Polyphenols (pSMD [95% CI] = 0.59 [0.37, 0.80], *p* value = 6.43 × 10^−8^; Egger’s test *p* value = 0.8643; I^2^ = 71.5%) and prebiotics and probiotics (pSMD [95% CI] = 0.71 [0.25, 1.16], *p* value = 2.64 × 10^−3^; Egger’s test *p* value = 0.2237; I^2^ = 87.1%) likewise showed significant positive effects, with heterogeneity reflecting differences in study population rather than inconsistency in effect direction. Vitamin-based interventions [56] and carotenoids [63] could not be evaluated using meta-analytic pooling, as only one study was available for each category.

### Carotenoids reduce skin redness

Among dietary interventions, only carotenoids were significantly associated with reduced skin redness. Carotenoid supplementation led to an improvement (pSMD [95% CI] = − 0.53 [− 1.02, − 0.04], *p* value = 0.0339), with no significant evidence of publication bias (Egger’s test *p* value = 0.1493) despite moderate heterogeneity (I^2^ = 78.9%), suggesting a consistent overall effect across studies (Fig. 3c).

Other nutrient groups, including lipids and fatty acids (pSMD [95% CI] = − 0.30 [− 0.73, 0.12], *p* value = 0.159; Egger’s test *p* value = 0.0294), polyphenols (pSMD [95% CI] = 0.06 [− 0.26, 0.39], *p* value = 0.707), and collagen, prebiotics and probiotics, and vitamins (not pooled due to insufficient data), did not show statistically significant effects.

These results highlight carotenoids as the most promising dietary intervention for reducing skin redness based on current evidence.

### Collagen reduces pigment spots

Collagen supplementation was significantly associated with a reduction in pigment spots. A small but statistically significant improvement was observed (pSMD [95% CI] = − 0.16 [− 0.31, − 0.003], *p* value = 0.0456), with no evidence of publication bias (Egger’s test *p* value = 0.6288) and no observed heterogeneity (I^2^ = 0%) (Fig. 3d).

Other dietary groups, including lipids and fatty acids (pSMD [95% CI] = − 0.35 [− 0.76, 0.07], *p* value = 0.101), polyphenols (pSMD [95% CI] = − 0.14 [− 0.69, 0.41], *p* value = 0.615), and prebiotics and probiotics (pSMD [95% CI] = 0.19 [− 0.15, 0.53], *p* value = 0.267), did not produce significant effects. There were no studies on the effect of carotenoids and vitamins on pigment spots.

These findings suggest that collagen is the most promising dietary intervention for reducing pigment spots, with statistically significant evidence across studies.

### Lipids and fatty acids improve skin elasticity

Lipids and fatty acids were associated with a significant improvement in skin elasticity. A moderate and statistically significant effect was observed (pSMD [95% CI] = 0.49 [0.14, 0.83], *p* value = 5.45 × 10^−3^), with no significant evidence of publication bias (Egger’s test *p* value = 0.0538). Heterogeneity was moderate (I^2^ = 54.5%), indicating some variation across studies but with a consistent positive direction of effect (Fig. 3e).

Other dietary groups did not show statistically significant improvements. Collagen supplementation showed a non-significant effect (pSMD [95% CI] = 0.41 [− 0.68, 1.50], *p* value = 0.463) and prebiotics and probiotics (pSMD [95% CI] = 0.08 [− 0.40, 0.56], *p* value = 0.742) also did not produce a significant pooled effect size.

Meta-analytic pooling was not possible for carotenoids [66], polyphenols [17], or vitamins due to insufficient data. These findings highlight lipids and fatty acids as the most promising dietary intervention for improving skin elasticity.

### Polyphenols improve skin barrier integrity

Polyphenol intake was associated with improved skin barrier integrity, measured by reductions in trans-epidermal water loss. A significant effect was observed (pSMD [95% CI] = − 0.50 [− 0.79, − 0.22], *p* value = 6.39 × 10^−4^), with moderate heterogeneity (I^2^ = 68.8%) and no significant evidence of publication bias (Egger’s test *p* value = 0.0939) (Fig. 3f).

Lipids and fatty acids also demonstrated a significant effect (pSMD [95% CI] = − 0.85 [− 1.23, − 0.47], *p* value = 1.03 × 10^−5^). The pooled results showed high heterogeneity (I^2^ = 84.8%) and significant evidence of publication bias (Egger’s test *p* value = 0.0338), which limits confidence in the interpretation and generalisability of this finding.

Collagen showed a borderline non-significant effect on skin barrier integrity (pSMD [95% CI] = − 0.21 [− 0.42, 0.004], *p* value = 0.055) with moderate heterogeneity (I^2^ = 57.7%) and no indication of publication bias (Egger’s test *p* value = 0.4799). Carotenoids [66] and vitamins [56] could not be meta-analysed due to an insufficient number of studies.

### Other skin ageing parameters

Sebum production and skin thickness were analysed using the same methodological approach as for the other skin ageing phenotypes.

Although dietary interventions involving collagen [23, 25] (pSMD = 0.11), lipids and fatty acids [22, 42] (pSMD = 1.04), polyphenols [12, 54] (pSMD = 0.04), and prebiotics and probiotics [15, 67] (pSMD = 0.41) appeared to be associated with increased sebum production, none of these pSMDs reached statistical significance (Additional File 9).

Similarly, interventions with collagen [19, 24, 28] (pSMD = 0.99) and polyphenols [20, 47, 48, 53] (pSMD = 0.29) were suggestive of an increase in skin thickness, but again, the pSMDs did not reach statistical significance (Additional File 10). One individual study reported that vitamin intake [56] was associated with increased skin thickness (SMD = 0.45); however, as this finding is based on a single study, it could not be included in meta-analytic pooling.

Parameters of skin elasticity measured using a Cutometer, such as R0 (distensibility), R2 (visco-elasticity), R5 (net elasticity), R7 (immediate elastic skin recovery ratio), and R9 (fatigue resistance), were assessed using a different methodology. As such, they were analysed separately and are presented in Additional Files 11 to 15.

The data presented thus far are derived from paired-sample analyses comparing participants’ skin measurements following dietary intervention with their baseline values. Most of these studies also included comparisons between subjects receiving dietary interventions and non-interventional control groups. Results from these comparative analyses are similar and are reported in Additional Files 16 to 28.

Notably, polyphenol-based dietary interventions demonstrated efficacy in both improving skin hydration and strengthening the skin barrier. Participants receiving polyphenol supplementation exhibited significant increases in skin hydration (pSMD [95% CI] = 0.54 [0.32, 0.76], *p* = 1.48 × 10^−6^) and significant reductions in trans-epidermal water loss (TEWL) (pSMD [95% CI] = − 0.58 [− 0.91, − 0.26], *p* = 4.54 × 10^−4^) when compared with non-interventional controls. No significant publication bias was detected, as indicated by Egger’s test *p* values of 0.5282 for skin hydration and 0.6444 for skin barrier integrity.

## Discussion

### Dietary effects on skin ageing: From targeted benefits to broad improvements

Diet plays an important role in modulating skin health and the ageing process. There has been a growing interest in studying nutritional interventions aimed at reducing skin ageing, with the goal of identifying effective nutritional strategies to maintain skin integrity and reduce skin ageing. To synthesise the current evidence, we conducted a comprehensive meta-analysis evaluating the effects of different dietary components on key skin ageing parameters.

Our meta-analysis utilised pooled standardised mean differences (SMDs) and assessed the presence of publication bias to ensure robustness. This rigorous method revealed three key insights (Table 2).

**Table 2:**
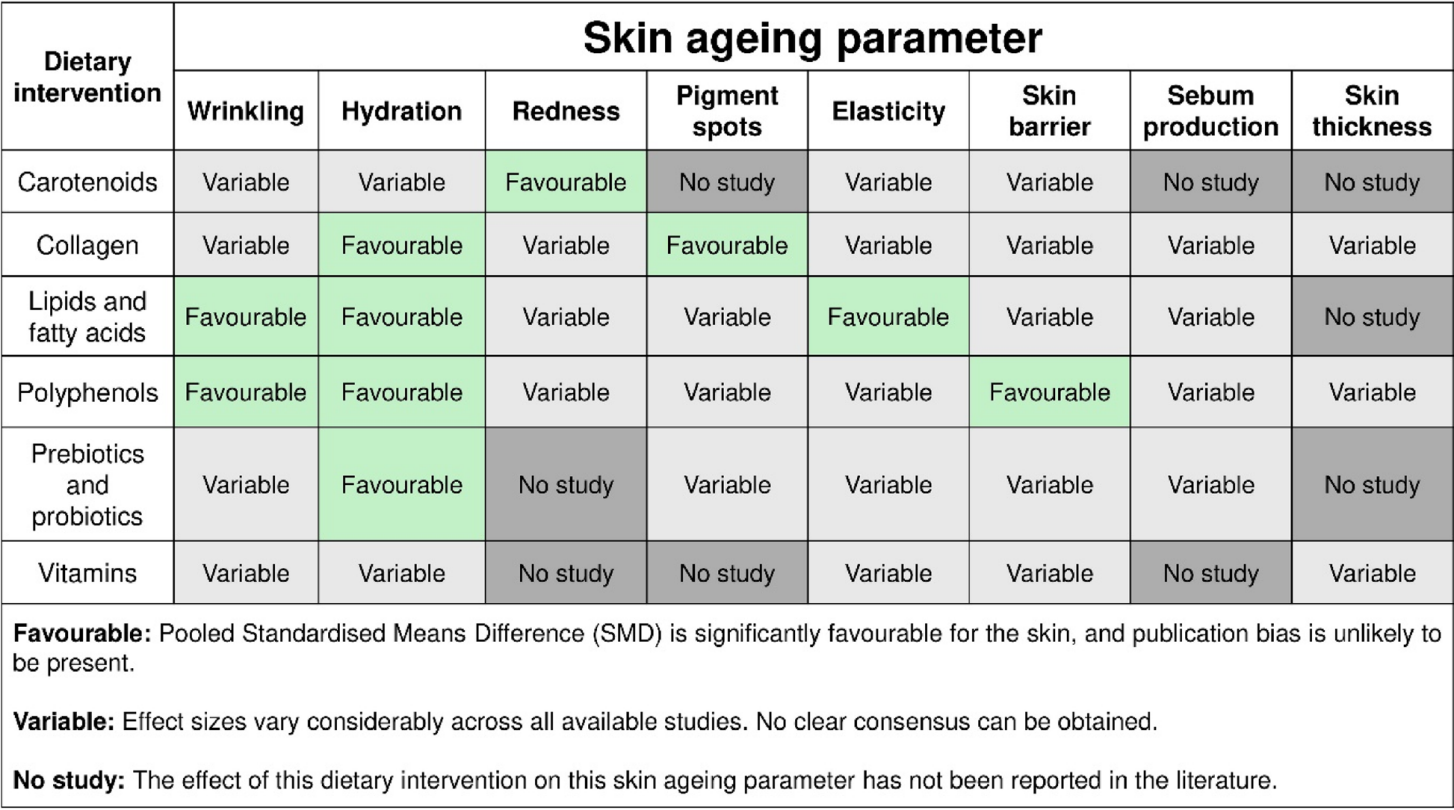
Summary of meta-analytic evidence for dietary interventions on skin ageing parameters. ‘Favourable’ indicates a significant pooled effect size and no significant evidence of publication bias. ‘Variable’ means that effect sizes vary across studies, ranging from beneficial to non-significant. ‘No study’ indicates that no data is available for that dietary intervention-parameter pair

First, certain dietary interventions appear to target specific skin ageing outcomes more effectively than others. For instance, carotenoids demonstrated the strongest effect in reducing skin redness [59–66], whereas collagen supplementation was most effective in decreasing the appearance of pigment spots [24, 27, 31, 34]. Lipids and fatty acids produced the strongest effects on improving skin elasticity [22, 40, 41], while polyphenols were the most effective in strengthening the skin barrier [13, 17, 20, 47–49, 52].

Second, multiple dietary interventions were effective in improving both wrinkles and skin hydration, suggesting the potential involvement of shared or overlapping biological pathways. We found that both dietary interventions involving lipids and fatty acids [22, 38, 40, 41, 44, 46], and interventions involving polyphenols [17, 18, 20, 47–49, 52, 55], demonstrated significant efficacy in reducing wrinkle formation. In a similar idea, four of the six studied dietary interventions: collagen [21, 23, 24, 26–29, 31–34, 37], lipids and fatty acids [16, 22, 38–44, 46], polyphenols [12, 13, 17, 20, 47–50, 52, 68], and prebiotics and probiotics [15, 67, 69–72], were shown to be effective in improving skin hydration. These findings suggest that different dietary components may influence common cellular mechanisms to promote skin health. Future research should investigate whether these effects are additive or synergistic by conducting combinatorial intervention trials. For example, studies examining the combined effects of lipids, fatty acids, and polyphenols may reveal whether such combinations bring about greater improvements in wrinkle reduction than individual interventions alone.

Third, for individuals seeking broad improvements in skin health and healthy skin ageing, rather than targeting specific phenotypes, interventions involving lipids and fatty acids or polyphenols appear to offer the most comprehensive benefits. Strong and consistent evidence supports the intake of essential fatty acids from sources such as avocados [42], borage seed oil [16, 38], corn oil [39], evening primrose oil [41, 73], flaxseeds [38], and sea buckthorn oil [40]. In addition, ceramides from cultivated Asian rice [22], glucosylceramide derived from torula yeast (*Candida utilis*) [44], and fermented milk with *Acetobacter malorum* NCI 1683, a dihydroceramide-rich bacterium [43], have shown similar beneficial effects. Plant sterol lipids such as aloe sterol [14, 45] and glycolipids from wheat [46] further contribute to skin improvements. Collectively, these interventions lead to reduced wrinkle formation, enhanced hydration, and improved elasticity. Similarly, dietary polyphenols, including flavonoids from chokeberries [53], cocoa powder [48], green mandarin extract [49], green tea [17, 47], *Hydrangea serrata* leaf extracts [50], lemon balm tea [51], and red orange complex [52]; phenolic compounds from pomegranate [54] and wheat [46]; condensed tannins from French maritime pine bark extract [55]; and white tea and grape seed extracts [12, 18] have demonstrated broad-ranging efficacy ranging from wrinkle reduction, improved hydration, to a stronger skin barrier integrity.

Overall, these findings highlight the potential of dietary interventions to bring about both targeted and broad-spectrum improvements to skin health and reduce skin ageing.

Important gaps remain. Carotenoids and vitamins are the least well studied dietary interventions. To date, no published studies have examined the effects of carotenoids on pigment spots, sebum production, or skin thickness. Similarly, there is a lack of evidence on the effects of vitamins on skin redness, pigment spots, and sebum production. While further research is needed, these effects offer a promising foundation for advancing our understanding of the biological mechanisms linking diet and skin ageing.

### Carotenoids in photo-protection and reducing skin redness

**Fig. 4:**
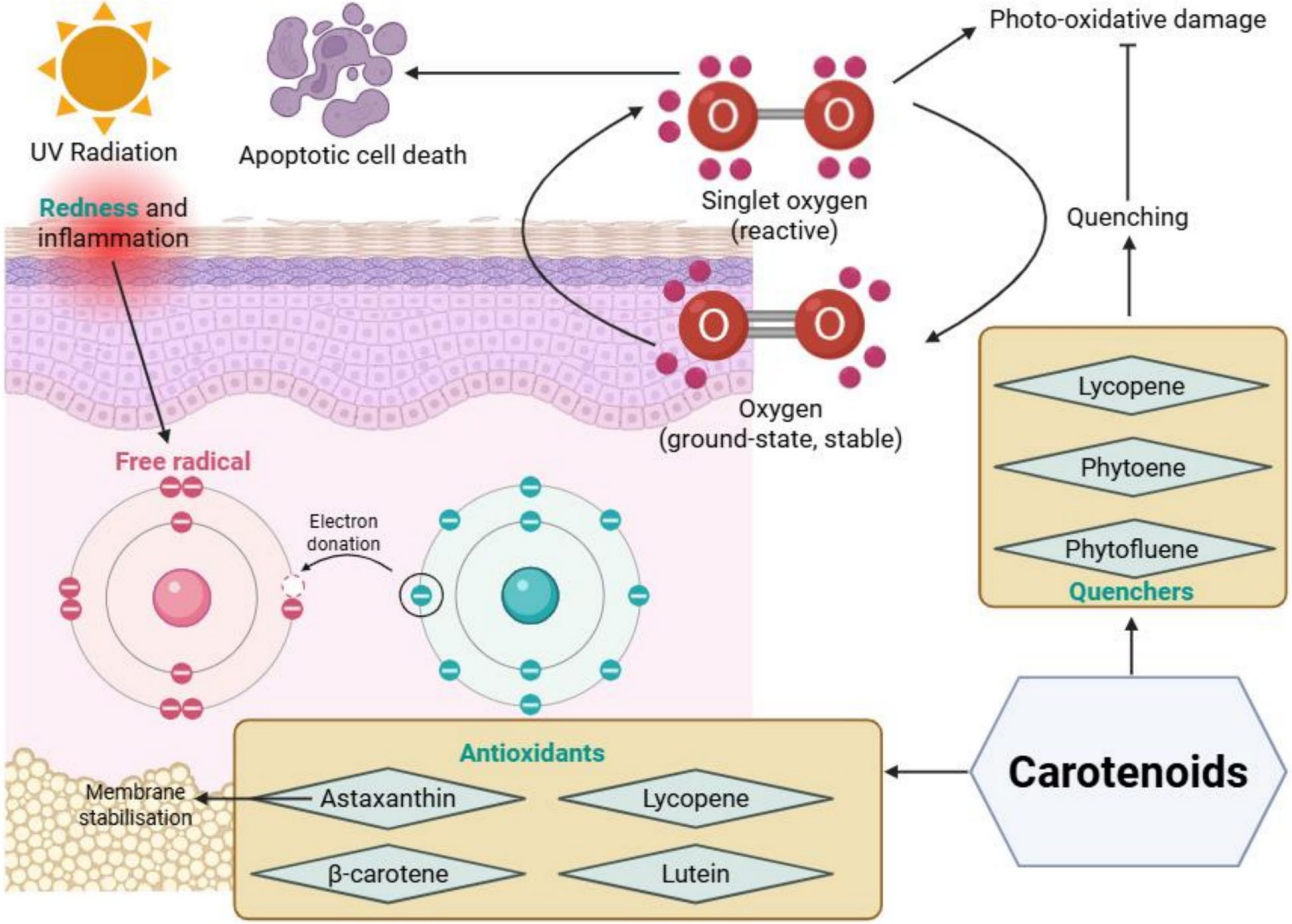
Dietary carotenoids reduce skin redness by providing antioxidant and photoprotective effects. Compounds such as lycopene, phytoene, phytofluene, β-carotene, lutein, and astaxanthin from sources such as tomatoes and Ataulfo mangoes mitigate UV-induced oxidative stress and inflammation, helping to reduce skin redness and photo-oxidative skin damage

Carotenoids, a class of pigmented compounds found in various fruits and vegetables, have beneficial effects in reducing skin redness through their antioxidant and photoprotective properties (Fig. 4).

Ultraviolet radiation from the Sun damages the skin and generates singlet oxygen (i.e., a highly reactive form of oxygen) in the skin. This singlet oxygen damages the skin and has been documented in the literature as a potent trigger for inducing apoptosis in skin-infiltrating T-helper cells [74]. Lycopene, phytoene, and phytofluene from tomato extracts [59] help to protect the skin against photo-oxidative damage by acting as quenchers, converting singlet oxygen back into the more stable ground-state form of oxygen. By doing so, these quenchers help to prevent photo-oxidative damage and preserve skin integrity.

Other carotenoids, including β-carotene [61, 62], lycopene [59, 62, 65, 66], lutein [62, 66], and astaxanthin from tomatoes [63, 65, 66] and Ataulfo mangoes [60], and supplement blends [66] are potent antioxidants. These carotenoids help to reduce skin erythema by mitigating oxidative stress and shielding the skin from ultraviolet-induced inflammation. Astaxanthin, a xanthophyll, also stabilises cell membranes and neutralises free radicals, offering further protection.

The overall effect of incorporating a variety of carotenoids through diet or supplementation is that it reinforces the defences of the skin by removing reactive oxygen species, reduces skin redness, and enhances the resistance of the skin to photo-oxidative damage.

### Collagen supplementation enhances skin hydration and reduces pigment spots

**Fig. 5:**
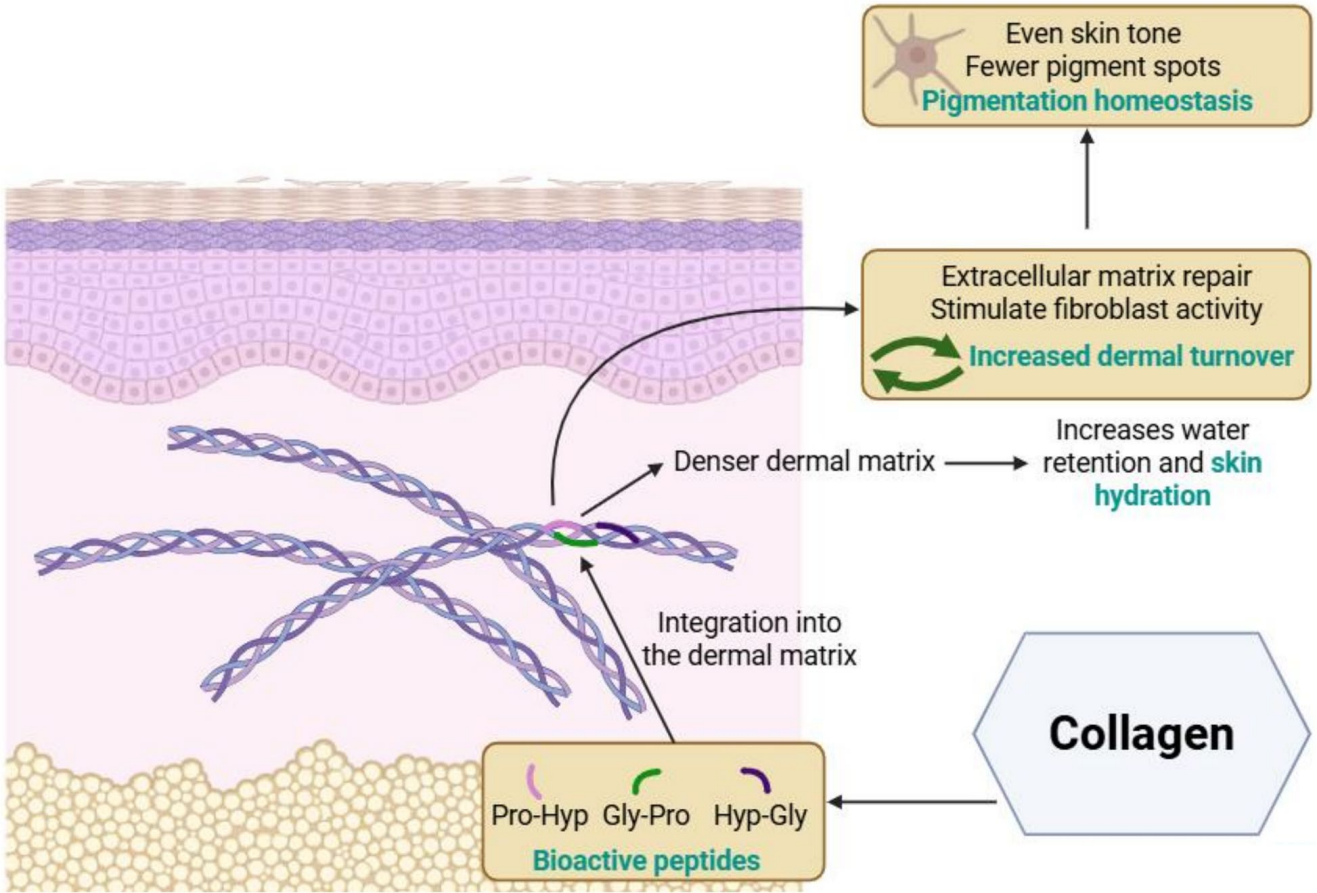
Collagen supplementation and collagen biosynthesis-enhancing extracts improve skin hydration and reduce pigment spots by supporting dermal structure and function. Bioactive peptides from egg membrane, fish, grape skin and Edible Bird’s Nest, along with extracts such as acerola and pine bark, promote extracellular matrix repair and stimulate fibroblast activity. These actions improve moisture retention and contribute to pigmentation homeostasis, leading to a more hydrated and even-toned skin

Collagen supplementation has shown promising benefits for improving skin hydration and reducing pigment spots (Fig. 5). A range of collagen sources, including egg membrane (Ovoderm) [27], fish proteins [23, 34], and Edible Bird’s Nest extract [31, 75], deliver primarily di- and tri-bioactive peptides such as Gly-Pro, Pro-Hyp and Hyp-Gly that integrate directly into the dermal matrix. These peptides act as building blocks that integrate into dermal collagen networks and repair and remodel the extracellular dermal matrix. These peptides also stimulate dermal fibroblast proliferation, promote collagen biosynthesis, leading to an improved dermal matrix density.

By promoting a healthier dermal architecture and stimulating extracellular matrix renewal, collagen peptides may contribute to pigmentation homeostasis by increasing dermal turnover. This could potentially explain the connection between collagen intake and a more even skin tone with fewer pigment spots.

Meanwhile, hydrolysed collagen [23, 32, 36, 37], together with hyaluronans [30, 33] and other important glycosaminoglycans of the extracellular matrix, improve moisture retention and maintain intercellular moisture by enhancing dermal matrix density. This leads to improved skin hydration.

Overall, collagen peptides play a functional role in improving skin hydration and reducing pigment spots through structural and cellular support of the dermis.

### Lipids and fatty acids reduce wrinkling and improves hydration and elasticity

**Fig. 6:**
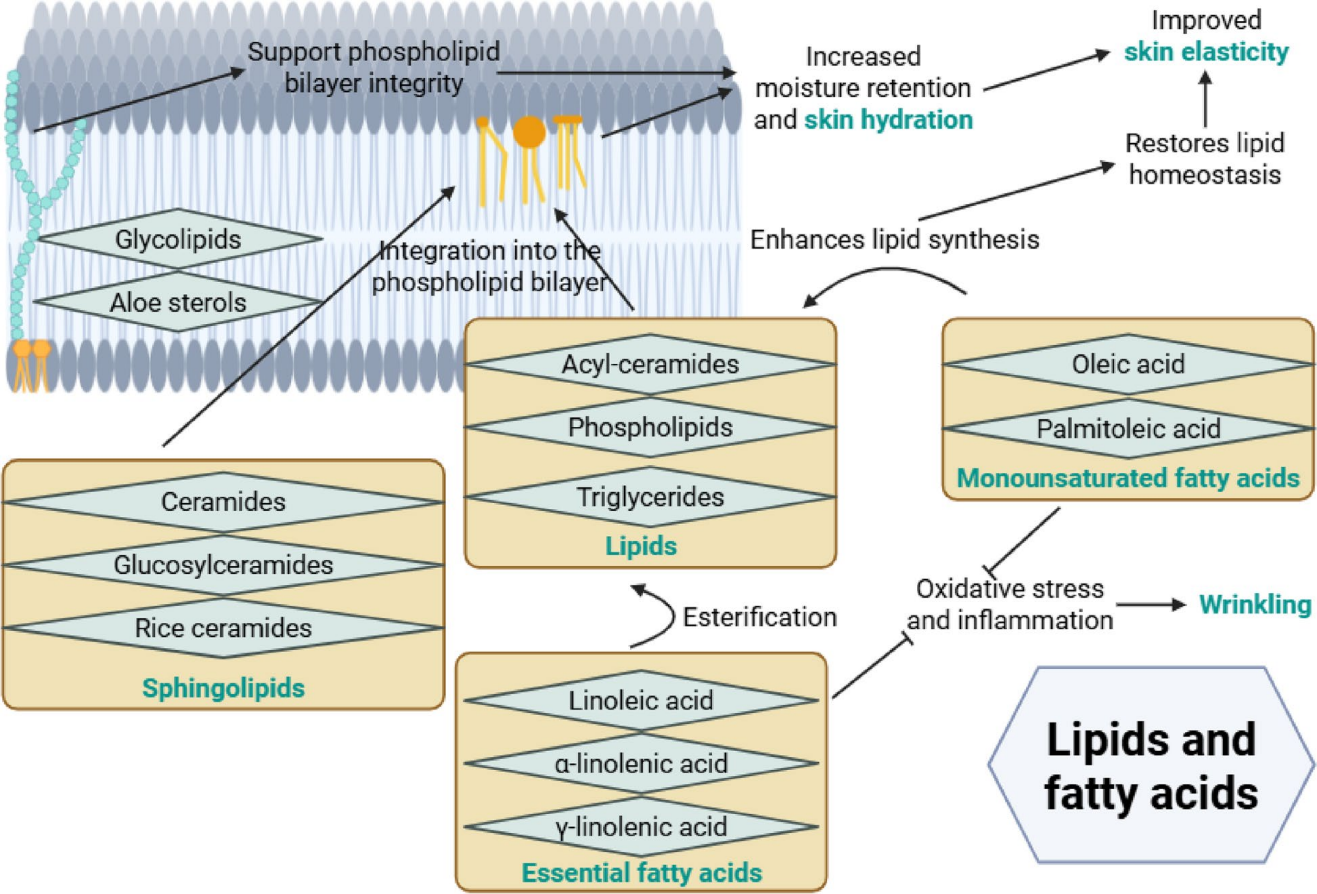
Lipids and fatty acids improve skin wrinkling, hydration, and elasticity by maintaining lipid homeostasis and reducing oxidative stress and inflammation. Essential fatty acids, sphinogolipids, and monounsaturated fatty acids from sources such as borage seed, flaxseed, evening primose, sea buckthorn, torula yeast, avocados, wheat, rice, and corn play important roles to enhance moisture retention, support dermal structure, and improve skin elasticity

Various lipid and fatty acid compounds have shown significant benefits for improving skin wrinkling, hydration, and elasticity, primarily through mechanisms that strengthen the epidermal barrier, modulate inflammation, and restore lipid homeostasis (Fig. 6).

Essential polyunsaturated fatty acids support skin health. Omega-6 linoleic acid, found in flaxseed [38], corn oil [39], and sea buckthorn oil [40], and omega-3 α-linolenic acid mainly from flaxseeds [38] improve epidermal barrier integrity and reduce skin inflammation. Omega-6 γ-linolenic acid, found in borage seed [16, 38] and evening primrose oil [41] has similar effects. Monounsaturated fatty acids also benefit the skin. Palmitoleic acid from 7-MEGA [39] and oleic acid from avocados [42] modulate skin lipid composition and barrier function. These changes enhance barrier permeability and reduce inflammation. Together, monounsaturated and polyunsaturated fatty acids reduce oxidative stress and suppress pro-inflammatory pathways that contribute to wrinkle formation.

The essential fatty acids linoleic acid, α-linolenic acid, and γ-linolenic acid undergo esterification into acyl-ceramides, phospholipids, and triglycerides, respectively [38]. Monounsaturated fatty acids also promote lipid biosynthesis. These acyl-ceramides, phospholipids, and triglycerides are incorporated into the cell membrane. Sphingolipids are also integrated into the membrane; these come from acetic acid bacteria for ceramides [43], torula yeast for glycosylceramides [44], and Asian cultivated rice for rice ceramides [22]. The lipids increase the turnover and renewal of the skin’s lipid structures. The sphingolipids replenish skin ceramides. Together, they enhance the skin barrier, supporting moisture retention and hydration. Plant-derived sterol lipids such as aloe sterols [14], and glycolipids from wheat polar lipid complexes [46], may also be incorporated into the cell membrane. These compounds support the integrity of the phospholipid bilayer and modulate metabolism, further sustaining hydration.

Many of the above-described lipids and fatty acids also support skin elasticity. Essential fatty acids, such as linoleic acid, α-linolenic acid, and γ-linolenic acid, primarily function in biological processes that protect and preserve skin health. They support skin elasticity by strengthening the skin barrier, reducing inflammation and oxidative stress, and preserving the structural integrity of the skin, collectively maintaining flexible and elastic skin. In contrast, sphingolipids, including rice ceramides and glucosylceramides, primarily act by restoring the structural components of the skin. They replenish and boost the skin’s natural ceramide content, strengthening the lipid barrier and enhancing hydration, leading to improved skin smoothness and structural support. Palmitoleic acid activates Sirtuin 1 (SIRT-1), leading to decreased matrix metalloproteinase 1 (MMP-1) expression and increased elastin production [76]. Through these metabolic and signalling pathways, it stimulates cellular regeneration and dynamically maintains skin bounce. Finally, glycolipids contribute to the structural integrity of the skin by maintaining membrane stability and facilitating cell–cell interactions [77]. They play a role in modulating the physical properties of the skin’s lipid bilayer, which is essential for maintaining elasticity. Together, these four groups target distinct but complementary aspects of skin elasticity, collectively improving skin resilience, smoothness, and bounce.

Overall, various lipids and fatty acids improve skin wrinkling, hydration and elasticity by strengthening the skin barrier, reducing inflammation, restoring lipid balance, promoting cellular regeneration and maintaining membrane stability.

### Polyphenols improve skin hydration, strengthen skin barrier and reduce wrinkles through antioxidant and anti-inflammatory mechanisms

**Fig. 7:**
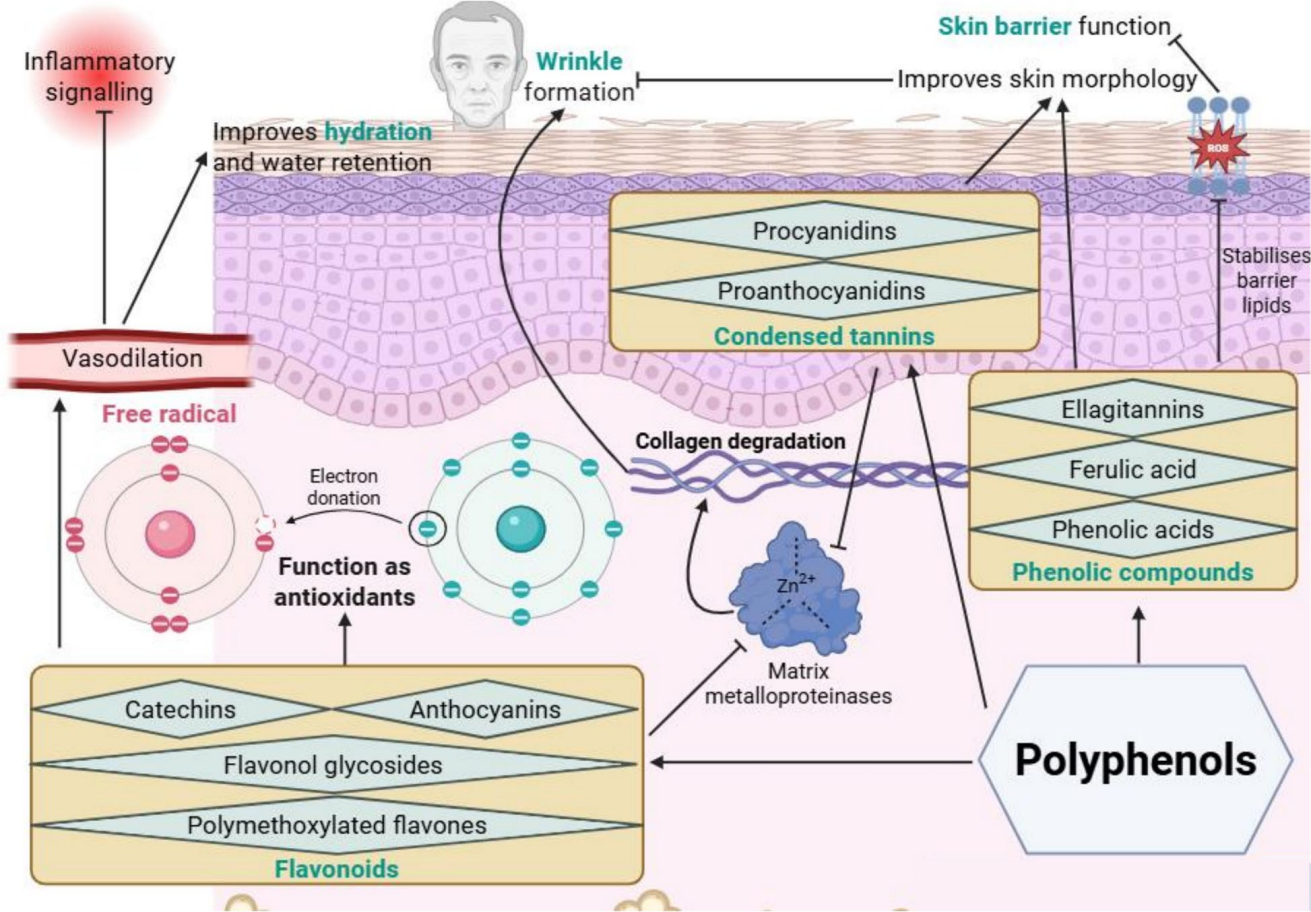
Polyphenols improve skin wrinkling, hydration and barrier function by exerting antioxidant and anti-inflammatory effects. Flavonoids, phenolic compounds, and condensed tannins from sources such as green tea, cocoa, pine bark, grape seed, and citrus fruits reduce wrinkle formation, enhance moisture retention, and lower trans-epidermal water loss (TEWL), contributing to a less wrinkled and more hydrated skin

Dietary polyphenols improve skin hydration, strengthen the skin barrier, and reduce wrinkle formation by acting as antioxidants and stabilising barrier lipids (Fig. 7). These plant-derived compounds can be broadly categorised into three major classes: flavonoids, phenolic compounds, and condensed tannins. Catechins from green tea [17, 47], and catechins and epicatechins from cocoa powder [48], improve microcirculation. This boosts antioxidant defences in the skin and suppresses inflammation, indirectly promoting skin moisture retention. Polymethoxylated flavones from green mandarin extract [49], flavonoid glycosides from Hydrangea serrata [50] and lemon balm tea [51], and anthocyanins from red orange complexes [52] and chokeberries [53], exhibit both antioxidant and anti-inflammatory activities. These actions promote skin moisture retention and improve skin hydration.

Phenolic compounds support skin barrier function and reduce trans-epidermal water loss by acting as strong antioxidants. Ellagitannins from pomegranate [20, 54] and ferulic acid from dietary supplements [13] scavenge free radicals, suppressing lipid peroxidation and limiting damage to membrane phospholipids. Ellagitannins bind to Kelch-like ECH-associated protein 1 (Keap1). This releases nuclear factor erythroid 2–related factor 2 (Nrf2), which then translocates to the nucleus. Ferulic acid indirectly activates Nrf2 by modulating cellular redox status [78]. Once translocated, Nrf2 activates the Nrf2–ARE (antioxidant response element) signalling pathway, inducing the transcription of cytoprotective enzymes, protecting epidermal lipids from oxidative damage [79]. Wheat extracts also provide phenolic acids [46] and these acids reduce inflammation by downregulating pro-inflammatory mediators like cyclooxygenase-2 (COX-2) and nuclear factor kappa-light-chain-enhancer of activated B cells (NF-κB) [80]. Collectively, these compounds help stabilise epidermal lipid structures by protecting them from oxidative damage. The outcome is an improved skin barrier function and reduced moisture loss from the skin.

Polyphenols reduce wrinkling by acting against oxidative stress and inhibiting collagen-degrading matrix metalloproteinases (MMPs). Catechins from green tea and cocoa powder scavenge free radicals [17, 47, 48]. Free radicals would otherwise activate the mitogen-activated protein kinase (MAPK) pathway, leading to upregulated MMPs. By suppressing the MAPK pathway, catechins help to preserve collagen integrity and reduce wrinkle formation. Procyanidins from French maritime pine bark [55] and proanthocyanidins from white tea and grape seed extracts [18] belong to a subclass of polyphenols known as condensed tannins. These condensed tannins inhibit MMP activity, promote collagen stability, and reduce wrinkle formation.

Overall, dietary polyphenols improve skin hydration, barrier integrity, and reduce wrinkle formation by modulating oxidative stress, reducing inflammation, stabilising barrier lipids, and preserving collagen structure.

### Probiotics and prebiotics improve skin hydration by modulating the gut microbiome and gut–skin axis

**Fig. 8:**
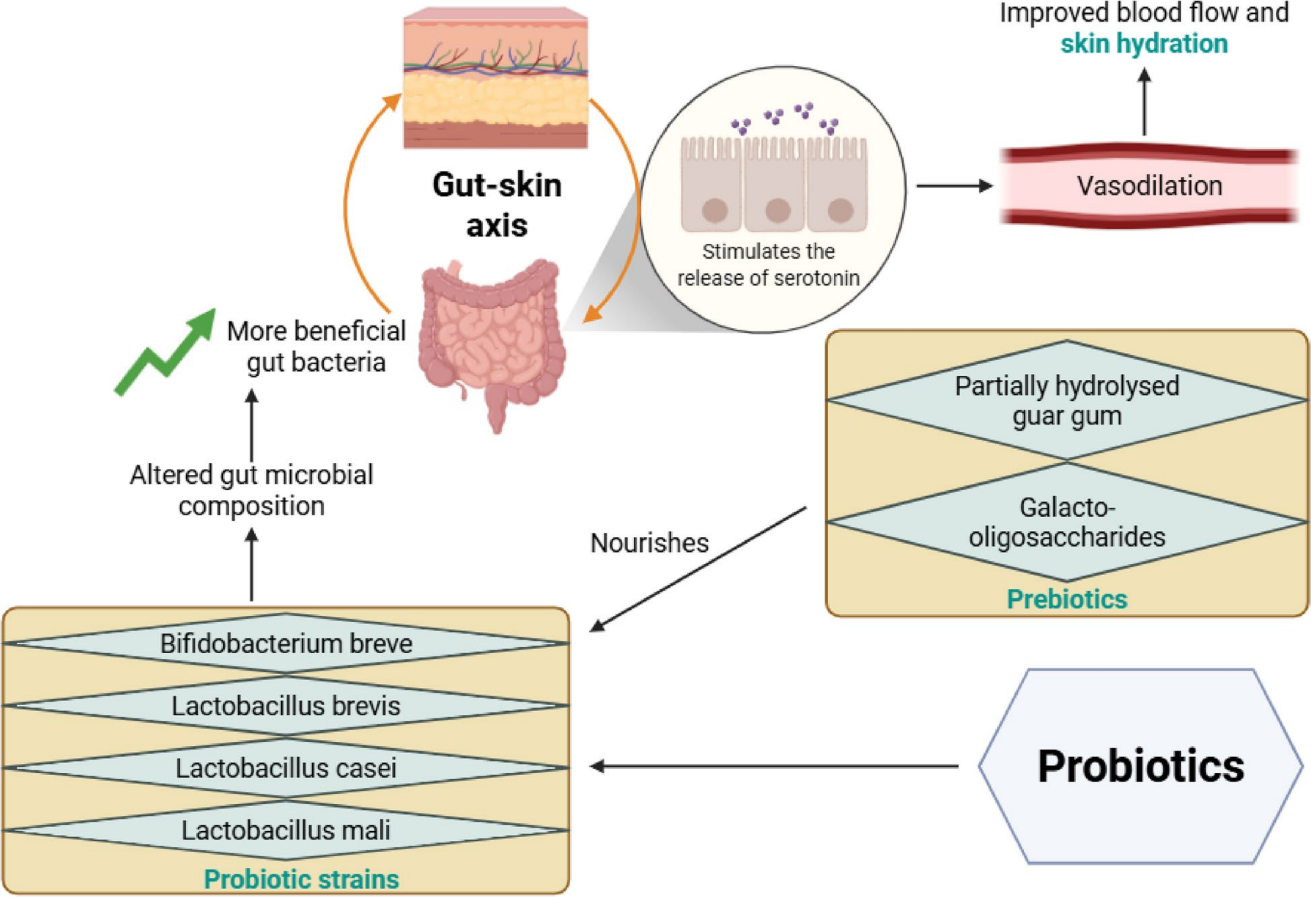
Prebiotics and probiotics improve skin hydration by modulating the gut–skin axis. Beneficial *Bifidobacterium* and *Lactobacillus* live probiotic strains introduced to the gut through fermented soy milk supplemented with prebiotics promote the gut microbial ecosystem and supports skin moisture through the gut–skin axis via systemic effects

Probiotics and prebiotics exert beneficial effects on skin hydration mainly by modulating the gut microbiome and the gut–skin axis. The gut–skin axis is a bidirectional communication network in which gut microbes influence skin health through immune, metabolic, and inflammatory pathways. Several formulations reported in the literature, including live [15, 67, 71, 81] or heat-killed bacteria [72], and the supplementation of prebiotic fibres, have demonstrated improvements in skin moisture levels through gut-mediated mechanisms (Fig. 8).

Galacto-oligosaccharides [69] and partially hydrolysed guar gum [70] are non-digestible dietary fibres that function as prebiotics. They selectively promote beneficial gut bacteria growth and proliferation, thereby altering the microbial composition in favour of a healthier gut ecosystem. As the gut microbiome plays a central role in regulating the gut–skin axis, these microbial shifts may help improve skin hydration.

One possible mechanism on how heat-killed *Lactobacillus brevis* SBC8803 in the gut can indirectly affect the skin has been described in the literature [72]. When in the gut, this bacterium stimulates serotonin release from intestinal enterochromaffin cells. This response increases cutaneous blood flow, thereby reducing skin dryness and improving skin hydration.

Overall, these findings highlight the potential of prebiotic and probiotic strategies to improve skin hydration through targeted modulation of the gut microbiota.

### Limitations

There is substantial heterogeneity across intervention studies due to differences in dosage, duration, and participant ethnicity. This variability complicates meta-analytic data pooling. However, heterogeneity is not inherently negative; it reflects diverse study designs and populations, offering a broader understanding of how dietary factors affect skin ageing under diverse conditions.

Also, as mentioned earlier in *Dietary effects on skin ageing*: *From targeted benefits to broad improvements*, carotenoids and vitamins are among the least studied dietary interventions. No studies have examined carotenoids’ effects on pigment spots, sebum or skin thickness, and evidence on vitamins’ impact on redness, pigment spots, or sebum is limited. These gaps highlight key areas for future research into diet and skin ageing mechanisms.

Most studies focused on female participants, with limited representation of males and diverse ethnic groups. Although analyses are adjusted for ethnicity, population-specific effects remain underexplored. Future studies should address these gaps by investigating more balanced cohorts across sexes and ethnicities to provide a comprehensive understanding of the influence of diet on skin ageing.

Another challenge is that most interventional studies are short-term, often lasting only a few weeks to a few months, and may not capture the long-term effects of diet on skin ageing, which progresses over much longer timescales. To address this, future research could take the form of longitudinal or prospective studies designed to examine dietary patterns and skin ageing phenotypes over extended time periods. Such designs can provide added evidence on whether the benefits of dietary interventions are sustained. Demonstrating long-term efficacy would strengthen dietary recommendations and support the development of effective public health strategies for promoting healthy skin ageing throughout the lifespan.

### Anthropological perspective on ethnic diets

While the findings above are robust, they may be modulated by ethnic diets, which influence nutrient exposure and modulate skin ageing through cultural, physiological, and behavioural means.

From an anthropological perspective, diets are shaped not only by nutrient availability but also by cultural practices, traditional food preparation, and symbolic meanings of food. Although our analyses are adjusted for ethnicity, such adjustments may not fully capture the complex interactions between culture, physiology, and diet. For example, the Mediterranean diet, rich in olive oil, fruits and vegetables, provides antioxidants and anti-inflammatory compounds that support skin health. Traditional Japanese diets, high in fish, seaweed, and fermented foods, may improve wrinkling, hydration, and skin elasticity via omega-3 fatty acids, and through modulating the gut microbiome. Likewise, Nordic diets rich in whole grains, berries, and oily fish contain polyphenols and fatty acids, which may reduce wrinkles, improve skin hydration, increase skin elasticity, and improve the skin barrier.

Cultural attitudes towards ageing and beauty may also shape dietary behaviours, further modulating outcomes. Recognising these physiological and cultural interactions is essential for developing dietary recommendations that are evidence-based and culturally sensitive.

### Future perspectives

While this review has examined the effects of individual dietary groups, it is important to acknowledge that nutrients are not consumed in isolation. Instead, in everyday life, foods are eaten in combinations that form habitual dietary patterns, with certain food items regularly appearing together. Our previous work has identified such patterns, and future research will build on these findings to investigate how different dietary patterns are associated with skin ageing phenotypes.

Beyond dietary patterns, further research could explore the potential of personalised nutrition approaches. Genetic predispositions, microbiome composition, and lifestyle factors may interact with dietary interventions to influence skin ageing outcomes. Identifying gene–diet interactions and tailoring interventions accordingly could allow for more targeted and effective strategies.

A deeper understanding of the role of diet in skin ageing could inform practical guidelines for the general population and support public health policies that integrate skin health into broader nutritional recommendations. Such initiatives may help bridge the gap between clinical findings and everyday practice and could ultimately inform the development of evidence-based nutritional strategies and public health policies aimed at promoting healthy skin ageing.

## Conclusion

In conclusion, this systematic review and meta-analysis synthesises current evidence on a range of dietary interventions and examines the mechanisms in which they work to promote healthy skin ageing. These interventions have been shown to slow the progression of key skin ageing parameters, including wrinkle formation, skin redness, and pigment spots, while also enhancing skin hydration, barrier function, and elasticity.

Carotenoids, collagen peptides, lipids and fatty acids, polyphenols, and probiotics each contribute to skin health through distinct yet complementary mechanisms. Carotenoids enhance the skin’s defence against photo-oxidative damage by neutralising reactive oxygen species and reducing erythema, thereby diminishing visible skin redness. Collagen peptides support dermal architecture, resulting in improved hydration and a reduction in pigment spots. Lipids and fatty acids strengthen the skin barrier, modulate inflammatory responses and restore lipid homeostasis, leading to improved skin hydration, elasticity, and fewer wrinkles. Polyphenols act as antioxidants and skin barrier stabilisers, thereby improving skin hydration, improve barrier integrity, and reducing wrinkle formation. Finally, probiotic and prebiotic interventions improve skin moisture levels through modulating the gut–skin axis and by fostering the growth of beneficial gut microbiota.

These findings collectively highlight the interconnectivity of different dietary interventions in addressing the most visible and functionally significant features of skin ageing.

Notably, our meta-analytic findings reveal that some dietary interventions are more effective for specific skin ageing outcomes. For instance, carotenoids are effective for reducing skin redness and probiotics are effective for improving skin hydration. This highlights the importance of a comprehensive and integrative approach to dietary strategies targeting skin health.

## Supplementary Information

Additional file 1: PRISMA 2020 for Abstracts Checklist.

Additional file 2: PRISMA 2020 Checklist.

Additional file 3: Summary of dietary intervention studies included in the meta-analysis, alongside their categorisation into dietary groups. Each entry details the original study’s authorship, year, intervention, and the diet category assigned for analysis. Justifications for diet groupings are provided, including the rationale, source, and full citation of the supporting literature used to classify the intervention.

Additional file 4: Extracted study characteristics, participant demographics, dietary intervention details, and outcome measures for all included studies examining the effects of dietary interventions on skin ageing phenotypes. Information includes study design, setting, sample composition, intervention specifics, phenotype definitions, and measurement methods.

Additional file 5: Begg’s funnel plots and Egger’s test *p*-values for detecting publication bias following dietary intervention. Funnel plots show Standardised Mean Differences (SMD) plotted against standard errors (SE) for dietary interventions with two or more studies. Funnel plots could not be drawn for interventions with fewer than two studies and are labelled as ‘Insufficient studies available’. Studies assess changes in wrinkles, hydration, redness, pigment spots, elasticity, the skin barrier, sebum production, and skin thickness **following dietary intervention**. The dietary groups are carotenoids, collagen, lipids and fatty acids, polyphenols, prebiotics and probiotics, and vitamins. Each dot represents a study. The vertical black dotted line represents the pooled effects size measured as pooled Odds Ratios while the diagonal black dotted lines represent the pooled 95% Confidence Interval. Studies with smaller standard errors appear higher in each funnel plot while studies with larger standard errors are positioned lower. Symmetry around the pooled effect estimate suggests the absence of publication bias, while asymmetry may indicate potential publication bias. Funnel plot symmetry is statistically quantified using Egger’s test, where two-tailed *p*-values lower than 0.05 suggest potential publication bias. Egger’s test *p*-values could only be calculated when interventions have three or more studies. SMD: standardised means difference. SE: standard error.

Additional file 6: Begg’s funnel plots and Egger’s test *p*-values for detecting publication bias between dietary interventions and non-interventional controls. Funnel plots show Standardised Mean Differences (SMD) plotted against standard errors (SE) for dietary interventions with two or more studies. Funnel plots could not be drawn for interventions with fewer than two studies and are labelled as ‘Insufficient studies available’. Studies assess changes in wrinkles, hydration, redness, pigment spots, elasticity, the skin barrier, sebum production, and skin thickness **between dietary interventions and non-interventional controls**. The dietary groups are carotenoids, collagen, lipids and fatty acids, polyphenols, prebiotics and probiotics, and vitamins. Each dot represents a study. The vertical black dotted line represents the pooled effects size measured as pooled Odds Ratios while the diagonal black dotted lines represent the pooled 95% Confidence Interval. Studies with smaller standard errors appear higher in each funnel plot while studies with larger standard errors are positioned lower. Symmetry around the pooled effect estimate suggests the absence of publication bias, while asymmetry may indicate potential publication bias. Funnel plot symmetry is statistically quantified using Egger’s test, where two-tailed*p*-values lower than 0.05 suggest potential publication bias. Egger’s test*p*-values could only be calculated when interventions have three or more studies. SMD: standardised means difference. SE: standard error.

Additional file 7: Diversity and scope of publications investigating dietary interventions on skin ageing. The figure summarises the range of published studies assessing different skin ageing parameters following dietary interventions, highlighting both the breadth of existing research and potential gaps in the literature. Y: Yes, the phenotype has been studied; N: No, the phenotype has not been studied.

Additional file 8: Diversity and scope of publications investigating dietary interventions on skin ageing, stratified by dietary interventions. Publications are stratified by skin ageing parameter (rows) and dietary intervention (columns). Each field indicates the presence or absence, and the number of studies assessing each parameter-intervention pair. Fields with no study available are shaded in pink. This figure quantifies research coverage and highlights gaps in the existing literature.

Additional file 9: Forest plot summarising the effect sizes, quantified as Standardised Means Difference (SMD), for studies assessing the impact of dietary interventions on **sebum production** compared to before the dietary intervention. Each circle represents a study's effect size. The size of each circle is proportional to its weight in the meta-analysis. Horizontal lines denote 95% confidence intervals. SMD for each study was calculated using Cohen’s d for paired samples (i.e., before vs after dietary intervention). The vertical dotted line indicates the line of no effect (SMD=0). A positive SMD indicates that the dietary intervention favours more sedum production when compared to before the dietary intervention. The pooled effect estimate and pooled 95% Confidence Interval (CI) are computed based on a Random Effects Model and shown as a diamond, in which the diamond’s width represents the range of the 95% CI. The I² statistic quantifies the proportion of total variation in results across studies investigating the same dietary intervention that is due to heterogeneity rather than chance. An I^2^ value of 0% indicates no observed heterogeneity; the group of studies examining this dietary intervention are relatively homogeneous. Larger I^2^ values indicate greater heterogeneity. SMD: standardised means difference. CI: confidence interval. χ^2^: chi-square. df: degrees of freedom. p: chi-square test *p*-value. I^2^: heterogeneity statistic.

Additional file 10: Forest plot summarising the effect sizes, quantified as Standardised Means Difference (SMD), for studies assessing the impact of dietary interventions on **skin thickness** compared to before the dietary intervention. Each circle represents a study's effect size. The size of each circle is proportional to its weight in the meta-analysis. Horizontal lines denote 95% confidence intervals. SMD for each study was calculated using Cohen’s d for paired samples (i.e., before vs after dietary intervention). The vertical dotted line indicates the line of no effect (SMD=0). A positive SMD indicates that the dietary intervention favours a greater skin thickness when compared to before the dietary intervention. The pooled effect estimate and pooled 95% Confidence Interval (CI) are computed based on a Random Effects Model and shown as a diamond, in which the diamond’s width represents the range of the 95% CI. The I^2^ statistic quantifies the proportion of total variation in results across studies investigating the same dietary intervention that is due to heterogeneity rather than chance. An I^2^ value of 0% indicates no observed heterogeneity; the group of studies examining this dietary intervention are relatively homogeneous. Larger I^2^ values indicate greater heterogeneity. SMD: standardised means difference. CI: confidence interval. χ^2^: chi-square. df: degrees of freedom. p: chi-square test *p*-value. I^2^: heterogeneity statistic.

Additional file 11: Forest plot summarising the effect sizes, quantified as Standardised Means Difference (SMD), for studies assessing the impact of dietary interventions on **skin distensibility** (i.e., R-parameter R0 on the Cutometer) compared to before the dietary intervention. Each circle represents a study's effect size. The size of each circle is proportional to its weight in the meta-analysis. Horizontal lines denote 95% confidence intervals. SMD for each study was calculated using Cohen’s d for paired samples (i.e., before vs after dietary intervention). The vertical dotted line indicates the line of no effect (SMD=0). A negative SMD indicates that the dietary intervention favours a less saggy skin (i.e., less skin distensibility, R-parameter R0 on the Cutometer) when compared to before the dietary intervention. The pooled effect estimate and pooled 95% Confidence Interval (CI) are computed based on a Random Effects Model and shown as a diamond, in which the diamond’s width represents the range of the 95% CI. The I^2^ statistic quantifies the proportion of total variation in results across studies investigating the same dietary intervention that is due to heterogeneity rather than chance. An I^2^ value of 0% indicates no observed heterogeneity; the group of studies examining this dietary intervention are relatively homogeneous. Larger I^2^ values indicate greater heterogeneity. SMD: standardised means difference. CI: confidence interval. χ^2^: chi-square. df: degrees of freedom. p: chi-square test*p*-value. I^2^: heterogeneity statistic.

Additional file 12: Forest plot summarising the effect sizes, quantified as Standardised Means Difference (SMD), for studies assessing the impact of dietary interventions on the **overall visco-elasticity** of the skin (i.e., R-parameter R2 on the Cutometer) compared to before the dietary intervention. Each circle represents a study's effect size. The size of each circle is proportional to its weight in the meta-analysis. Horizontal lines denote 95% confidence intervals. SMD for each study was calculated using Cohen’s d for paired samples (i.e., before vs after dietary intervention). The vertical dotted line indicates the line of no effect (SMD=0). A positive SMD indicates that the dietary intervention favours a greater overall visco-elasticity of the skin (i.e., R-parameter R2 on the Cutometer) when compared to before the dietary intervention. The pooled effect estimate and pooled 95% Confidence Interval (CI) are computed based on a Random Effects Model and shown as a diamond, in which the diamond’s width represents the range of the 95% CI. The I^2^ statistic quantifies the proportion of total variation in results across studies investigating the same dietary intervention that is due to heterogeneity rather than chance. An I^2^ value of 0% indicates no observed heterogeneity; the group of studies examining this dietary intervention are relatively homogeneous. Larger I^2^ values indicate greater heterogeneity. SMD: standardised means difference. CI: confidence interval. χ^2^: chi-square. df: degrees of freedom. p: chi-square test *p*-value. I^2^: heterogeneity statistic.

Additional file 13: Forest plot summarising the effect sizes, quantified as Standardised Means Difference (SMD), for studies assessing the impact of dietary interventions on the **net elasticity** of the skin (i.e., R-parameter R5 on the Cutometer) compared to before the dietary intervention. Each circle represents a study's effect size. The size of each circle is proportional to its weight in the meta-analysis. Horizontal lines denote 95% confidence intervals. SMD for each study was calculated using Cohen’s d for paired samples (i.e., before vs after dietary intervention). The vertical dotted line indicates the line of no effect (SMD=0). A positive SMD indicates that the dietary intervention favours a greater net elasticity of the skin (i.e., R-parameter R5 on the Cutometer) when compared to before the dietary intervention. The pooled effect estimate and pooled 95% Confidence Interval (CI) are computed based on a Random Effects Model and shown as a diamond, in which the diamond’s width represents the range of the 95% CI. The I^2^ statistic quantifies the proportion of total variation in results across studies investigating the same dietary intervention that is due to heterogeneity rather than chance. An I^2^ value of 0% indicates no observed heterogeneity; the group of studies examining this dietary intervention are relatively homogeneous. Larger I^2^ values indicate greater heterogeneity. SMD: standardised means difference. CI: confidence interval. χ^2^: chi-square. df: degrees of freedom. p: chi-square test *p*-value. I^2^: heterogeneity statistic.

Additional file 14: Forest plot summarising the effect sizes, quantified as Standardised Means Difference (SMD), for studies assessing the impact of dietary interventions on the **immediate recovery of the skin after suction** (i.e., R-parameter R7 on the Cutometer) compared to before the dietary intervention. Each circle represents a study's effect size. The size of each circle is proportional to its weight in the meta-analysis. Horizontal lines denote 95% confidence intervals. SMD for each study was calculated using Cohen’s d for paired samples (i.e., before vs after dietary intervention). The vertical dotted line indicates the line of no effect (SMD=0). A positive SMD indicates that the dietary intervention favours a greater immediate recovery of the skin after suction (i.e., R-parameter R7 on the Cutometer) when compared to before the dietary intervention. The pooled effect estimate and pooled 95% Confidence Interval (CI) are computed based on a Random Effects Model and shown as a diamond, in which the diamond’s width represents the range of the 95% CI. The I^2^ statistic quantifies the proportion of total variation in results across studies investigating the same dietary intervention that is due to heterogeneity rather than chance. An I^2^ value of 0% indicates no observed heterogeneity; the group of studies examining this dietary intervention are relatively homogeneous. Larger I^2^ values indicate greater heterogeneity. SMD: standardised means difference. SMD: standardised means difference. CI: confidence interval. χ^2^: chi-square. df: degrees of freedom. p: chi-square test *p*-value. I^2^: heterogeneity statistic.

Additional file 15: Forest plot summarising the effect sizes, quantified as Standardised Means Difference (SMD), for studies assessing the impact of dietary interventions on the **fatigue resistance** of the skin (i.e., R-parameter R9 on the Cutometer) compared to before the dietary intervention. Each circle represents a study's effect size. The size of each circle is proportional to its weight in the meta-analysis. Horizontal lines denote 95% confidence intervals. SMD for each study was calculated using Cohen’s d for paired samples (i.e., before vs after dietary intervention). The vertical dotted line indicates the line of no effect (SMD=0). A positive SMD indicates that the dietary intervention favours a greater fatigue resistance (i.e., R-parameter R9 on the Cutometer) when compared to before the dietary intervention. The pooled effect estimate and pooled 95% Confidence Interval (CI) are computed based on a Random Effects Model and shown as a diamond, in which the diamond’s width represents the range of the 95% CI. The I^2^ statistic quantifies the proportion of total variation in results across studies investigating the same dietary intervention that is due to heterogeneity rather than chance. An I^2^ value of 0% indicates no observed heterogeneity; the group of studies examining this dietary intervention are relatively homogeneous. Larger I^2^ values indicate greater heterogeneity. SMD: standardised means difference. CI: confidence interval. χ^2^: chi-square. df: degrees of freedom. p: chi-square test *p*-value. I^2^: heterogeneity statistic.

Additional file 16: Forest plot summarising the effect sizes, quantified as Standardised Means Difference (SMD), for studies assessing the impact of dietary interventions on **wrinkles compared to non-interventional controls**. Each circle represents a study's effect size. The size of each circle is proportional to its weight in the meta-analysis. Horizontal lines denote 95% confidence intervals. SMD for each study was calculated using Cohen’s d for paired samples (i.e., before vs after dietary intervention). The vertical dotted line indicates the line of no effect (SMD=0). A negative SMD indicates that the dietary intervention favours fewer wrinkles when compared to non-interventional controls. The pooled effect estimate and pooled 95% Confidence Interval (CI) are computed based on a Random Effects Model and shown as a diamond, in which the diamond’s width represents the range of the 95% CI. The I^2^ statistic quantifies the proportion of total variation in results across studies investigating the same dietary intervention that is due to heterogeneity rather than chance. An I^2^ value of 0% indicates no observed heterogeneity; the group of studies examining this dietary intervention are relatively homogeneous. Larger I^2^ values indicate greater heterogeneity. SMD: standardised means difference. CI: confidence interval. χ^2^: chi-square. df: degrees of freedom. p: chi-square test *p*-value. I^2^: heterogeneity statistic.

Additional file 17: Forest plot summarising the effect sizes, quantified as Standardised Means Difference (SMD), for studies assessing the impact of dietary interventions on **skin hydration compared to non-interventional controls**. Each circle represents a study's effect size. The size of each circle is proportional to its weight in the meta-analysis. Horizontal lines denote 95% confidence intervals. SMD for each study was calculated using Cohen’s d for paired samples (i.e., before vs after dietary intervention). The vertical dotted line indicates the line of no effect (SMD=0). A positive SMD indicates that the dietary intervention favours a higher skin hydration when compared to non-interventional controls. The pooled effect estimate and pooled 95% Confidence Interval (CI) are computed based on a Random Effects Model and shown as a diamond, in which the diamond’s width represents the range of the 95% CI. The I^2^ statistic quantifies the proportion of total variation in results across studies investigating the same dietary intervention that is due to heterogeneity rather than chance. An I^2^ value of 0% indicates no observed heterogeneity; the group of studies examining this dietary intervention are relatively homogeneous. Larger I^2^ values indicate greater heterogeneity. SMD: standardised means difference. CI: confidence interval. χ^2^: chi-square. df: degrees of freedom. p: chi-square test *p*-value. I^2^: heterogeneity statistic.

Additional file 18: Forest plot summarising the effect sizes, quantified as Standardised Means Difference (SMD), for studies assessing the impact of dietary interventions on **skin redness compared to non-interventional controls**. Each circle represents a study's effect size. The size of each circle is proportional to its weight in the meta-analysis. Horizontal lines denote 95% confidence intervals. SMD for each study was calculated using Cohen’s d for paired samples (i.e., before vs after dietary intervention). The vertical dotted line indicates the line of no effect (SMD=0). A negative SMD indicates that the dietary intervention favours a lower skin redness when compared to non-interventional controls. The pooled effect estimate and pooled 95% Confidence Interval (CI) are computed based on a Random Effects Model and shown as a diamond, in which the diamond’s width represents the range of the 95% CI. The I^2^ statistic quantifies the proportion of total variation in results across studies investigating the same dietary intervention that is due to heterogeneity rather than chance. An I^2^ value of 0% indicates no observed heterogeneity; the group of studies examining this dietary intervention are relatively homogeneous. Larger I^2^ values indicate greater heterogeneity. SMD: standardised means difference. CI: confidence interval. χ^2^: chi-square. df: degrees of freedom. p: chi-square test *p*-value. I^2^: heterogeneity statistic.

Additional file 19: Forest plot summarising the effect sizes, quantified as Standardised Means Difference (SMD), for studies assessing the impact of dietary interventions on **pigment spots compared to non-interventional controls**. Each circle represents a study's effect size. The size of each circle is proportional to its weight in the meta-analysis. Horizontal lines denote 95% confidence intervals. SMD for each study was calculated using Cohen’s d for paired samples (i.e., before vs after dietary intervention). The vertical dotted line indicates the line of no effect (SMD=0). A negative SMD indicates that the dietary intervention favours pigment spots when compared to non-interventional controls. The pooled effect estimate and pooled 95% Confidence Interval (CI) are computed based on a Random Effects Model and shown as a diamond, in which the diamond’s width represents the range of the 95% CI. The I^2^ statistic quantifies the proportion of total variation in results across studies investigating the same dietary intervention that is due to heterogeneity rather than chance. An I^2^ value of 0% indicates no observed heterogeneity; the group of studies examining this dietary intervention are relatively homogeneous. Larger I^2^ values indicate greater heterogeneity. SMD: standardised means difference. CI: confidence interval. χ^2^: chi-square. df: degrees of freedom. p: chi-square test *p*-value. I^2^: heterogeneity statistic.

Additional file 20: Forest plot summarising the effect sizes, quantified as Standardised Means Difference (SMD), for studies assessing the impact of dietary interventions on **skin elasticity compared to non-interventional controls**. Each circle represents a study's effect size. The size of each circle is proportional to its weight in the meta-analysis. Horizontal lines denote 95% confidence intervals. SMD for each study was calculated using Cohen’s d for paired samples (i.e., before vs after dietary intervention). The vertical dotted line indicates the line of no effect (SMD=0). A positive SMD indicates that the dietary intervention favours greater skin elasticity when compared to non-interventional controls. The pooled effect estimate and pooled 95% Confidence Interval (CI) are computed based on a Random Effects Model and shown as a diamond, in which the diamond’s width represents the range of the 95% CI. The I^2^ statistic quantifies the proportion of total variation in results across studies investigating the same dietary intervention that is due to heterogeneity rather than chance. An I^2^ value of 0% indicates no observed heterogeneity; the group of studies examining this dietary intervention are relatively homogeneous. Larger I^2^ values indicate greater heterogeneity. SMD: standardised means difference. CI: confidence interval. χ^2^: chi-square. df: degrees of freedom. p: chi-square test *p*-value. I^2^: heterogeneity statistic.

Additional file 21: Forest plot summarising the effect sizes, quantified as Standardised Means Difference (SMD), for studies assessing the impact of dietary interventions on the **skin barrier compared to non-interventional controls**. Each circle represents a study's effect size. The size of each circle is proportional to its weight in the meta-analysis. Horizontal lines denote 95% confidence intervals. SMD for each study was calculated using Cohen’s d for paired samples (i.e., before vs after dietary intervention). The vertical dotted line indicates the line of no effect (SMD=0). A negative SMD indicates that the dietary intervention favours a lower trans-epidermal water loss (TEWL) (i.e., a better skin barrier) when compared to non-interventional controls. The pooled effect estimate and pooled 95% Confidence Interval (CI) are computed based on a Random Effects Model and shown as a diamond, in which the diamond’s width represents the range of the 95% CI. The I^2^ statistic quantifies the proportion of total variation in results across studies investigating the same dietary intervention that is due to heterogeneity rather than chance. An I^2^ value of 0% indicates no observed heterogeneity; the group of studies examining this dietary intervention are relatively homogeneous. Larger I^2^ values indicate greater heterogeneity. SMD: standardised means difference. CI: confidence interval. χ^2^: chi-square. df: degrees of freedom. p: chi-square test *p*-value. I^2^: heterogeneity statistic. TEWL: trans-epidermal water loss.

Additional file 22: Forest plot summarising the effect sizes, quantified as Standardised Means Difference (SMD), for studies assessing the impact of dietary interventions on **sebum production compared to non-interventional controls**. Each circle represents a study's effect size. The size of each circle is proportional to its weight in the meta-analysis. Horizontal lines denote 95% confidence intervals. SMD for each study was calculated using Cohen’s d for paired samples (i.e., before vs after dietary intervention). The vertical dotted line indicates the line of no effect (SMD=0). A positive SMD indicates that the dietary intervention favours more sebum production when compared to non-interventional controls. The pooled effect estimate and pooled 95% Confidence Interval (CI) are computed based on a Random Effects Model and shown as a diamond, in which the diamond’s width represents the range of the 95% CI. The I^2^ statistic quantifies the proportion of total variation in results across studies investigating the same dietary intervention that is due to heterogeneity rather than chance. An I^2^ value of 0% indicates no observed heterogeneity; the group of studies examining this dietary intervention are relatively homogeneous. Larger I^2^ values indicate greater heterogeneity. SMD: standardised means difference. CI: confidence interval. χ^2^: chi-square. df: degrees of freedom. p: chi-square test *p*-value. I^2^: heterogeneity statistic.

Additional file 23: Forest plot summarising the effect sizes, quantified as Standardised Means Difference (SMD), for studies assessing the impact of dietary interventions on **skin thickness compared to non-interventional controls**. Each circle represents a study's effect size. The size of each circle is proportional to its weight in the meta-analysis. Horizontal lines denote 95% confidence intervals. SMD for each study was calculated using Cohen’s d for paired samples (i.e., before vs after dietary intervention). The vertical dotted line indicates the line of no effect (SMD=0). A positive SMD indicates that the dietary intervention favours a greater skin thickness when compared to non-interventional controls. The pooled effect estimate and pooled 95% Confidence Interval (CI) are computed based on a Random Effects Model and shown as a diamond, in which the diamond’s width represents the range of the 95% CI. The I^2^ statistic quantifies the proportion of total variation in results across studies investigating the same dietary intervention that is due to heterogeneity rather than chance. An I^2^ value of 0% indicates no observed heterogeneity; the group of studies examining this dietary intervention are relatively homogeneous. Larger I^2^ values indicate greater heterogeneity. SMD: standardised means difference. CI: confidence interval. χ^2^: chi-square. df: degrees of freedom. p: chi-square test *p*-value. I^2^: heterogeneity statistic.

Additional file 24: Forest plot summarising the effect sizes, quantified as Standardised Means Difference (SMD), for studies assessing the impact of dietary interventions on **skin distensibility (i.e., R-parameter R0 on the Cutometer) compared to non-interventional controls**. Each circle represents a study's effect size. The size of each circle is proportional to its weight in the meta-analysis. Horizontal lines denote 95% confidence intervals. SMD for each study was calculated using Cohen’s d for paired samples (i.e., before vs after dietary intervention). The vertical dotted line indicates the line of no effect (SMD=0). A negative SMD indicates that the dietary intervention favours a less saggy skin (i.e., less skin distensibility, R-parameter R0 on the Cutometer) when compared to non-interventional controls. The pooled effect estimate and pooled 95% Confidence Interval (CI) are computed based on a Random Effects Model and shown as a diamond, in which the diamond’s width represents the range of the 95% CI. The I^2^ statistic quantifies the proportion of total variation in results across studies investigating the same dietary intervention that is due to heterogeneity rather than chance. An I^2^ value of 0% indicates no observed heterogeneity; the group of studies examining this dietary intervention are relatively homogeneous. Larger I^2^ values indicate greater heterogeneity. SMD: standardised means difference. CI: confidence interval. χ^2^: chi-square. df: degrees of freedom. p: chi-square test *p*-value. I^2^: heterogeneity statistic.

Additional file 25: Forest plot summarising the effect sizes, quantified as Standardised Means Difference (SMD), for studies assessing the impact of dietary interventions on the **overall visco-elasticity of the skin (i.e., R-parameter R2 on the Cutometer) compared to non-interventional controls**. Each circle represents a study's effect size. The size of each circle is proportional to its weight in the meta-analysis. Horizontal lines denote 95% confidence intervals. SMD for each study was calculated using Cohen’s d for paired samples (i.e., before vs after dietary intervention). The vertical dotted line indicates the line of no effect (SMD=0). A positive SMD indicates that the dietary intervention favours a greater overall visco-elasticity of the skin (i.e., R-parameter R2 on the Cutometer) when compared to non-interventional controls. The pooled effect estimate and pooled 95% Confidence Interval (CI) are computed based on a Random Effects Model and shown as a diamond, in which the diamond’s width represents the range of the 95% CI. The I^2^ statistic quantifies the proportion of total variation in results across studies investigating the same dietary intervention that is due to heterogeneity rather than chance. An I^2^ value of 0% indicates no observed heterogeneity; the group of studies examining this dietary intervention are relatively homogeneous. Larger I^2^ values indicate greater heterogeneity. SMD: standardised means difference. CI: confidence interval. χ^2^: chi-square. df: degrees of freedom. p: chi-square test *p*-value. I^2^: heterogeneity statistic.

Additional file 26: Forest plot summarising the effect sizes, quantified as Standardised Means Difference (SMD), for studies assessing the impact of dietary interventions on the **net elasticity of the skin (i.e., R-parameter R5 on the Cutometer) compared to non-interventional controls**. Each circle represents a study's effect size. The size of each circle is proportional to its weight in the meta-analysis. Horizontal lines denote 95% confidence intervals. SMD for each study was calculated using Cohen’s d for paired samples (i.e., before vs after dietary intervention). The vertical dotted line indicates the line of no effect (SMD=0). A positive SMD indicates that the dietary intervention favours a greater net elasticity of the skin (i.e., R-parameter R5 on the Cutometer) when compared to non-interventional controls. The pooled effect estimate and pooled 95% Confidence Interval (CI) are computed based on a Random Effects Model and shown as a diamond, in which the diamond’s width represents the range of the 95% CI. The I^2^ statistic quantifies the proportion of total variation in results across studies investigating the same dietary intervention that is due to heterogeneity rather than chance. An I^2^ value of 0% indicates no observed heterogeneity; the group of studies examining this dietary intervention are relatively homogeneous. Larger I^2^ values indicate greater heterogeneity. SMD: standardised means difference. CI: confidence interval. χ^2^: chi-square. df: degrees of freedom. p: chi-square test *p*-value. I^2^: heterogeneity statistic.

Additional file 27: Forest plot summarising the effect sizes, quantified as Standardised Means Difference (SMD), for studies assessing the impact of dietary interventions on the **immediate recovery of the skin after suction (i.e., R-parameter R7 on the Cutometer) compared to non-interventional controls**. Each circle represents a study's effect size. The size of each circle is proportional to its weight in the meta-analysis. Horizontal lines denote 95% confidence intervals. SMD for each study was calculated using Cohen’s d for paired samples (i.e., before vs after dietary intervention). The vertical dotted line indicates the line of no effect (SMD=0). A positive SMD indicates that the dietary intervention favours a greater immediate recovery of the skin after suction (i.e., R-parameter R7 on the Cutometer) when compared to non-interventional controls. The pooled effect estimate and pooled 95% Confidence Interval (CI) are computed based on a Random Effects Model and shown as a diamond, in which the diamond’s width represents the range of the 95% CI. The I^2^ statistic quantifies the proportion of total variation in results across studies investigating the same dietary intervention that is due to heterogeneity rather than chance. An I^2^ value of 0% indicates no observed heterogeneity; the group of studies examining this dietary intervention are relatively homogeneous. Larger I^2^ values indicate greater heterogeneity. SMD: standardised means difference. CI: confidence interval. χ^2^: chi-square. df: degrees of freedom. p: chi-square test *p*-value. I^2^: heterogeneity statistic.

Additional file 28: Forest plot summarising the effect sizes, quantified as Standardised Means Difference (SMD), for studies assessing the impact of dietary interventions on the **fatigue resistance of the skin (i.e., R-parameter R9 on the Cutometer) compared to non-interventional controls**. Each circle represents a study's effect size. The size of each circle is proportional to its weight in the meta-analysis. Horizontal lines denote 95% confidence intervals. SMD for each study was calculated using Cohen’s d for paired samples (i.e., before vs after dietary intervention). The vertical dotted line indicates the line of no effect (SMD=0). A positive SMD indicates that the dietary intervention favours a greater fatigue resistance (i.e., R-parameter R9 on the Cutometer) when compared to non-interventional controls. The pooled effect estimate and pooled 95% Confidence Interval (CI) are computed based on a Random Effects Model and shown as a diamond, in which the diamond’s width represents the range of the 95% CI. The I^2^ statistic quantifies the proportion of total variation in results across studies investigating the same dietary intervention that is due to heterogeneity rather than chance. An I^2^ value of 0% indicates no observed heterogeneity; the group of studies examining this dietary intervention are relatively homogeneous. Larger I^2^ values indicate greater heterogeneity. SMD: standardised means difference. CI: confidence interval. χ^2^: chi-square. df: degrees of freedom. p: chi-square test *p*-value. I^2^: heterogeneity statistic.

## Data Availability

All data generated or analysed during this study are included in this published article and its supplementary information files. The datasets used and/or analysed during the current study are available from the corresponding author (F. T. C.) on reasonable request.

## Abbreviations

CI: Confidence interval
COX-2: Cyclooxygenase-2
df: Degrees of freedom
I^2^: Inconsistency index
Keap1: Kelch-like ECH-associated protein 1
MAPK: Mitogen-activated protein kinase
MMP: Matrix metalloproteinase
MMP-1: Matrix metalloproteinase 1
Nrf2: Nuclear factor erythroid 2–related factor 2
Nrf2-ARE: Nuclear factor erythroid 2–related factor 2 antioxidant response element
NF-κB: Nuclear factor kappa-light-chain-enhancer of activated B cells
PRISMA: Preferred Reporting Item for Systematic Review and Meta-Analyses
pSMD: Pooled standardised mean difference
SIRT-1: Sirtuin 1
SE: Standard error
SMD: Standardised mean difference
TEWL: Trans-epidermal water loss
