# Supplementary figures and images for: Dietary interventions in skin ageing: a systematic review and meta-analysis
Source: J Physiol Anthropol. 2025 Oct 31;44:26. doi: 10.1186/s40101-025-00408-4 (PMC12577306; doi:10.1186/s40101-025-00408-4)

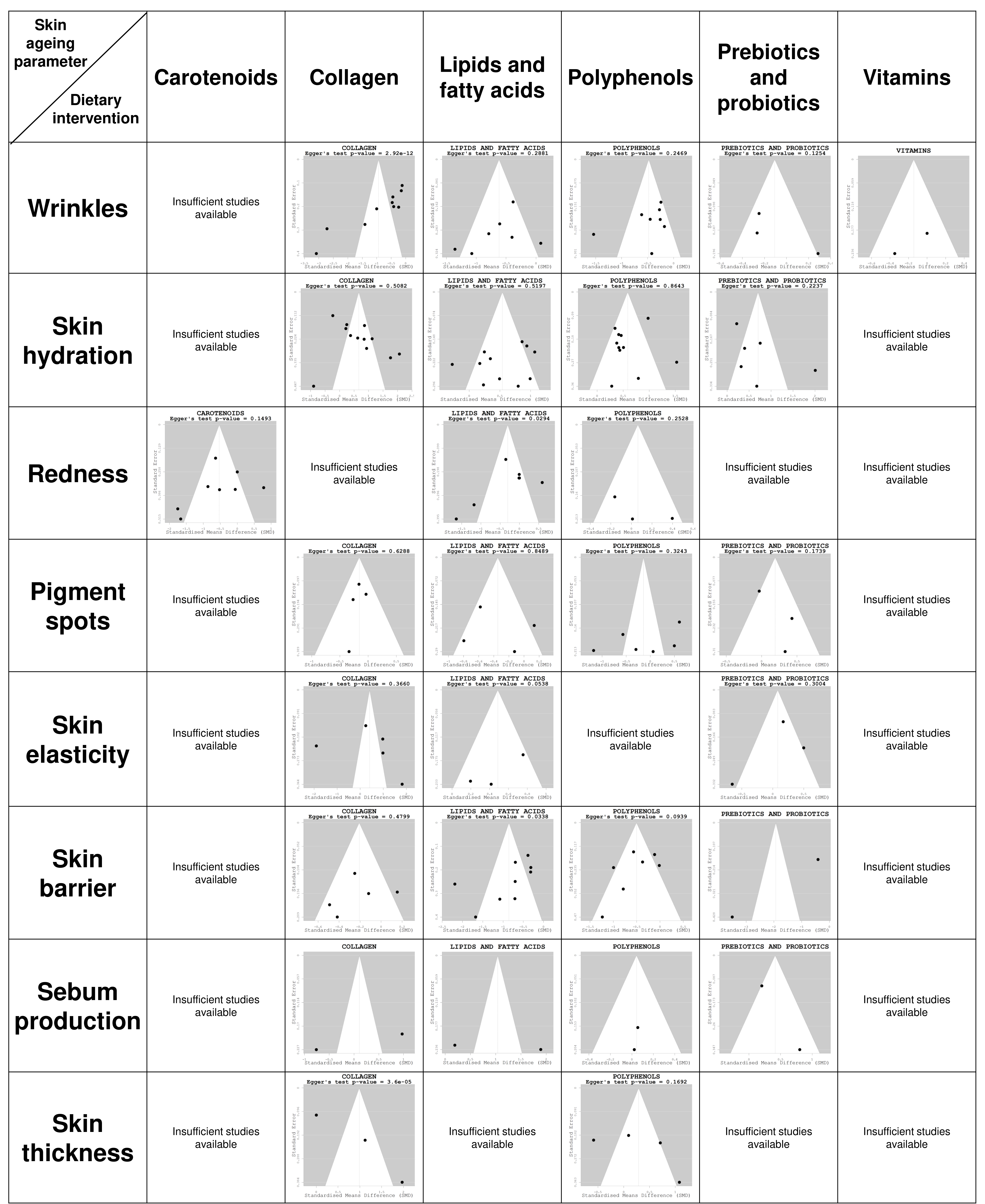

Supplement: Supplementary file 5 — Additional file 5: Begg’s funnel plots and Egger’s test p-values for detecting publication bias following dietary intervention. Funnel plots show Standardised Mean Differences (SMD) plotted against standard errors (SE) for dietary interventions with two or more studies. Funnel plots could not be drawn for interventions with fewer than two studies and are labelled as ‘Insufficient studies available’. Studies assess changes in wrinkles, hydration, redness, pigment spots, elasticity, the skin barrier, sebum production, and skin thickness following dietary intervention. The dietary groups are carotenoids, collagen, lipids and fatty acids, polyphenols, prebiotics and probiotics, and vitamins. Each dot represents a study. The vertical black dotted line represents the pooled effects size measured as pooled Odds Ratios while the diagonal black dotted lines represent the pooled 95% Confidence Interval. Studies with smaller standard errors appear higher in each funnel plot while studies with larger standard errors are positioned lower. Symmetry around the pooled effect estimate suggests the absence of publication bias, while asymmetry may indicate potential publication bias. Funnel plot symmetry is statistically quantified using Egger’s test, where two-tailed p-values lower than 0.05 suggest potential publication bias. Egger’s test p-values could only be calculated when interventions have three or more studies. SMD: standardised means difference. SE: standard error. [file 40101_2025_408_MOESM5_ESM.png]

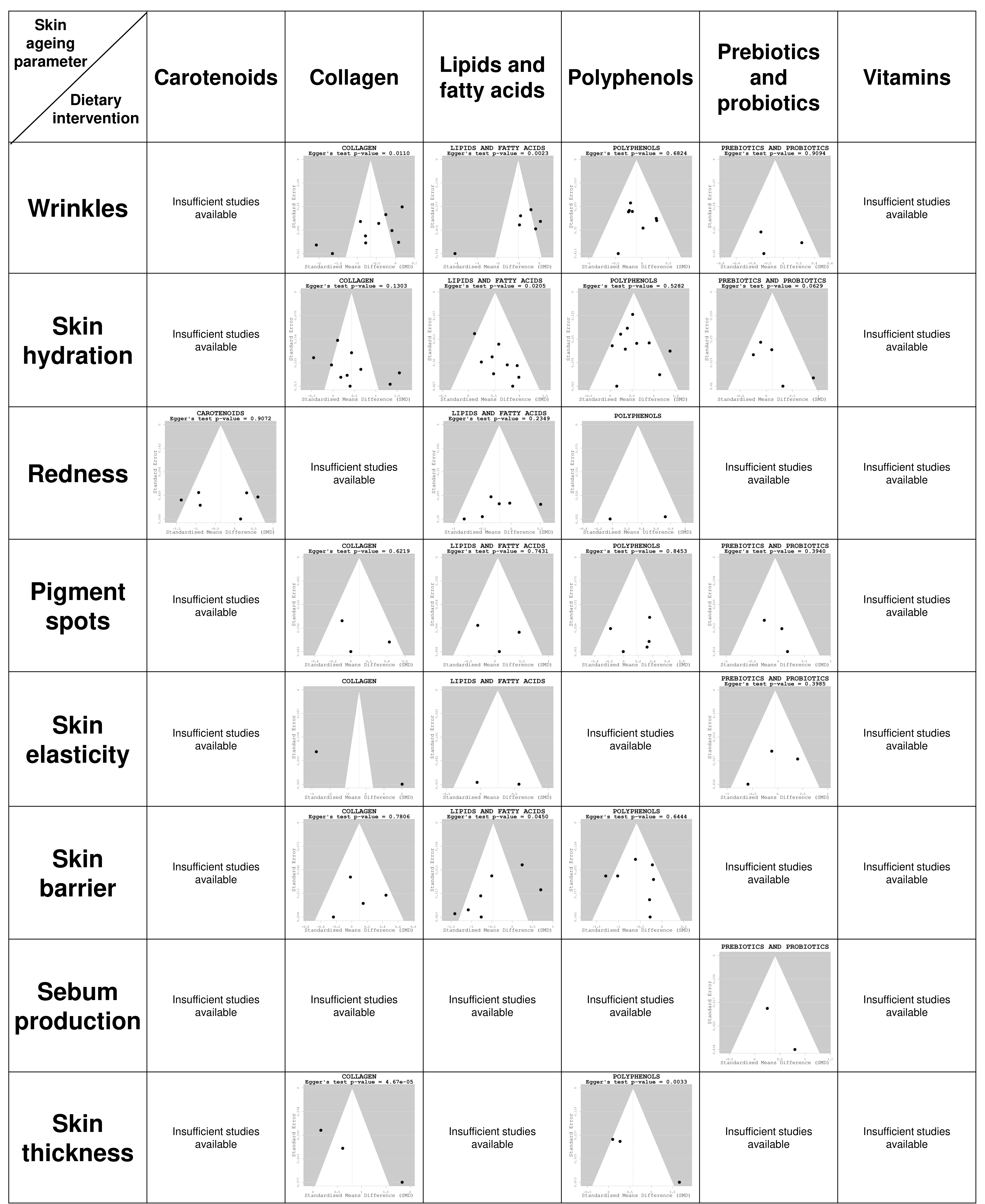

Supplement: Supplementary file 6 — Additional file 6: Begg’s funnel plots and Egger’s test p-values for detecting publication bias between dietary interventions and non-interventional controls. Funnel plots show Standardised Mean Differences (SMD) plotted against standard errors (SE) for dietary interventions with two or more studies. Funnel plots could not be drawn for interventions with fewer than two studies and are labelled as ‘Insufficient studies available’. Studies assess changes in wrinkles, hydration, redness, pigment spots, elasticity, the skin barrier, sebum production, and skin thickness between dietary interventions and non-interventional controls. The dietary groups are carotenoids, collagen, lipids and fatty acids, polyphenols, prebiotics and probiotics, and vitamins. Each dot represents a study. The vertical black dotted line represents the pooled effects size measured as pooled Odds Ratios while the diagonal black dotted lines represent the pooled 95% Confidence Interval. Studies with smaller standard errors appear higher in each funnel plot while studies with larger standard errors are positioned lower. Symmetry around the pooled effect estimate suggests the absence of publication bias, while asymmetry may indicate potential publication bias. Funnel plot symmetry is statistically quantified using Egger’s test, where two-tailedp-values lower than 0.05 suggest potential publication bias. Egger’s testp-values could only be calculated when interventions have three or more studies. SMD: standardised means difference. SE: standard error. [file 40101_2025_408_MOESM6_ESM.png]

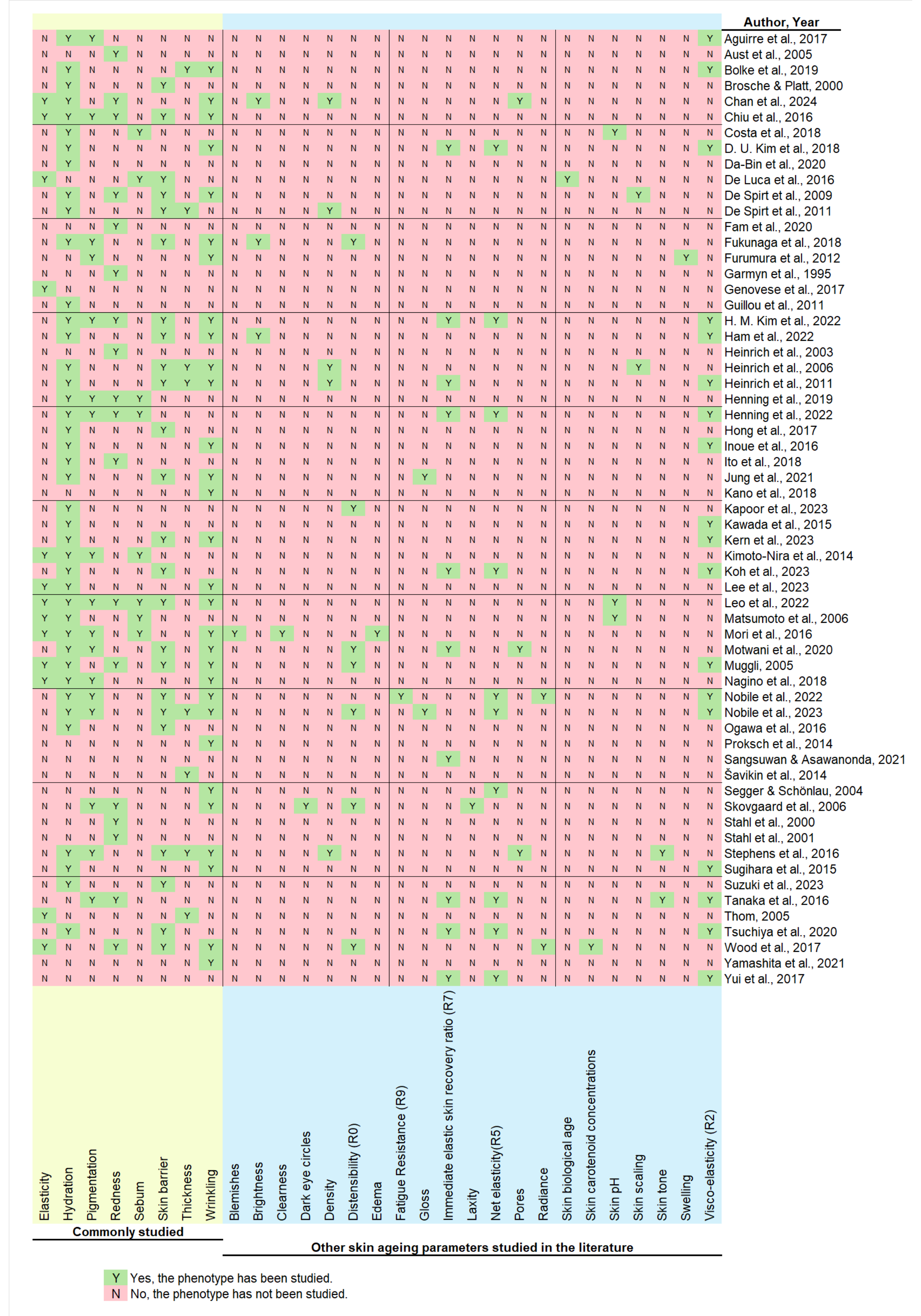

Supplement: Supplementary file 7 — Additional file 7: Diversity and scope of publications investigating dietary interventions on skin ageing. The figure summarises the range of published studies assessing different skin ageing parameters following dietary interventions, highlighting both the breadth of existing research and potential gaps in the literature. Y: Yes, the phenotype has been studied; N: No, the phenotype has not been studied. [file 40101_2025_408_MOESM7_ESM.png]

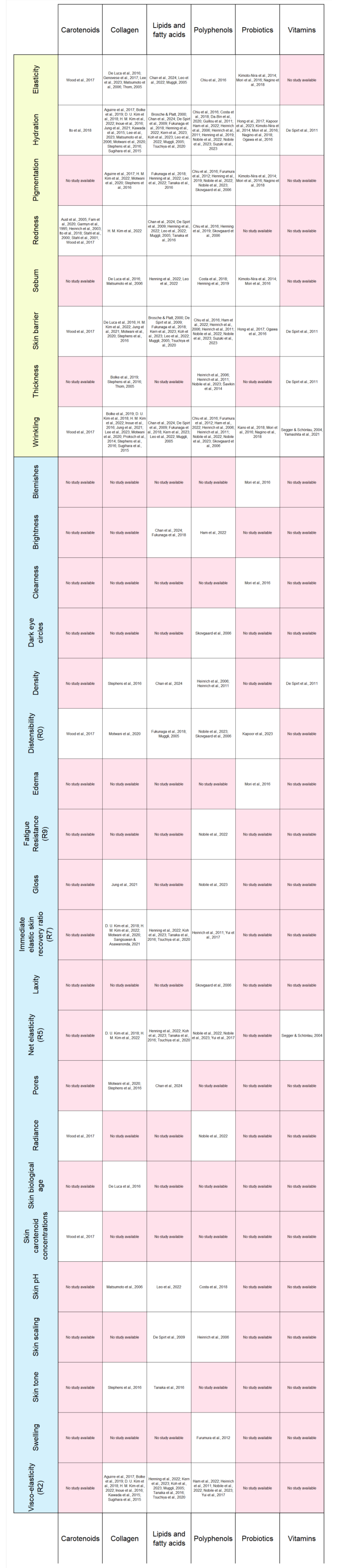

Supplement: Supplementary file 8 — Additional file 8: Diversity and scope of publications investigating dietary interventions on skin ageing, stratified by dietary interventions. Publications are stratified by skin ageing parameter (rows) and dietary intervention (columns). Each field indicates the presence or absence, and the number of studies assessing each parameter-intervention pair. Fields with no study available are shaded in pink. This figure quantifies research coverage and highlights gaps in the existing literature. [file 40101_2025_408_MOESM8_ESM.png]

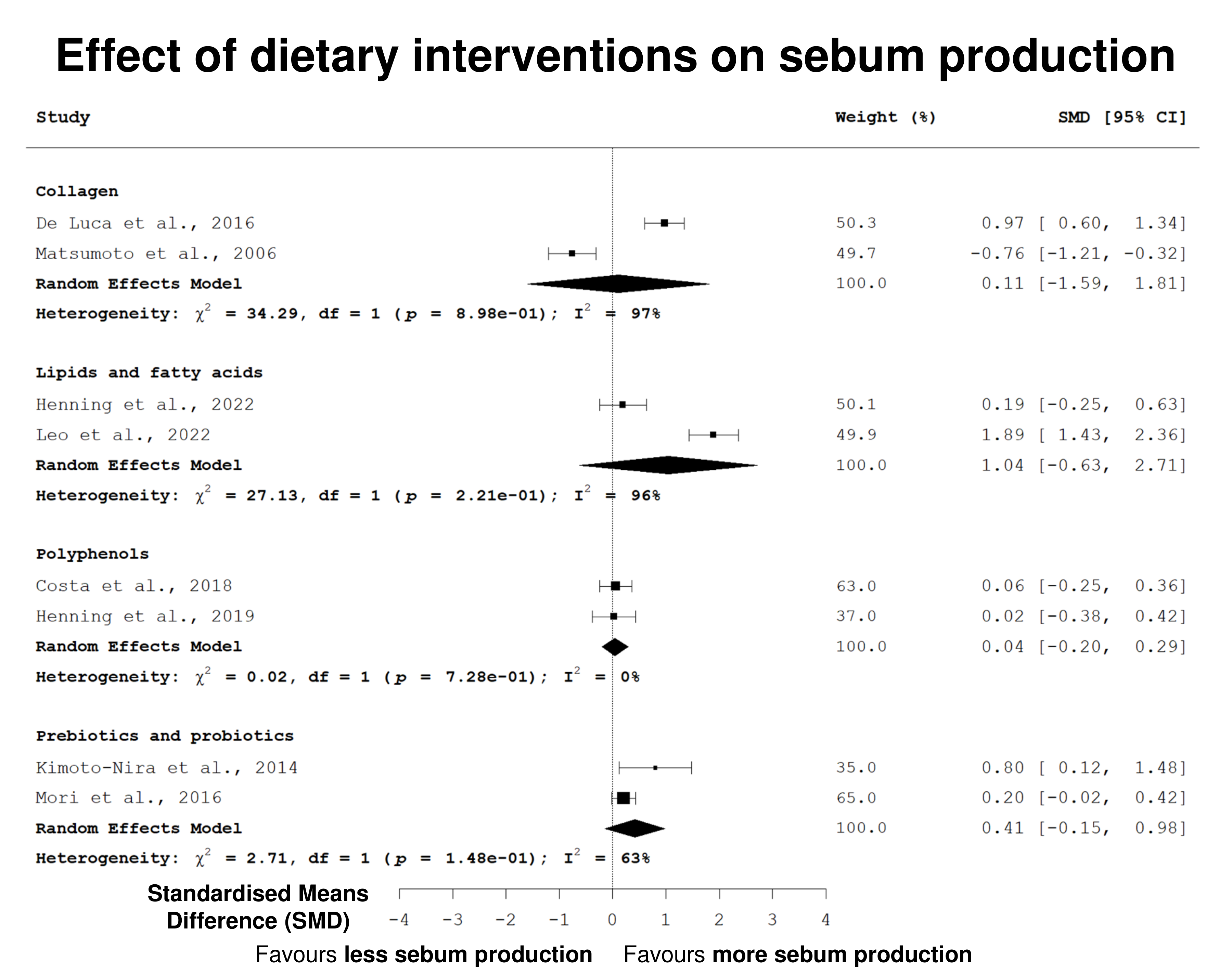

Supplement: Supplementary file 9 — Additional file 9: Forest plot summarising the effect sizes, quantified as Standardised Means Difference (SMD), for studies assessing the impact of dietary interventions on sebum production compared to before the dietary intervention. Each circle represents a study's effect size. The size of each circle is proportional to its weight in the meta-analysis. Horizontal lines denote 95% confidence intervals. SMD for each study was calculated using Cohen’s d for paired samples (i.e., before vs after dietary intervention). The vertical dotted line indicates the line of no effect (SMD=0). A positive SMD indicates that the dietary intervention favours more sedum production when compared to before the dietary intervention. The pooled effect estimate and pooled 95% Confidence Interval (CI) are computed based on a Random Effects Model and shown as a diamond, in which the diamond’s width represents the range of the 95% CI. The I² statistic quantifies the proportion of total variation in results across studies investigating the same dietary intervention that is due to heterogeneity rather than chance. An I2 value of 0% indicates no observed heterogeneity; the group of studies examining this dietary intervention are relatively homogeneous. Larger I2 values indicate greater heterogeneity. SMD: standardised means difference. CI: confidence interval. χ2: chi-square. df: degrees of freedom. p: chi-square test p-value. I2: heterogeneity statistic. [file 40101_2025_408_MOESM9_ESM.png]

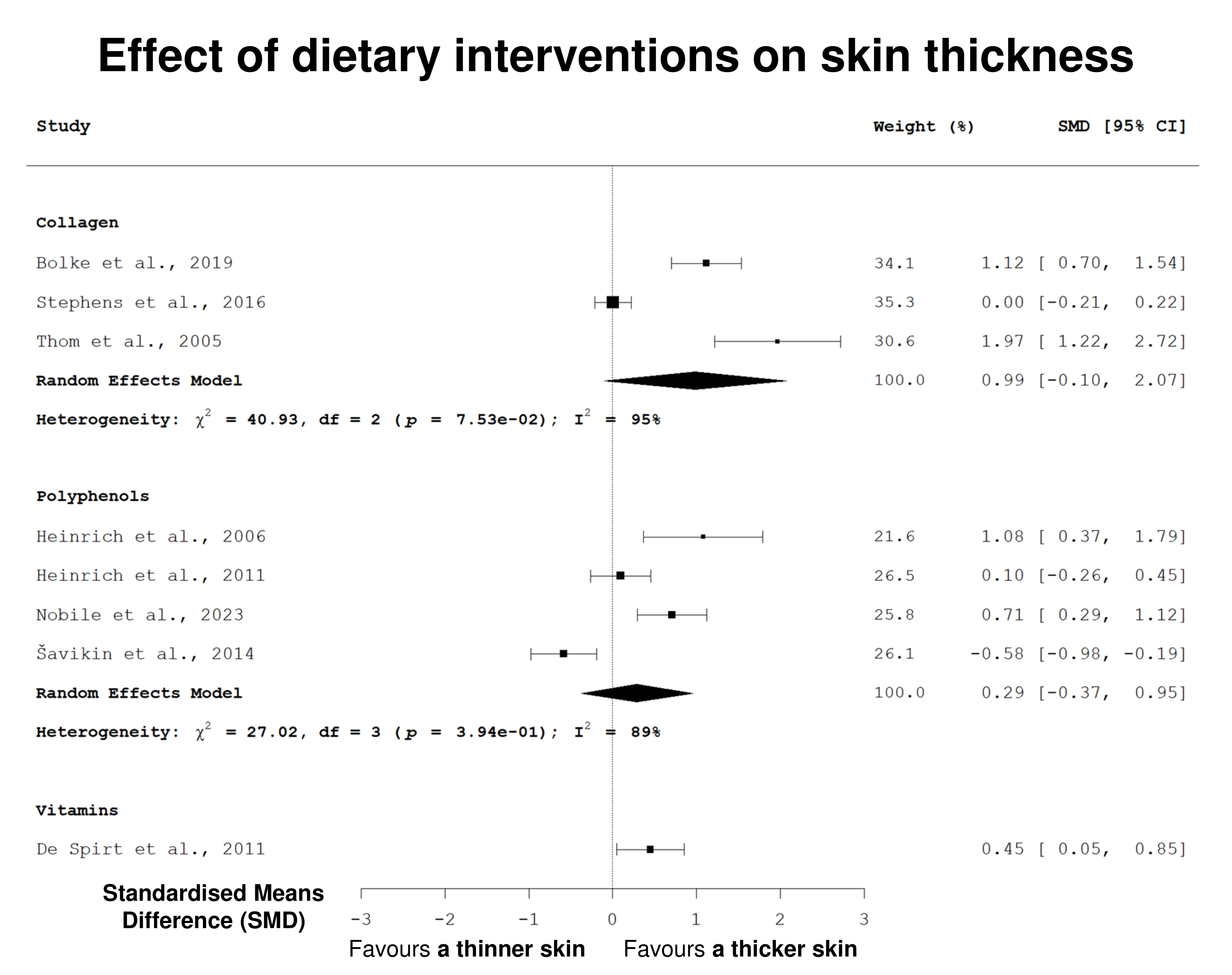

Supplement: Supplementary file 10 — Additional file 10: Forest plot summarising the effect sizes, quantified as Standardised Means Difference (SMD), for studies assessing the impact of dietary interventions on skin thickness compared to before the dietary intervention. Each circle represents a study's effect size. The size of each circle is proportional to its weight in the meta-analysis. Horizontal lines denote 95% confidence intervals. SMD for each study was calculated using Cohen’s d for paired samples (i.e., before vs after dietary intervention). The vertical dotted line indicates the line of no effect (SMD=0). A positive SMD indicates that the dietary intervention favours a greater skin thickness when compared to before the dietary intervention. The pooled effect estimate and pooled 95% Confidence Interval (CI) are computed based on a Random Effects Model and shown as a diamond, in which the diamond’s width represents the range of the 95% CI. The I2 statistic quantifies the proportion of total variation in results across studies investigating the same dietary intervention that is due to heterogeneity rather than chance. An I2 value of 0% indicates no observed heterogeneity; the group of studies examining this dietary intervention are relatively homogeneous. Larger I2 values indicate greater heterogeneity. SMD: standardised means difference. CI: confidence interval. χ2: chi-square. df: degrees of freedom. p: chi-square test p-value. I2: heterogeneity statistic. [file 40101_2025_408_MOESM10_ESM.png]

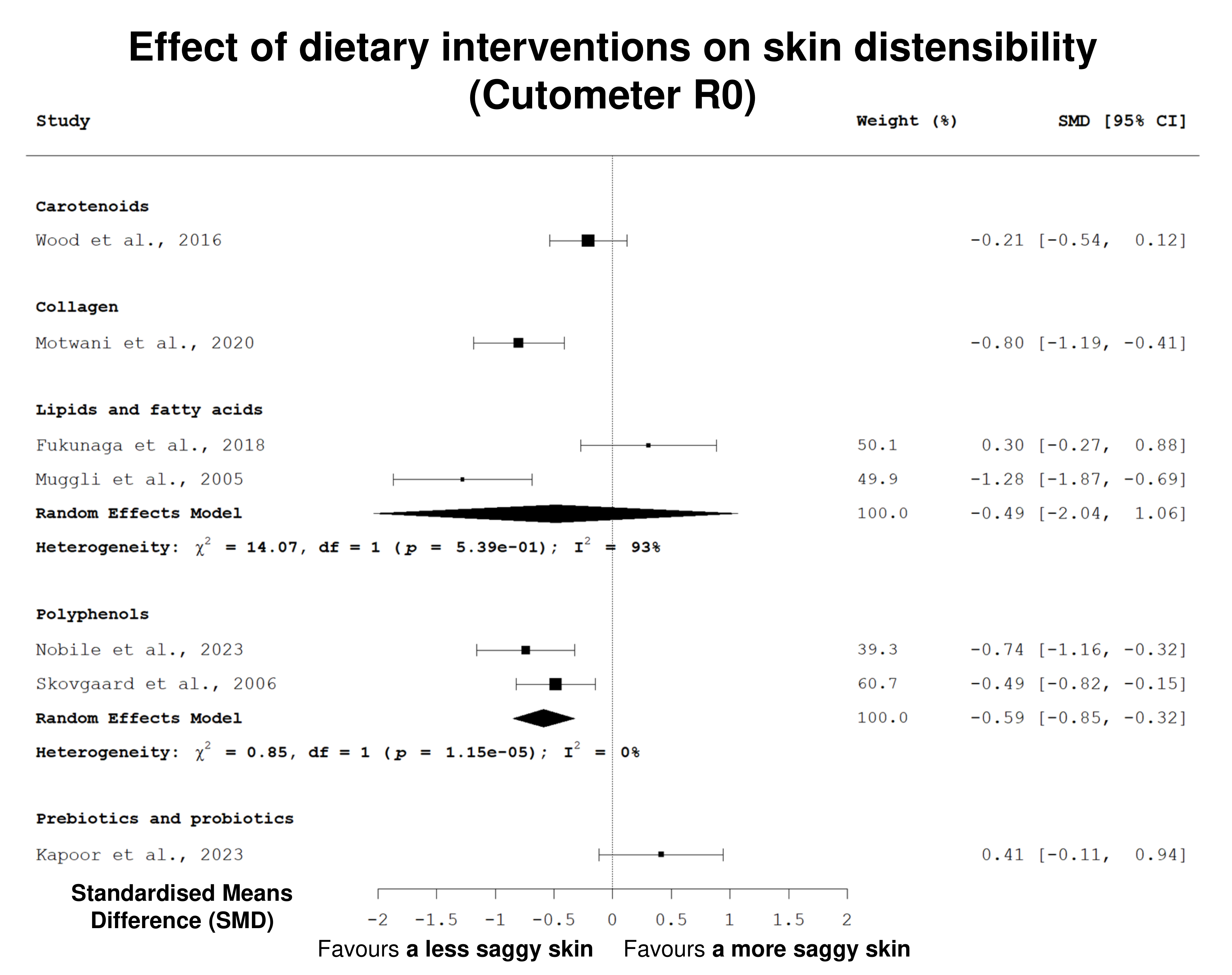

Supplement: Supplementary file 11 — Additional file 11: Forest plot summarising the effect sizes, quantified as Standardised Means Difference (SMD), for studies assessing the impact of dietary interventions on skin distensibility (i.e., R-parameter R0 on the Cutometer) compared to before the dietary intervention. Each circle represents a study's effect size. The size of each circle is proportional to its weight in the meta-analysis. Horizontal lines denote 95% confidence intervals. SMD for each study was calculated using Cohen’s d for paired samples (i.e., before vs after dietary intervention). The vertical dotted line indicates the line of no effect (SMD=0). A negative SMD indicates that the dietary intervention favours a less saggy skin (i.e., less skin distensibility, R-parameter R0 on the Cutometer) when compared to before the dietary intervention. The pooled effect estimate and pooled 95% Confidence Interval (CI) are computed based on a Random Effects Model and shown as a diamond, in which the diamond’s width represents the range of the 95% CI. The I2 statistic quantifies the proportion of total variation in results across studies investigating the same dietary intervention that is due to heterogeneity rather than chance. An I2 value of 0% indicates no observed heterogeneity; the group of studies examining this dietary intervention are relatively homogeneous. Larger I2 values indicate greater heterogeneity. SMD: standardised means difference. CI: confidence interval. χ2: chi-square. df: degrees of freedom. p: chi-square testp-value. I2: heterogeneity statistic. [file 40101_2025_408_MOESM11_ESM.png]

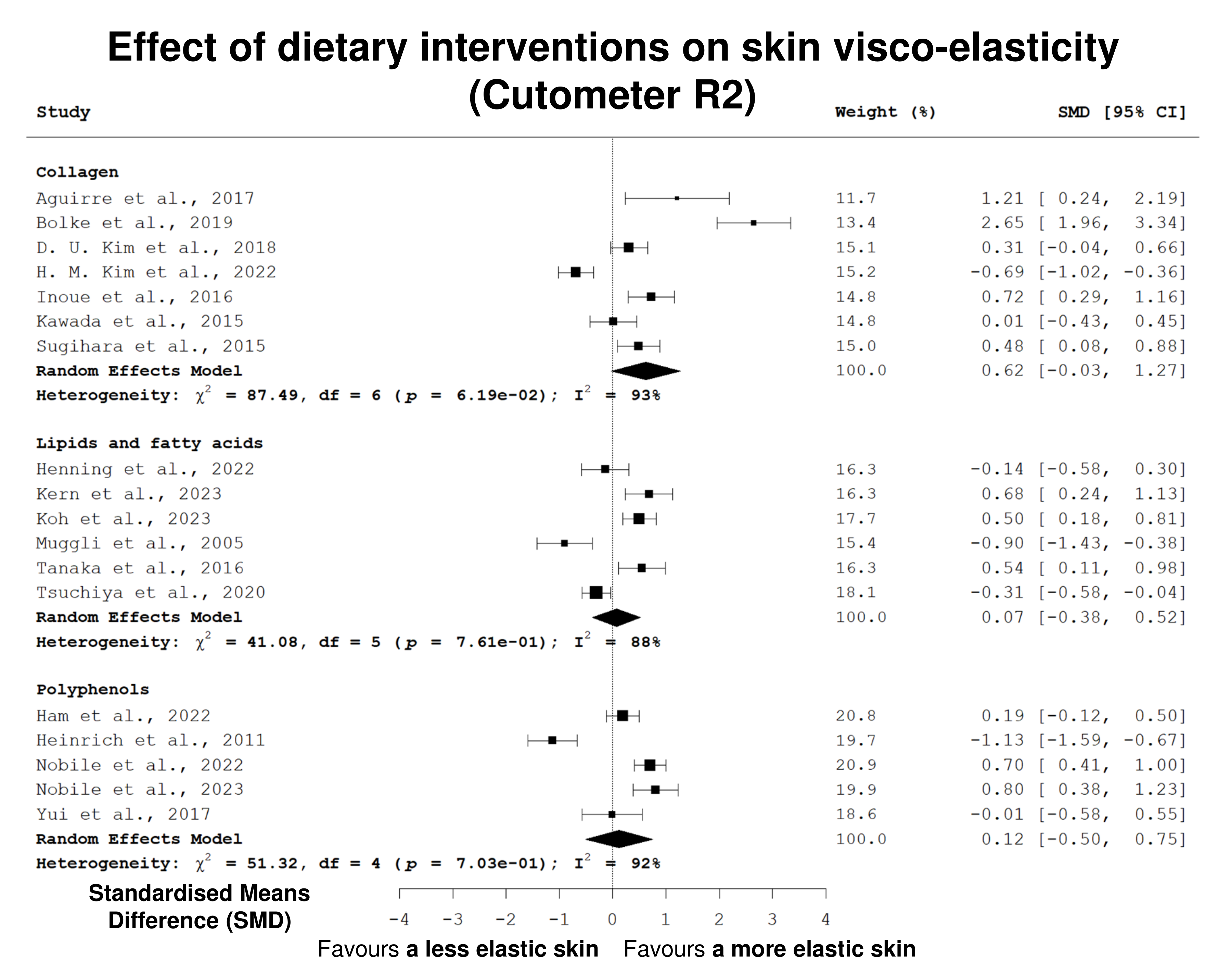

Supplement: Supplementary file 12 — Additional file 12: Forest plot summarising the effect sizes, quantified as Standardised Means Difference (SMD), for studies assessing the impact of dietary interventions on the overall visco-elasticity of the skin (i.e., R-parameter R2 on the Cutometer) compared to before the dietary intervention. Each circle represents a study's effect size. The size of each circle is proportional to its weight in the meta-analysis. Horizontal lines denote 95% confidence intervals. SMD for each study was calculated using Cohen’s d for paired samples (i.e., before vs after dietary intervention). The vertical dotted line indicates the line of no effect (SMD=0). A positive SMD indicates that the dietary intervention favours a greater overall visco-elasticity of the skin (i.e., R-parameter R2 on the Cutometer) when compared to before the dietary intervention. The pooled effect estimate and pooled 95% Confidence Interval (CI) are computed based on a Random Effects Model and shown as a diamond, in which the diamond’s width represents the range of the 95% CI. The I2 statistic quantifies the proportion of total variation in results across studies investigating the same dietary intervention that is due to heterogeneity rather than chance. An I2 value of 0% indicates no observed heterogeneity; the group of studies examining this dietary intervention are relatively homogeneous. Larger I2 values indicate greater heterogeneity. SMD: standardised means difference. CI: confidence interval. χ2: chi-square. df: degrees of freedom. p: chi-square test p-value. I2: heterogeneity statistic. [file 40101_2025_408_MOESM12_ESM.png]

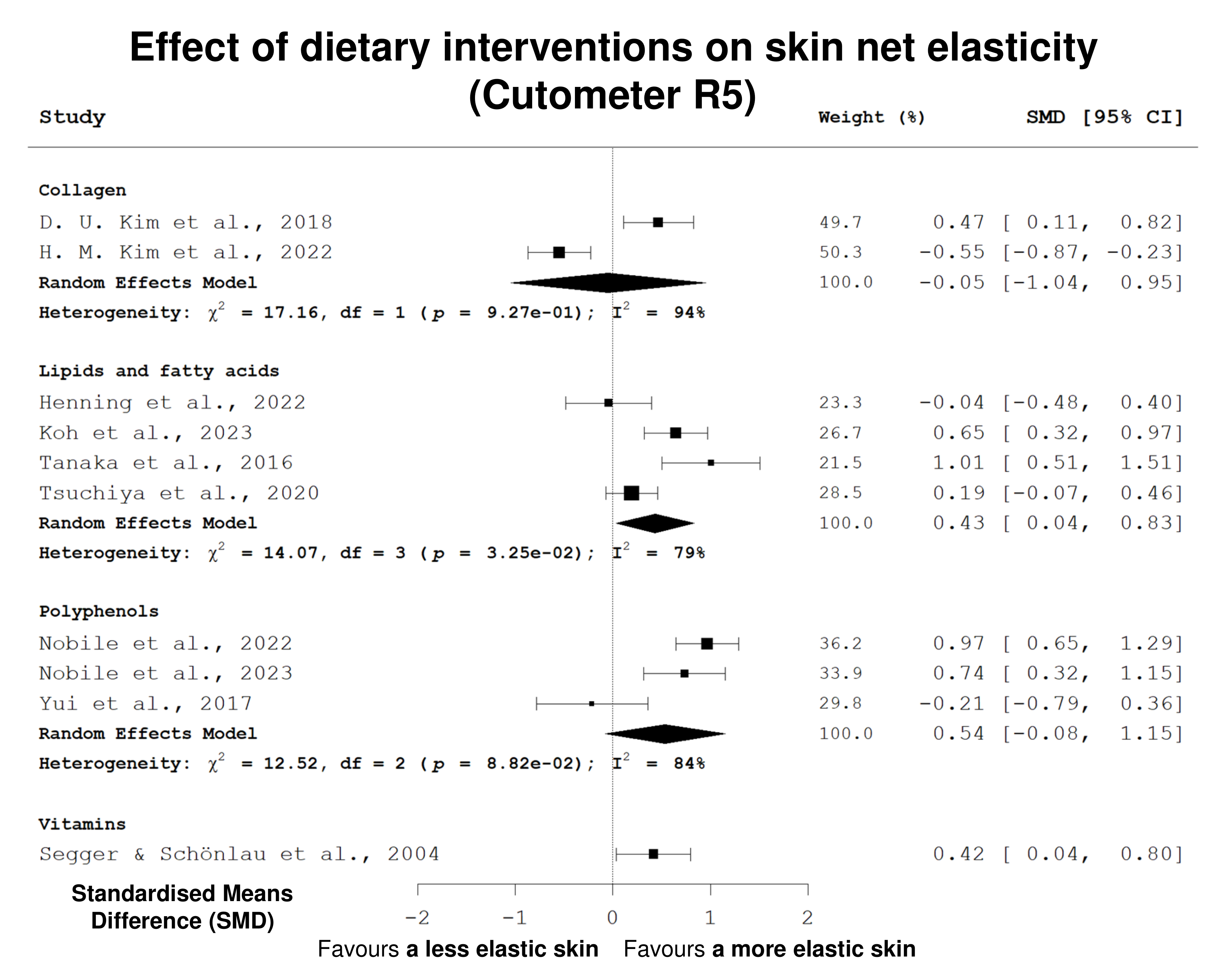

Supplement: Supplementary file 13 — Additional file 13: Forest plot summarising the effect sizes, quantified as Standardised Means Difference (SMD), for studies assessing the impact of dietary interventions on the net elasticity of the skin (i.e., R-parameter R5 on the Cutometer) compared to before the dietary intervention. Each circle represents a study's effect size. The size of each circle is proportional to its weight in the meta-analysis. Horizontal lines denote 95% confidence intervals. SMD for each study was calculated using Cohen’s d for paired samples (i.e., before vs after dietary intervention). The vertical dotted line indicates the line of no effect (SMD=0). A positive SMD indicates that the dietary intervention favours a greater net elasticity of the skin (i.e., R-parameter R5 on the Cutometer) when compared to before the dietary intervention. The pooled effect estimate and pooled 95% Confidence Interval (CI) are computed based on a Random Effects Model and shown as a diamond, in which the diamond’s width represents the range of the 95% CI. The I2 statistic quantifies the proportion of total variation in results across studies investigating the same dietary intervention that is due to heterogeneity rather than chance. An I2 value of 0% indicates no observed heterogeneity; the group of studies examining this dietary intervention are relatively homogeneous. Larger I2 values indicate greater heterogeneity. SMD: standardised means difference. CI: confidence interval. χ2: chi-square. df: degrees of freedom. p: chi-square test p-value. I2: heterogeneity statistic. [file 40101_2025_408_MOESM13_ESM.png]

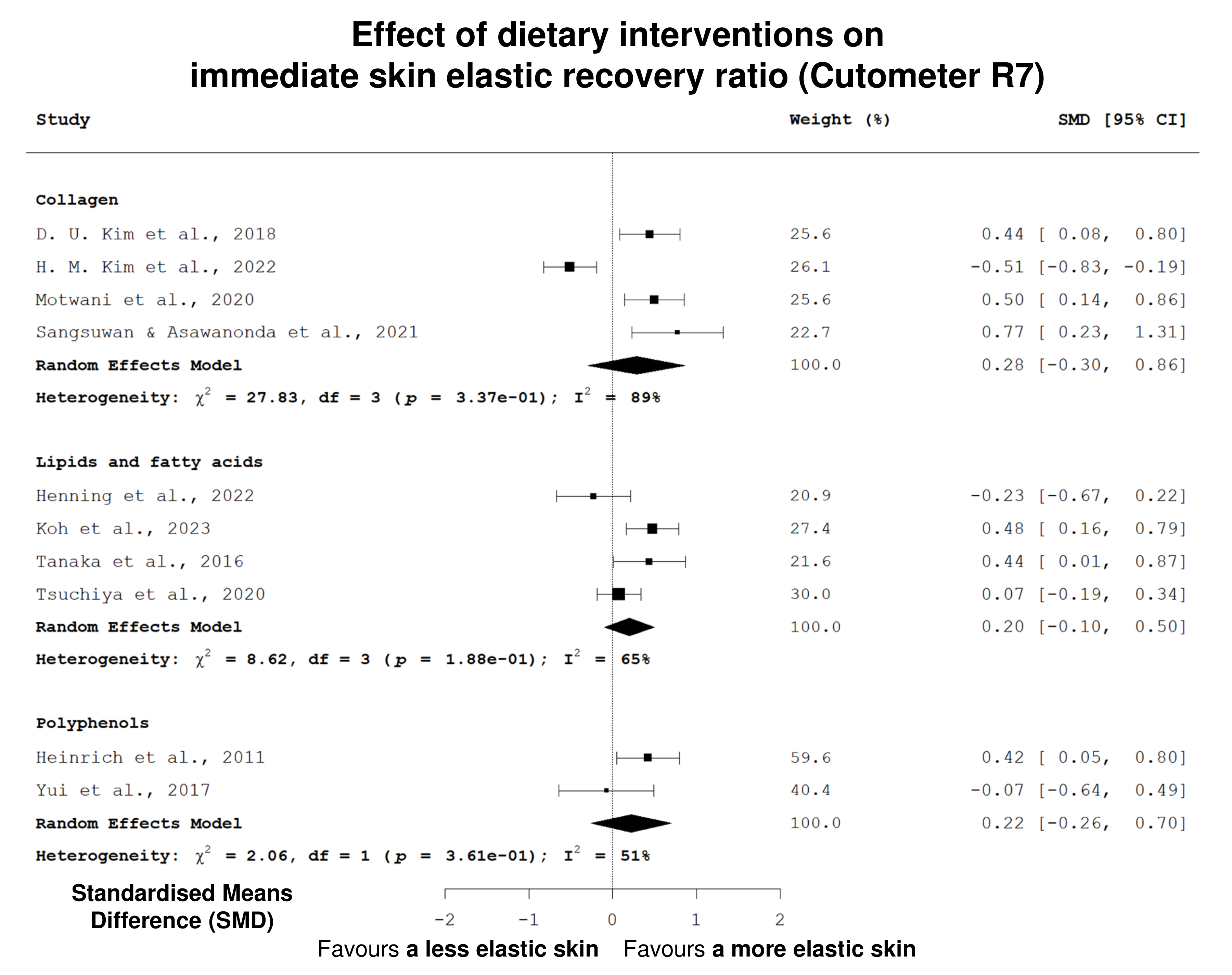

Supplement: Supplementary file 14 — Additional file 14: Forest plot summarising the effect sizes, quantified as Standardised Means Difference (SMD), for studies assessing the impact of dietary interventions on the immediate recovery of the skin after suction (i.e., R-parameter R7 on the Cutometer) compared to before the dietary intervention. Each circle represents a study's effect size. The size of each circle is proportional to its weight in the meta-analysis. Horizontal lines denote 95% confidence intervals. SMD for each study was calculated using Cohen’s d for paired samples (i.e., before vs after dietary intervention). The vertical dotted line indicates the line of no effect (SMD=0). A positive SMD indicates that the dietary intervention favours a greater immediate recovery of the skin after suction (i.e., R-parameter R7 on the Cutometer) when compared to before the dietary intervention. The pooled effect estimate and pooled 95% Confidence Interval (CI) are computed based on a Random Effects Model and shown as a diamond, in which the diamond’s width represents the range of the 95% CI. The I2 statistic quantifies the proportion of total variation in results across studies investigating the same dietary intervention that is due to heterogeneity rather than chance. An I2 value of 0% indicates no observed heterogeneity; the group of studies examining this dietary intervention are relatively homogeneous. Larger I2 values indicate greater heterogeneity. SMD: standardised means difference. SMD: standardised means difference. CI: confidence interval. χ2: chi-square. df: degrees of freedom. p: chi-square test p-value. I2: heterogeneity statistic. [file 40101_2025_408_MOESM14_ESM.png]

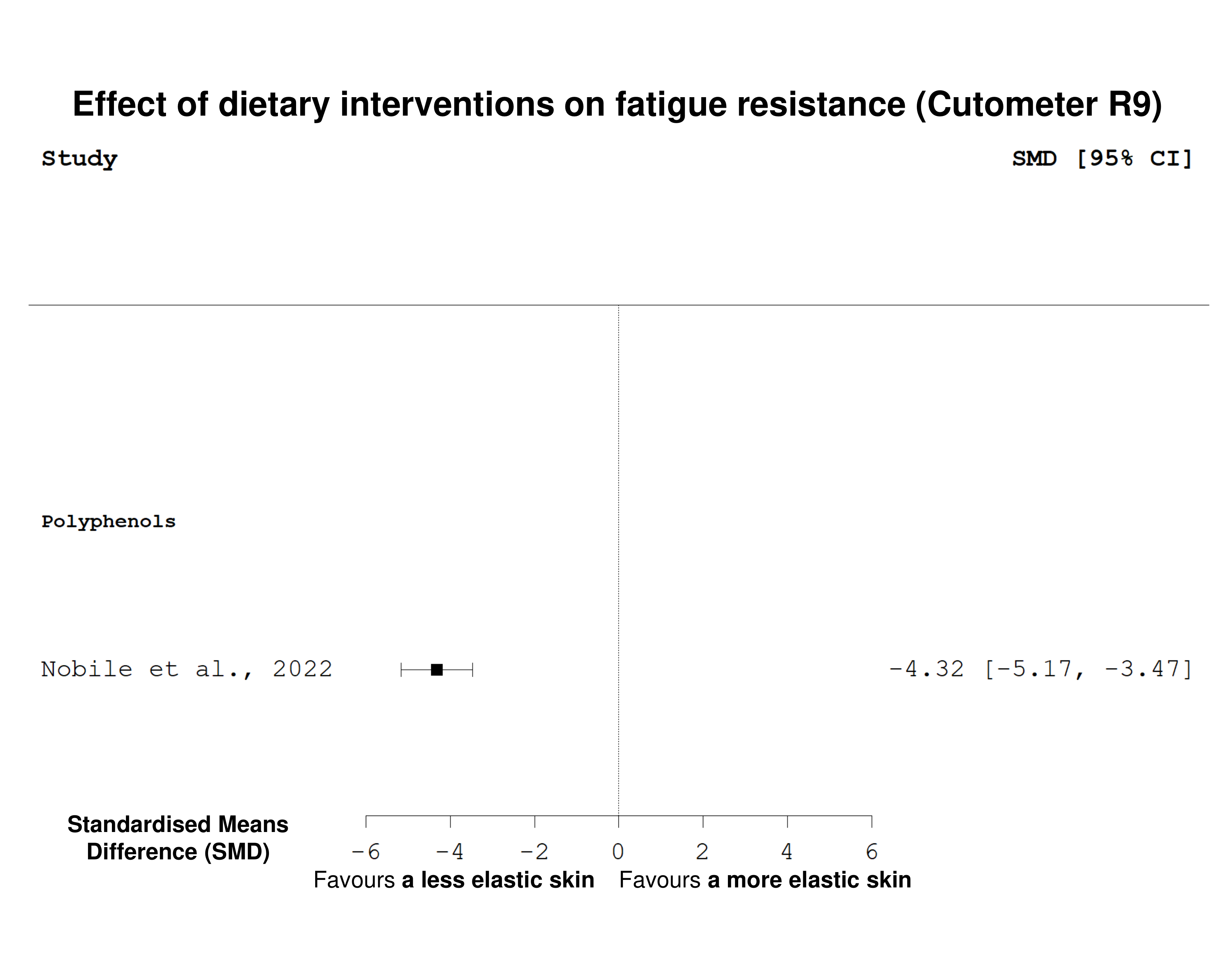

Supplement: Supplementary file 15 — Additional file 15: Forest plot summarising the effect sizes, quantified as Standardised Means Difference (SMD), for studies assessing the impact of dietary interventions on the fatigue resistance of the skin (i.e., R-parameter R9 on the Cutometer) compared to before the dietary intervention. Each circle represents a study's effect size. The size of each circle is proportional to its weight in the meta-analysis. Horizontal lines denote 95% confidence intervals. SMD for each study was calculated using Cohen’s d for paired samples (i.e., before vs after dietary intervention). The vertical dotted line indicates the line of no effect (SMD=0). A positive SMD indicates that the dietary intervention favours a greater fatigue resistance (i.e., R-parameter R9 on the Cutometer) when compared to before the dietary intervention. The pooled effect estimate and pooled 95% Confidence Interval (CI) are computed based on a Random Effects Model and shown as a diamond, in which the diamond’s width represents the range of the 95% CI. The I2 statistic quantifies the proportion of total variation in results across studies investigating the same dietary intervention that is due to heterogeneity rather than chance. An I2 value of 0% indicates no observed heterogeneity; the group of studies examining this dietary intervention are relatively homogeneous. Larger I2 values indicate greater heterogeneity. SMD: standardised means difference. CI: confidence interval. χ2: chi-square. df: degrees of freedom. p: chi-square test p-value. I2: heterogeneity statistic. [file 40101_2025_408_MOESM15_ESM.png]

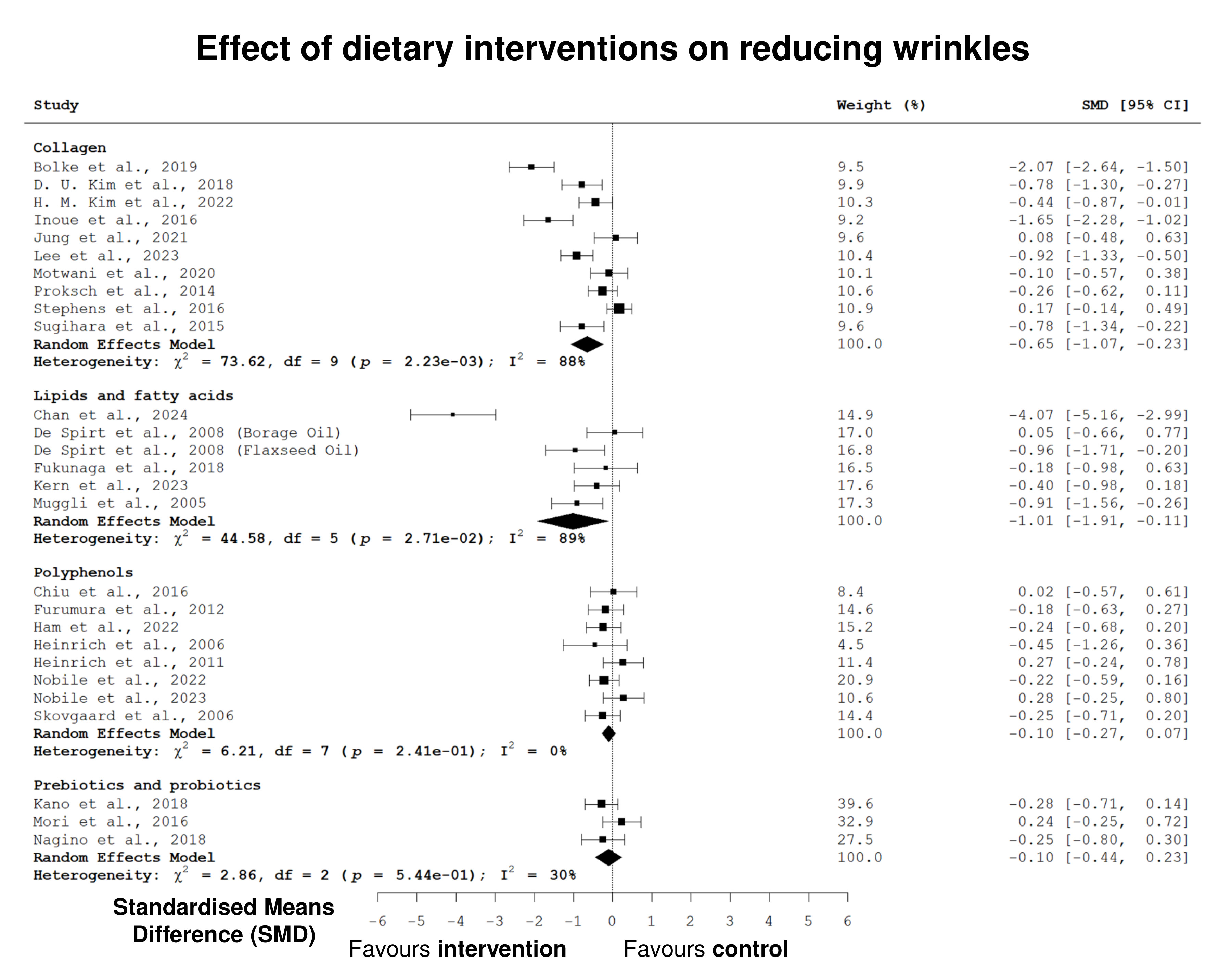

Supplement: Supplementary file 16 — Additional file 16: Forest plot summarising the effect sizes, quantified as Standardised Means Difference (SMD), for studies assessing the impact of dietary interventions on wrinkles compared to non-interventional controls. Each circle represents a study's effect size. The size of each circle is proportional to its weight in the meta-analysis. Horizontal lines denote 95% confidence intervals. SMD for each study was calculated using Cohen’s d for paired samples (i.e., before vs after dietary intervention). The vertical dotted line indicates the line of no effect (SMD=0). A negative SMD indicates that the dietary intervention favours fewer wrinkles when compared to non-interventional controls. The pooled effect estimate and pooled 95% Confidence Interval (CI) are computed based on a Random Effects Model and shown as a diamond, in which the diamond’s width represents the range of the 95% CI. The I2 statistic quantifies the proportion of total variation in results across studies investigating the same dietary intervention that is due to heterogeneity rather than chance. An I2 value of 0% indicates no observed heterogeneity; the group of studies examining this dietary intervention are relatively homogeneous. Larger I2 values indicate greater heterogeneity. SMD: standardised means difference. CI: confidence interval. χ2: chi-square. df: degrees of freedom. p: chi-square test p-value. I2: heterogeneity statistic. [file 40101_2025_408_MOESM16_ESM.png]

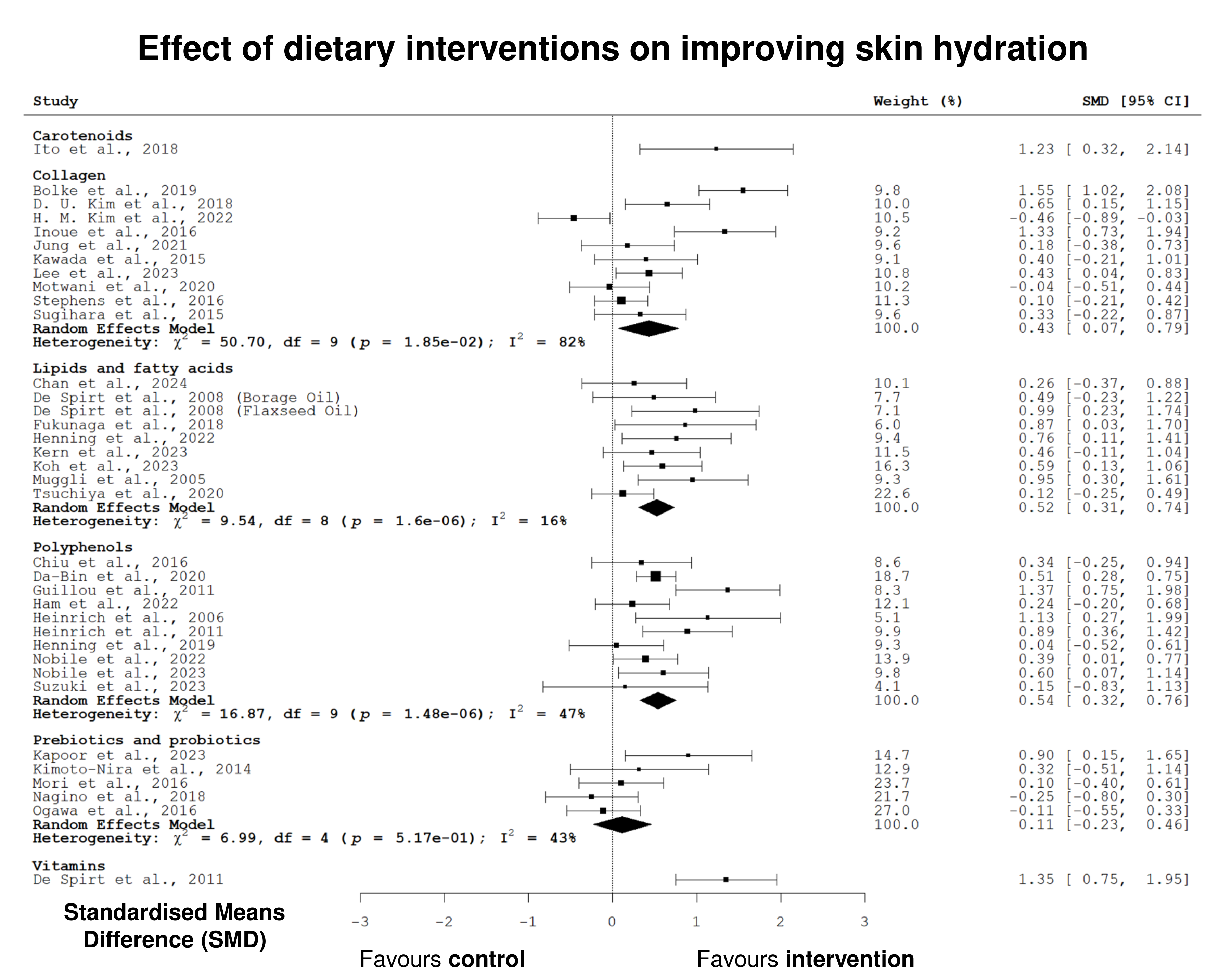

Supplement: Supplementary file 17 — Additional file 17: Forest plot summarising the effect sizes, quantified as Standardised Means Difference (SMD), for studies assessing the impact of dietary interventions on skin hydration compared to non-interventional controls. Each circle represents a study's effect size. The size of each circle is proportional to its weight in the meta-analysis. Horizontal lines denote 95% confidence intervals. SMD for each study was calculated using Cohen’s d for paired samples (i.e., before vs after dietary intervention). The vertical dotted line indicates the line of no effect (SMD=0). A positive SMD indicates that the dietary intervention favours a higher skin hydration when compared to non-interventional controls. The pooled effect estimate and pooled 95% Confidence Interval (CI) are computed based on a Random Effects Model and shown as a diamond, in which the diamond’s width represents the range of the 95% CI. The I2 statistic quantifies the proportion of total variation in results across studies investigating the same dietary intervention that is due to heterogeneity rather than chance. An I2 value of 0% indicates no observed heterogeneity; the group of studies examining this dietary intervention are relatively homogeneous. Larger I2 values indicate greater heterogeneity. SMD: standardised means difference. CI: confidence interval. χ2: chi-square. df: degrees of freedom. p: chi-square test p-value. I2: heterogeneity statistic. [file 40101_2025_408_MOESM17_ESM.png]

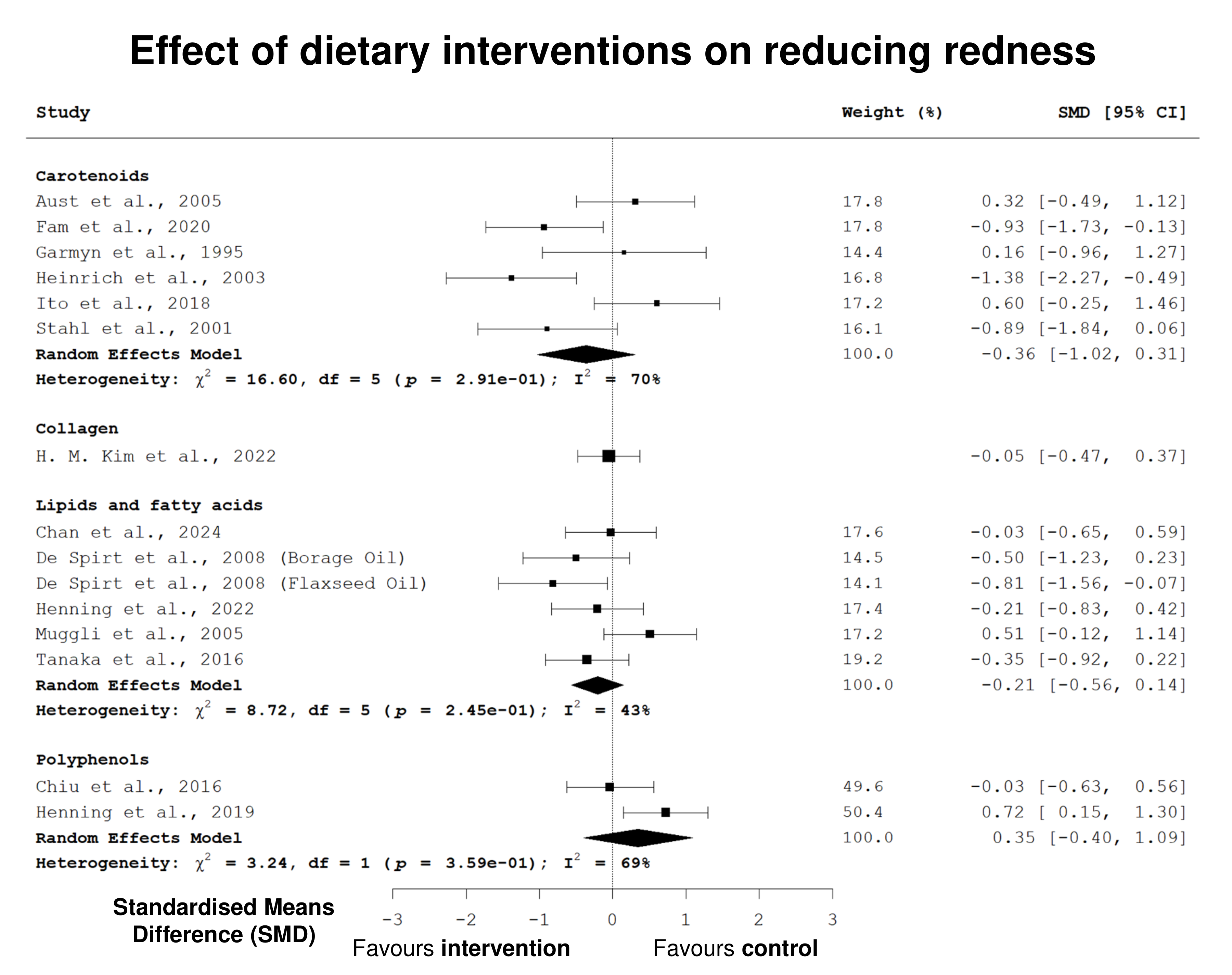

Supplement: Supplementary file 18 — Additional file 18: Forest plot summarising the effect sizes, quantified as Standardised Means Difference (SMD), for studies assessing the impact of dietary interventions on skin redness compared to non-interventional controls. Each circle represents a study's effect size. The size of each circle is proportional to its weight in the meta-analysis. Horizontal lines denote 95% confidence intervals. SMD for each study was calculated using Cohen’s d for paired samples (i.e., before vs after dietary intervention). The vertical dotted line indicates the line of no effect (SMD=0). A negative SMD indicates that the dietary intervention favours a lower skin redness when compared to non-interventional controls. The pooled effect estimate and pooled 95% Confidence Interval (CI) are computed based on a Random Effects Model and shown as a diamond, in which the diamond’s width represents the range of the 95% CI. The I2 statistic quantifies the proportion of total variation in results across studies investigating the same dietary intervention that is due to heterogeneity rather than chance. An I2 value of 0% indicates no observed heterogeneity; the group of studies examining this dietary intervention are relatively homogeneous. Larger I2 values indicate greater heterogeneity. SMD: standardised means difference. CI: confidence interval. χ2: chi-square. df: degrees of freedom. p: chi-square test p-value. I2: heterogeneity statistic. [file 40101_2025_408_MOESM18_ESM.png]

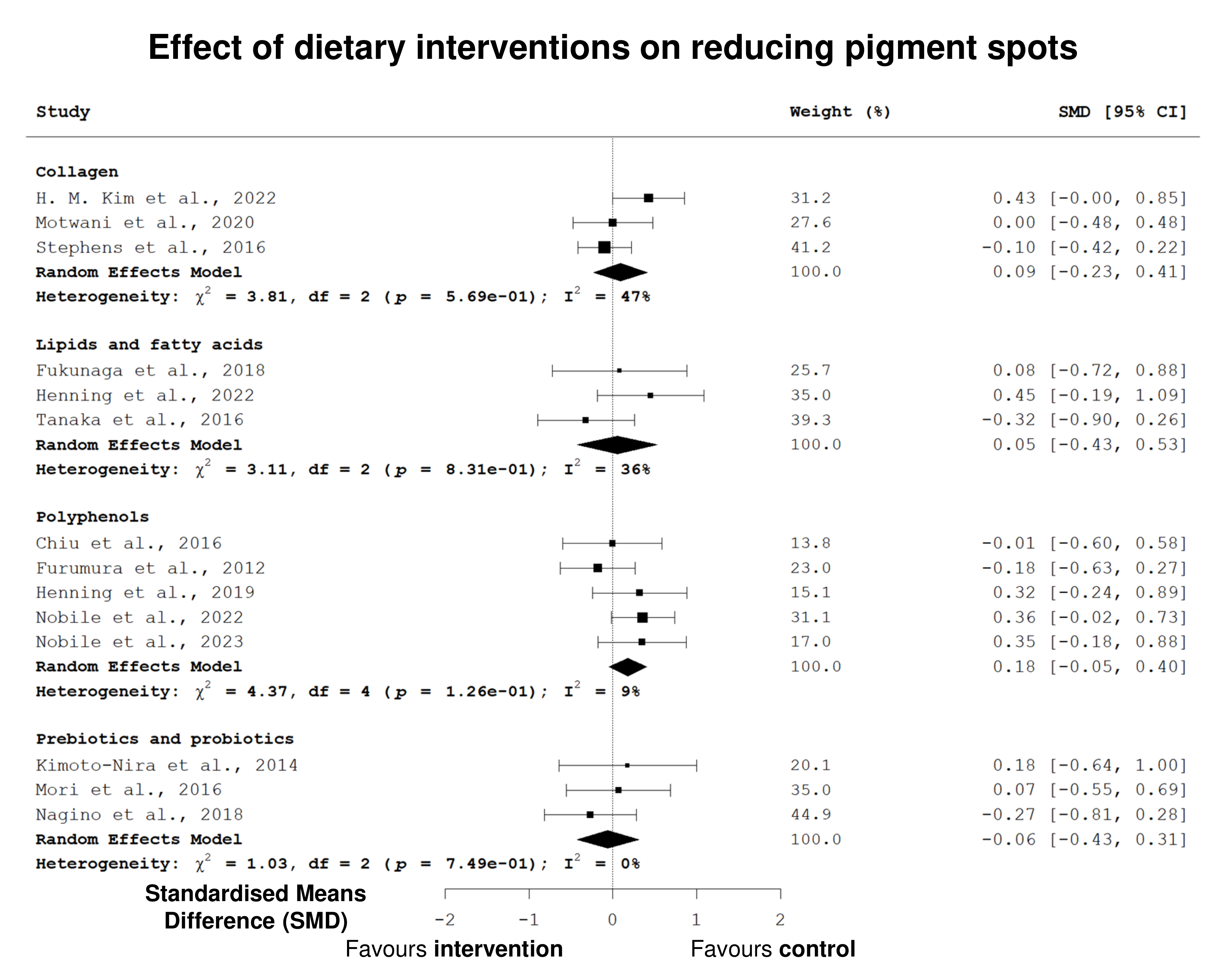

Supplement: Supplementary file 19 — Additional file 19: Forest plot summarising the effect sizes, quantified as Standardised Means Difference (SMD), for studies assessing the impact of dietary interventions on pigment spots compared to non-interventional controls. Each circle represents a study's effect size. The size of each circle is proportional to its weight in the meta-analysis. Horizontal lines denote 95% confidence intervals. SMD for each study was calculated using Cohen’s d for paired samples (i.e., before vs after dietary intervention). The vertical dotted line indicates the line of no effect (SMD=0). A negative SMD indicates that the dietary intervention favours pigment spots when compared to non-interventional controls. The pooled effect estimate and pooled 95% Confidence Interval (CI) are computed based on a Random Effects Model and shown as a diamond, in which the diamond’s width represents the range of the 95% CI. The I2 statistic quantifies the proportion of total variation in results across studies investigating the same dietary intervention that is due to heterogeneity rather than chance. An I2 value of 0% indicates no observed heterogeneity; the group of studies examining this dietary intervention are relatively homogeneous. Larger I2 values indicate greater heterogeneity. SMD: standardised means difference. CI: confidence interval. χ2: chi-square. df: degrees of freedom. p: chi-square test p-value. I2: heterogeneity statistic. [file 40101_2025_408_MOESM19_ESM.png]

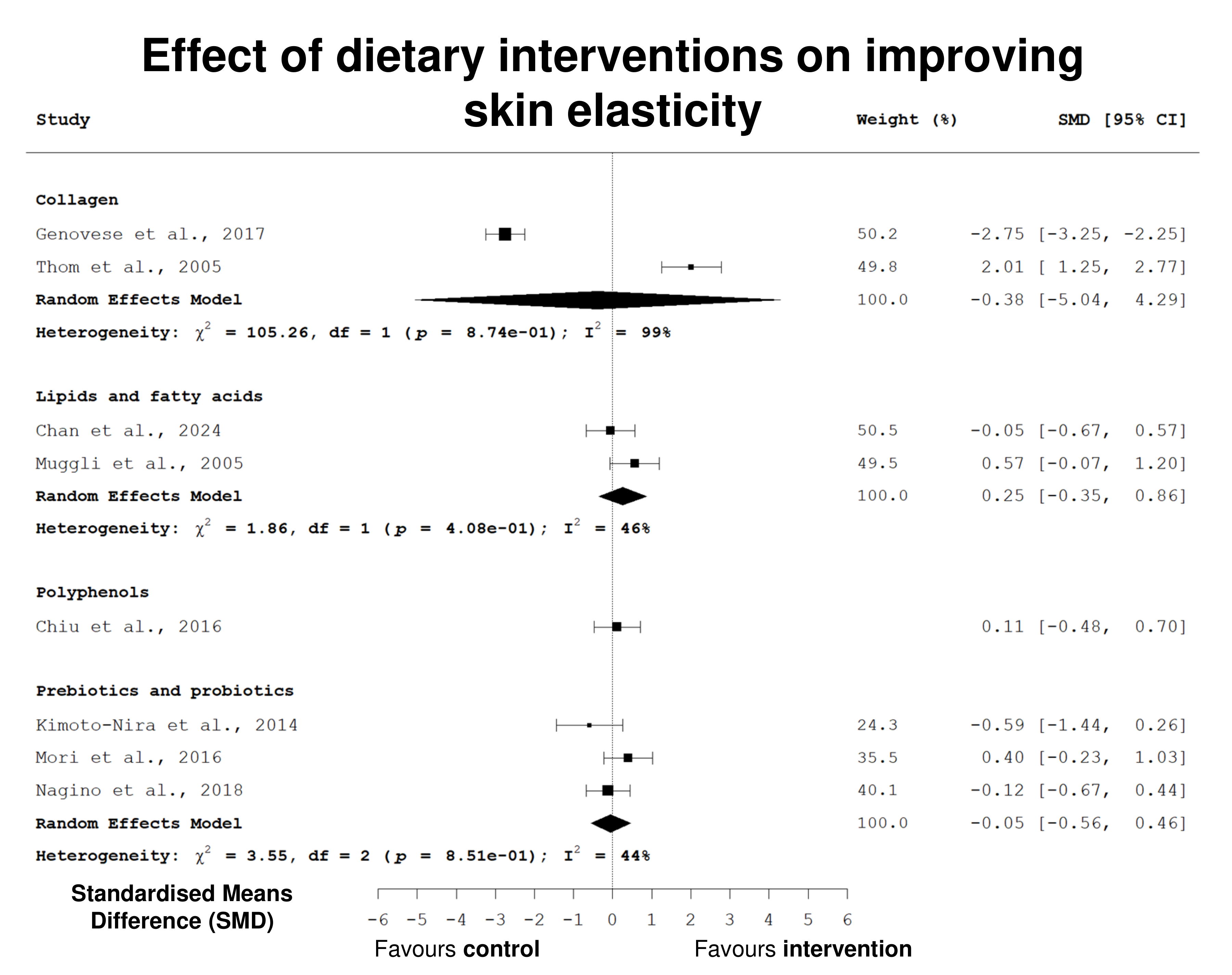

Supplement: Supplementary file 20 — Additional file 20: Forest plot summarising the effect sizes, quantified as Standardised Means Difference (SMD), for studies assessing the impact of dietary interventions on skin elasticity compared to non-interventional controls. Each circle represents a study's effect size. The size of each circle is proportional to its weight in the meta-analysis. Horizontal lines denote 95% confidence intervals. SMD for each study was calculated using Cohen’s d for paired samples (i.e., before vs after dietary intervention). The vertical dotted line indicates the line of no effect (SMD=0). A positive SMD indicates that the dietary intervention favours greater skin elasticity when compared to non-interventional controls. The pooled effect estimate and pooled 95% Confidence Interval (CI) are computed based on a Random Effects Model and shown as a diamond, in which the diamond’s width represents the range of the 95% CI. The I2 statistic quantifies the proportion of total variation in results across studies investigating the same dietary intervention that is due to heterogeneity rather than chance. An I2 value of 0% indicates no observed heterogeneity; the group of studies examining this dietary intervention are relatively homogeneous. Larger I2 values indicate greater heterogeneity. SMD: standardised means difference. CI: confidence interval. χ2: chi-square. df: degrees of freedom. p: chi-square test p-value. I2: heterogeneity statistic. [file 40101_2025_408_MOESM20_ESM.png]

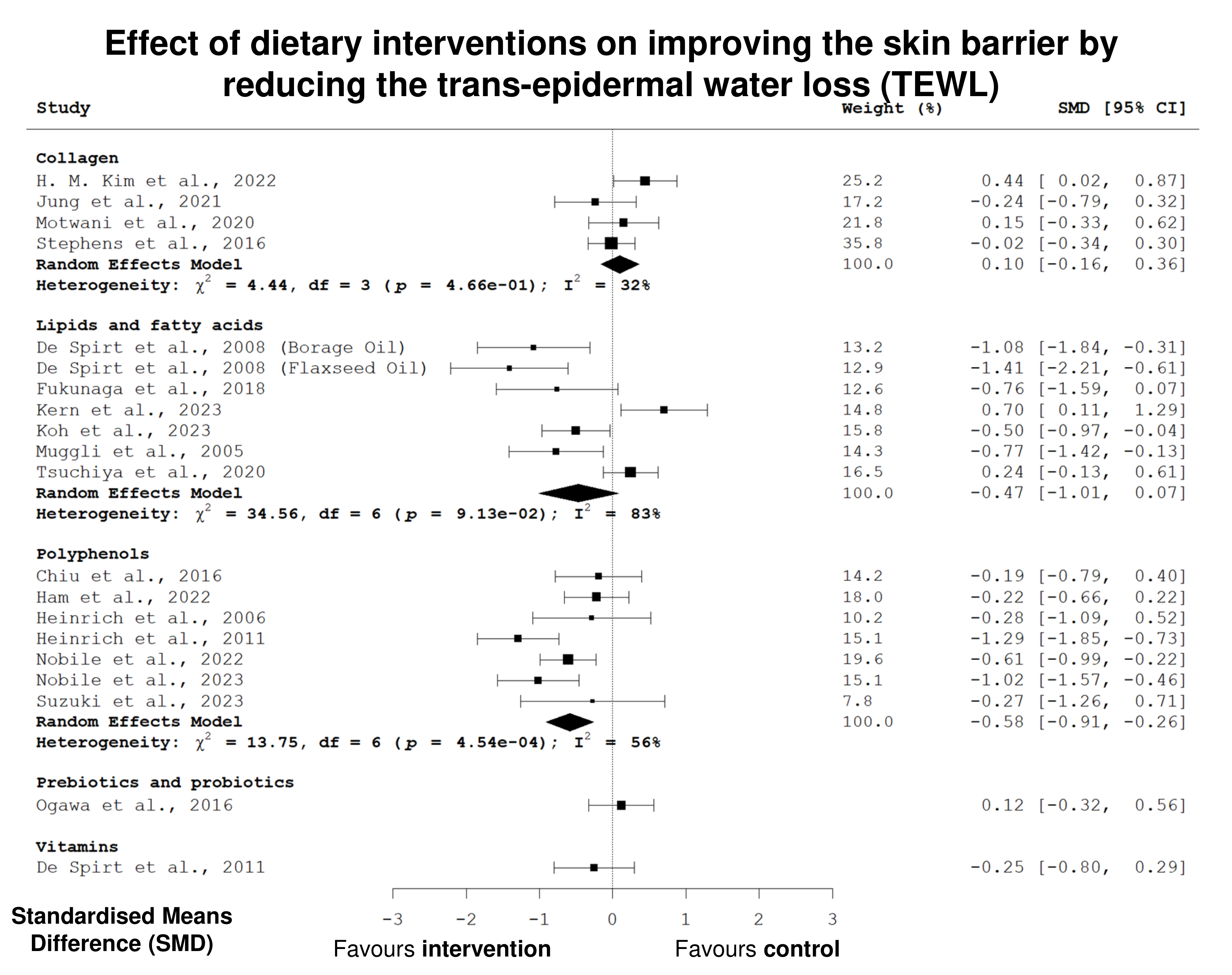

Supplement: Supplementary file 21 — Additional file 21: Forest plot summarising the effect sizes, quantified as Standardised Means Difference (SMD), for studies assessing the impact of dietary interventions on the skin barrier compared to non-interventional controls. Each circle represents a study's effect size. The size of each circle is proportional to its weight in the meta-analysis. Horizontal lines denote 95% confidence intervals. SMD for each study was calculated using Cohen’s d for paired samples (i.e., before vs after dietary intervention). The vertical dotted line indicates the line of no effect (SMD=0). A negative SMD indicates that the dietary intervention favours a lower trans-epidermal water loss (TEWL) (i.e., a better skin barrier) when compared to non-interventional controls. The pooled effect estimate and pooled 95% Confidence Interval (CI) are computed based on a Random Effects Model and shown as a diamond, in which the diamond’s width represents the range of the 95% CI. The I2 statistic quantifies the proportion of total variation in results across studies investigating the same dietary intervention that is due to heterogeneity rather than chance. An I2 value of 0% indicates no observed heterogeneity; the group of studies examining this dietary intervention are relatively homogeneous. Larger I2 values indicate greater heterogeneity. SMD: standardised means difference. CI: confidence interval. χ2: chi-square. df: degrees of freedom. p: chi-square test p-value. I2: heterogeneity statistic. TEWL: trans-epidermal water loss. [file 40101_2025_408_MOESM21_ESM.png]

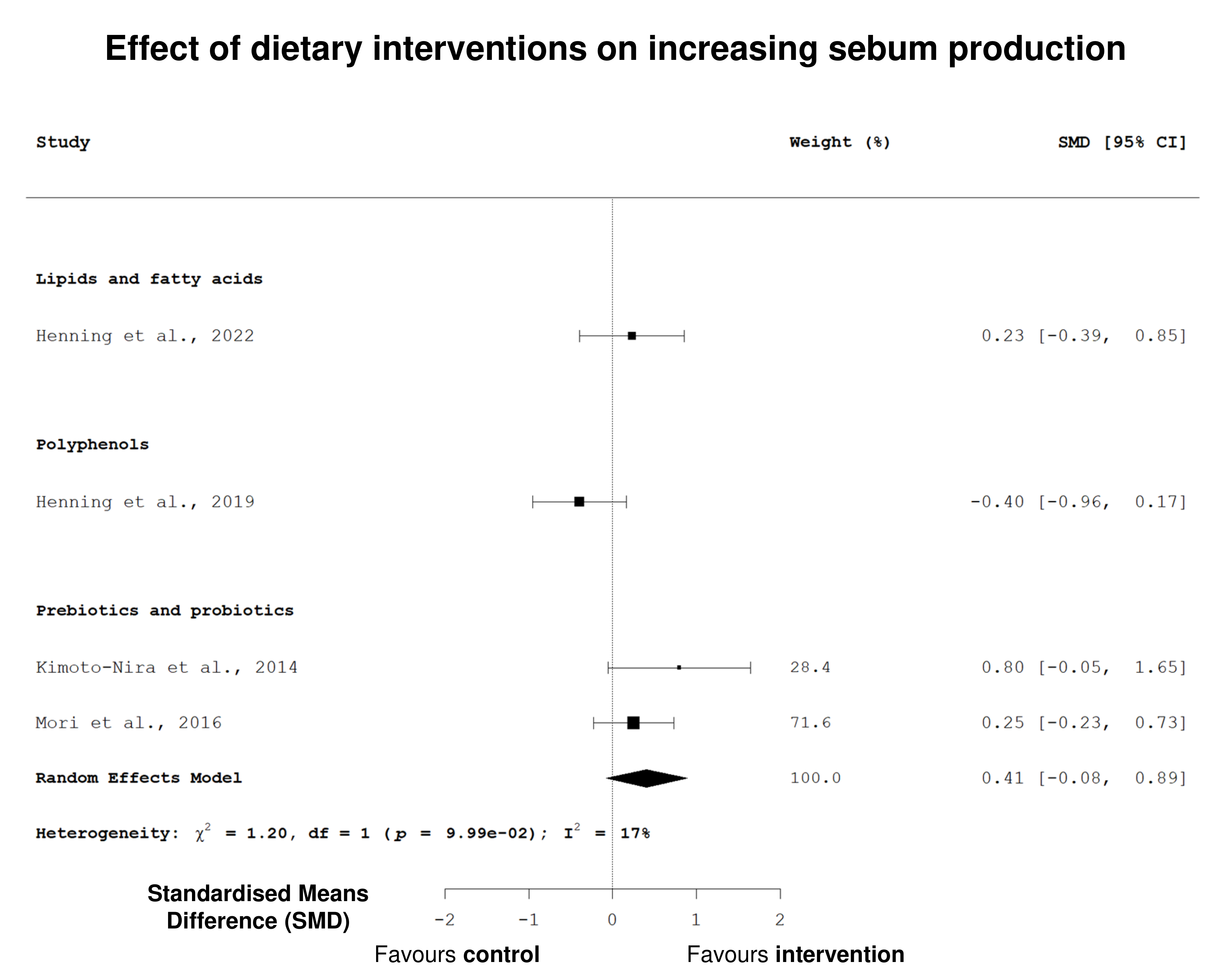

Supplement: Supplementary file 22 — Additional file 22: Forest plot summarising the effect sizes, quantified as Standardised Means Difference (SMD), for studies assessing the impact of dietary interventions on sebum production compared to non-interventional controls. Each circle represents a study's effect size. The size of each circle is proportional to its weight in the meta-analysis. Horizontal lines denote 95% confidence intervals. SMD for each study was calculated using Cohen’s d for paired samples (i.e., before vs after dietary intervention). The vertical dotted line indicates the line of no effect (SMD=0). A positive SMD indicates that the dietary intervention favours more sebum production when compared to non-interventional controls. The pooled effect estimate and pooled 95% Confidence Interval (CI) are computed based on a Random Effects Model and shown as a diamond, in which the diamond’s width represents the range of the 95% CI. The I2 statistic quantifies the proportion of total variation in results across studies investigating the same dietary intervention that is due to heterogeneity rather than chance. An I2 value of 0% indicates no observed heterogeneity; the group of studies examining this dietary intervention are relatively homogeneous. Larger I2 values indicate greater heterogeneity. SMD: standardised means difference. CI: confidence interval. χ2: chi-square. df: degrees of freedom. p: chi-square test p-value. I2: heterogeneity statistic. [file 40101_2025_408_MOESM22_ESM.png]

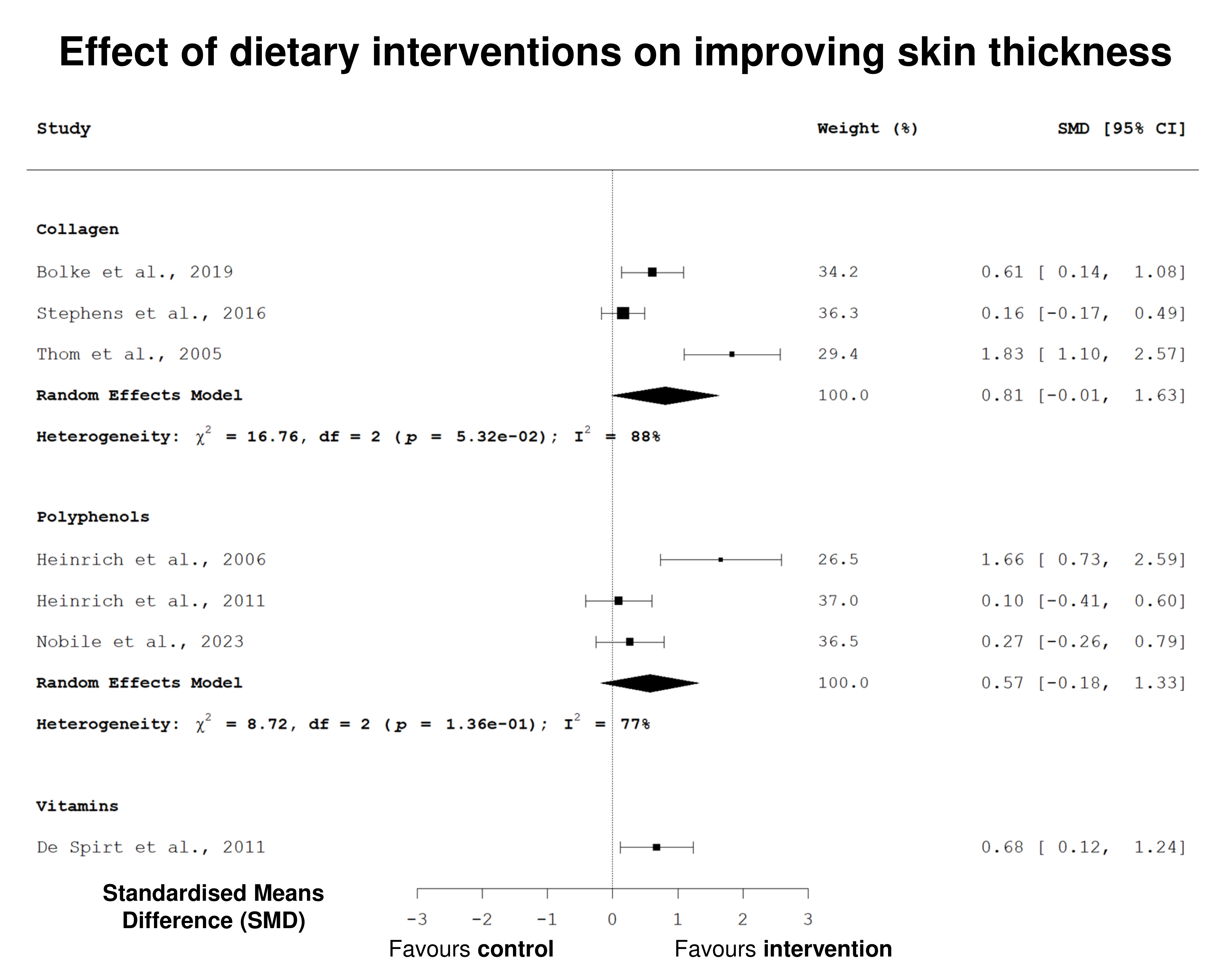

Supplement: Supplementary file 23 — Additional file 23: Forest plot summarising the effect sizes, quantified as Standardised Means Difference (SMD), for studies assessing the impact of dietary interventions on skin thickness compared to non-interventional controls. Each circle represents a study's effect size. The size of each circle is proportional to its weight in the meta-analysis. Horizontal lines denote 95% confidence intervals. SMD for each study was calculated using Cohen’s d for paired samples (i.e., before vs after dietary intervention). The vertical dotted line indicates the line of no effect (SMD=0). A positive SMD indicates that the dietary intervention favours a greater skin thickness when compared to non-interventional controls. The pooled effect estimate and pooled 95% Confidence Interval (CI) are computed based on a Random Effects Model and shown as a diamond, in which the diamond’s width represents the range of the 95% CI. The I2 statistic quantifies the proportion of total variation in results across studies investigating the same dietary intervention that is due to heterogeneity rather than chance. An I2 value of 0% indicates no observed heterogeneity; the group of studies examining this dietary intervention are relatively homogeneous. Larger I2 values indicate greater heterogeneity. SMD: standardised means difference. CI: confidence interval. χ2: chi-square. df: degrees of freedom. p: chi-square test p-value. I2: heterogeneity statistic. [file 40101_2025_408_MOESM23_ESM.png]

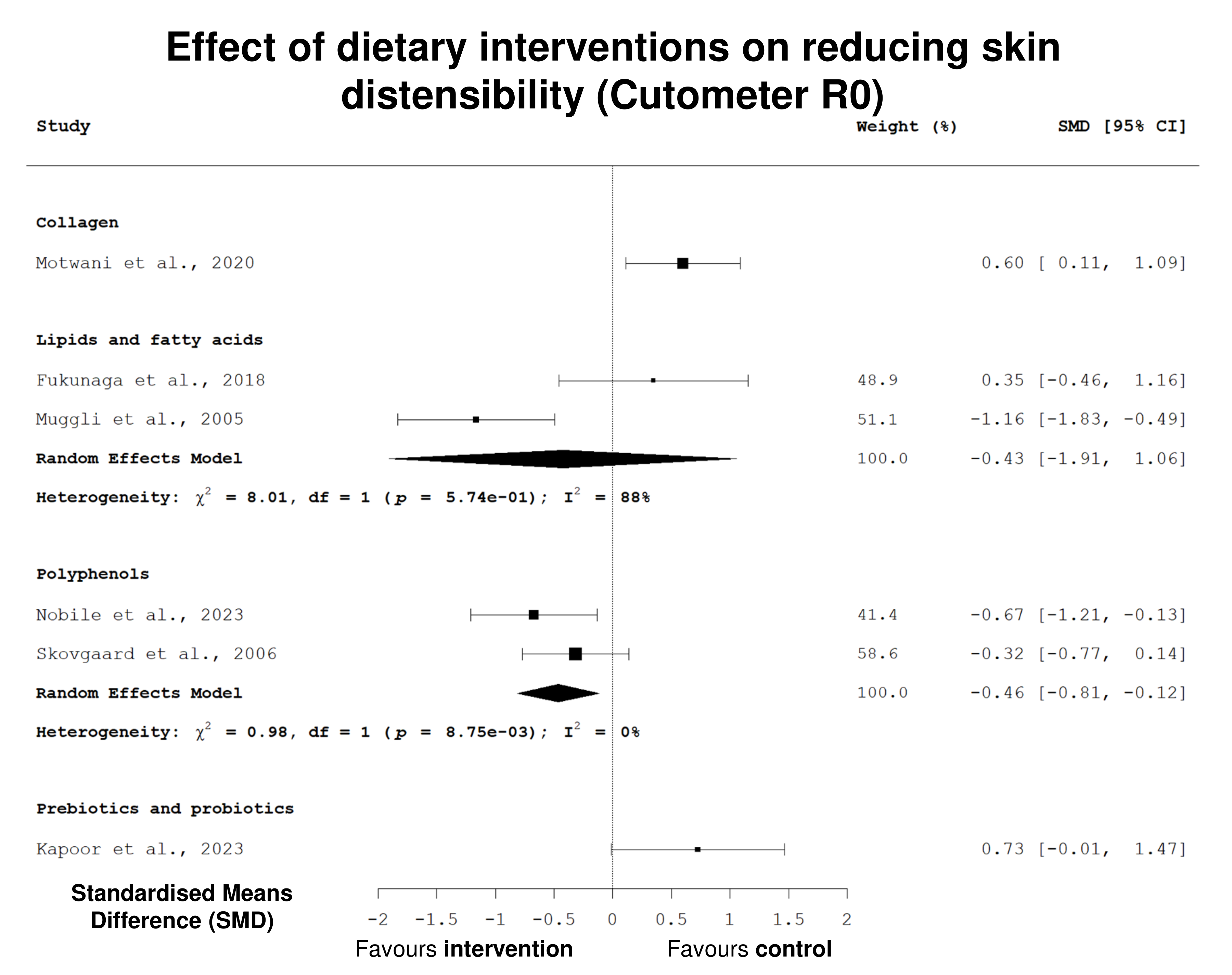

Supplement: Supplementary file 24 — Additional file 24: Forest plot summarising the effect sizes, quantified as Standardised Means Difference (SMD), for studies assessing the impact of dietary interventions on skin distensibility (i.e., R-parameter R0 on the Cutometer) compared to non-interventional controls. Each circle represents a study's effect size. The size of each circle is proportional to its weight in the meta-analysis. Horizontal lines denote 95% confidence intervals. SMD for each study was calculated using Cohen’s d for paired samples (i.e., before vs after dietary intervention). The vertical dotted line indicates the line of no effect (SMD=0). A negative SMD indicates that the dietary intervention favours a less saggy skin (i.e., less skin distensibility, R-parameter R0 on the Cutometer) when compared to non-interventional controls. The pooled effect estimate and pooled 95% Confidence Interval (CI) are computed based on a Random Effects Model and shown as a diamond, in which the diamond’s width represents the range of the 95% CI. The I2 statistic quantifies the proportion of total variation in results across studies investigating the same dietary intervention that is due to heterogeneity rather than chance. An I2 value of 0% indicates no observed heterogeneity; the group of studies examining this dietary intervention are relatively homogeneous. Larger I2 values indicate greater heterogeneity. SMD: standardised means difference. CI: confidence interval. χ2: chi-square. df: degrees of freedom. p: chi-square test p-value. I2: heterogeneity statistic. [file 40101_2025_408_MOESM24_ESM.png]

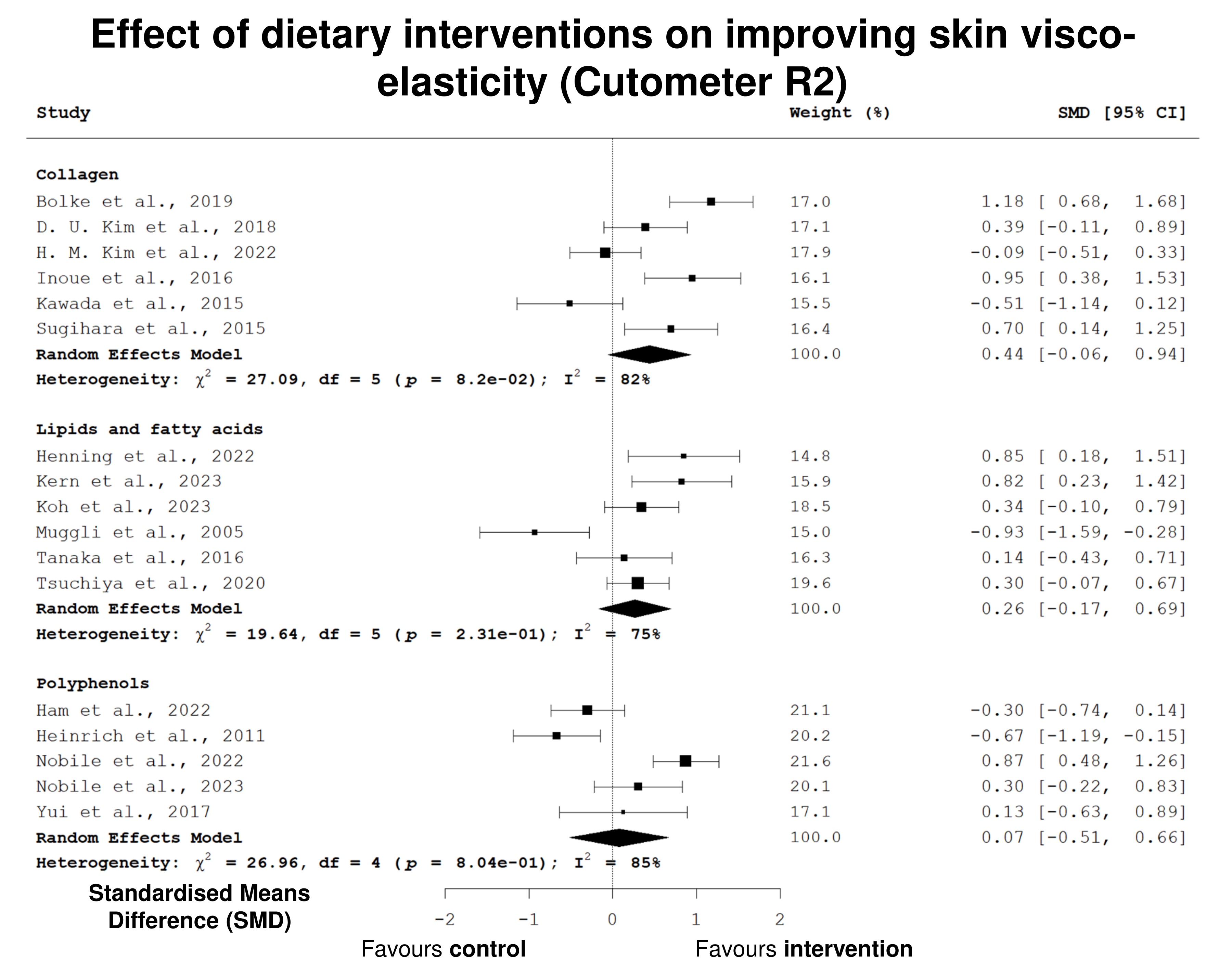

Supplement: Supplementary file 25 — Additional file 25: Forest plot summarising the effect sizes, quantified as Standardised Means Difference (SMD), for studies assessing the impact of dietary interventions on the overall visco-elasticity of the skin (i.e., R-parameter R2 on the Cutometer) compared to non-interventional controls. Each circle represents a study's effect size. The size of each circle is proportional to its weight in the meta-analysis. Horizontal lines denote 95% confidence intervals. SMD for each study was calculated using Cohen’s d for paired samples (i.e., before vs after dietary intervention). The vertical dotted line indicates the line of no effect (SMD=0). A positive SMD indicates that the dietary intervention favours a greater overall visco-elasticity of the skin (i.e., R-parameter R2 on the Cutometer) when compared to non-interventional controls. The pooled effect estimate and pooled 95% Confidence Interval (CI) are computed based on a Random Effects Model and shown as a diamond, in which the diamond’s width represents the range of the 95% CI. The I2 statistic quantifies the proportion of total variation in results across studies investigating the same dietary intervention that is due to heterogeneity rather than chance. An I2 value of 0% indicates no observed heterogeneity; the group of studies examining this dietary intervention are relatively homogeneous. Larger I2 values indicate greater heterogeneity. SMD: standardised means difference. CI: confidence interval. χ2: chi-square. df: degrees of freedom. p: chi-square test p-value. I2: heterogeneity statistic. [file 40101_2025_408_MOESM25_ESM.png]

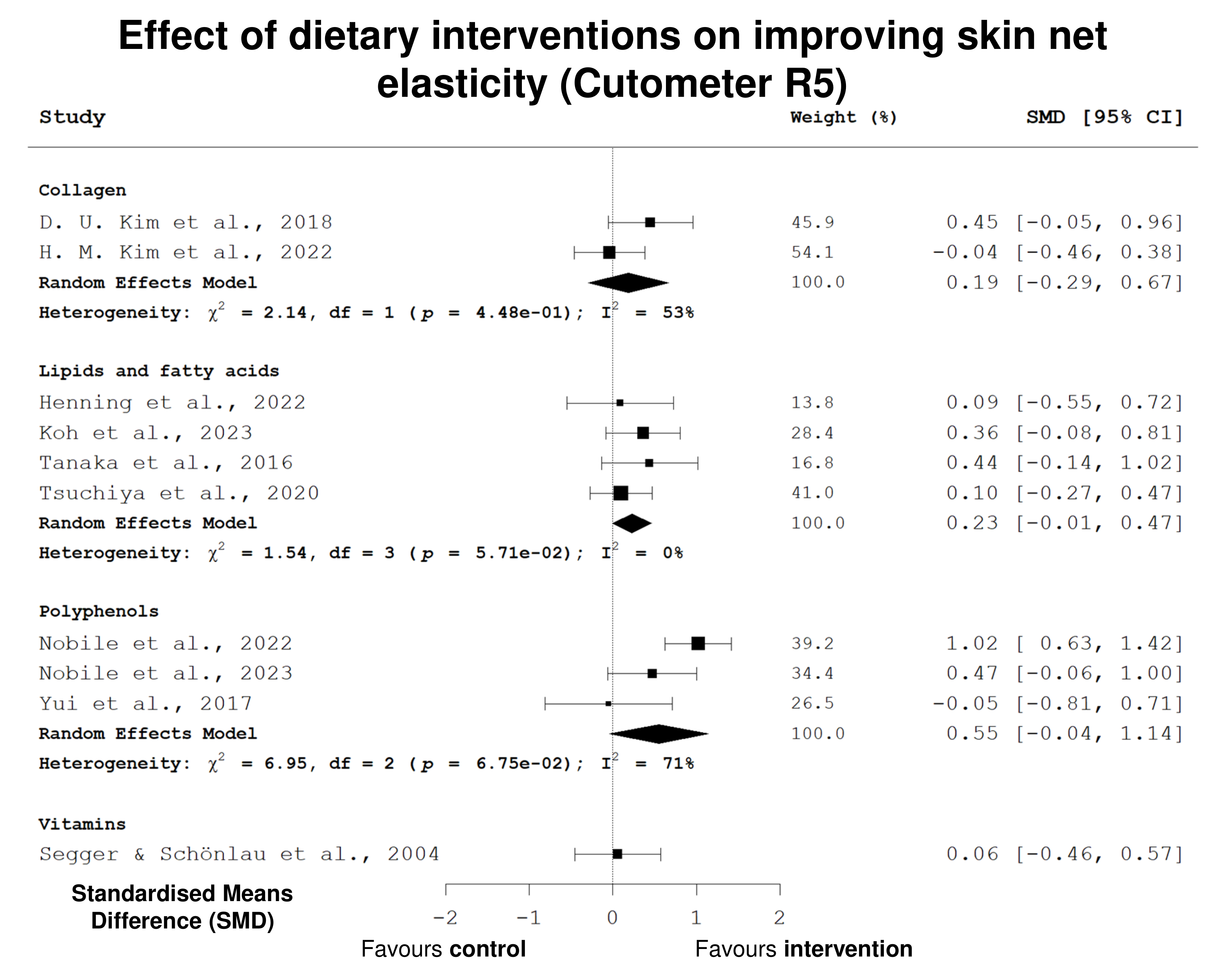

Supplement: Supplementary file 26 — Additional file 26: Forest plot summarising the effect sizes, quantified as Standardised Means Difference (SMD), for studies assessing the impact of dietary interventions on the net elasticity of the skin (i.e., R-parameter R5 on the Cutometer) compared to non-interventional controls. Each circle represents a study's effect size. The size of each circle is proportional to its weight in the meta-analysis. Horizontal lines denote 95% confidence intervals. SMD for each study was calculated using Cohen’s d for paired samples (i.e., before vs after dietary intervention). The vertical dotted line indicates the line of no effect (SMD=0). A positive SMD indicates that the dietary intervention favours a greater net elasticity of the skin (i.e., R-parameter R5 on the Cutometer) when compared to non-interventional controls. The pooled effect estimate and pooled 95% Confidence Interval (CI) are computed based on a Random Effects Model and shown as a diamond, in which the diamond’s width represents the range of the 95% CI. The I2 statistic quantifies the proportion of total variation in results across studies investigating the same dietary intervention that is due to heterogeneity rather than chance. An I2 value of 0% indicates no observed heterogeneity; the group of studies examining this dietary intervention are relatively homogeneous. Larger I2 values indicate greater heterogeneity. SMD: standardised means difference. CI: confidence interval. χ2: chi-square. df: degrees of freedom. p: chi-square test p-value. I2: heterogeneity statistic. [file 40101_2025_408_MOESM26_ESM.png]

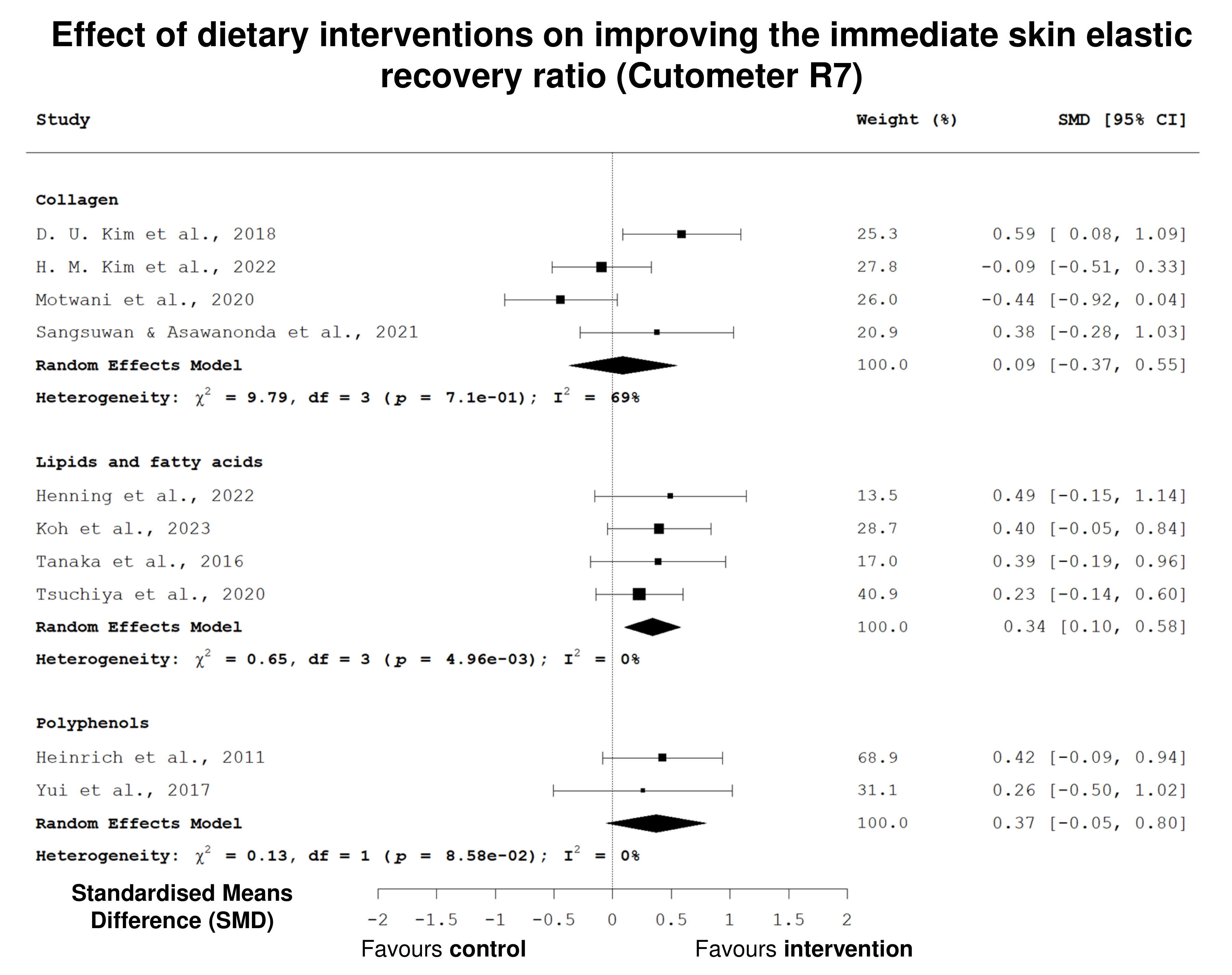

Supplement: Supplementary file 27 — Additional file 27: Forest plot summarising the effect sizes, quantified as Standardised Means Difference (SMD), for studies assessing the impact of dietary interventions on the immediate recovery of the skin after suction (i.e., R-parameter R7 on the Cutometer) compared to non-interventional controls. Each circle represents a study's effect size. The size of each circle is proportional to its weight in the meta-analysis. Horizontal lines denote 95% confidence intervals. SMD for each study was calculated using Cohen’s d for paired samples (i.e., before vs after dietary intervention). The vertical dotted line indicates the line of no effect (SMD=0). A positive SMD indicates that the dietary intervention favours a greater immediate recovery of the skin after suction (i.e., R-parameter R7 on the Cutometer) when compared to non-interventional controls. The pooled effect estimate and pooled 95% Confidence Interval (CI) are computed based on a Random Effects Model and shown as a diamond, in which the diamond’s width represents the range of the 95% CI. The I2 statistic quantifies the proportion of total variation in results across studies investigating the same dietary intervention that is due to heterogeneity rather than chance. An I2 value of 0% indicates no observed heterogeneity; the group of studies examining this dietary intervention are relatively homogeneous. Larger I2 values indicate greater heterogeneity. SMD: standardised means difference. CI: confidence interval. χ2: chi-square. df: degrees of freedom. p: chi-square test p-value. I2: heterogeneity statistic. [file 40101_2025_408_MOESM27_ESM.png]

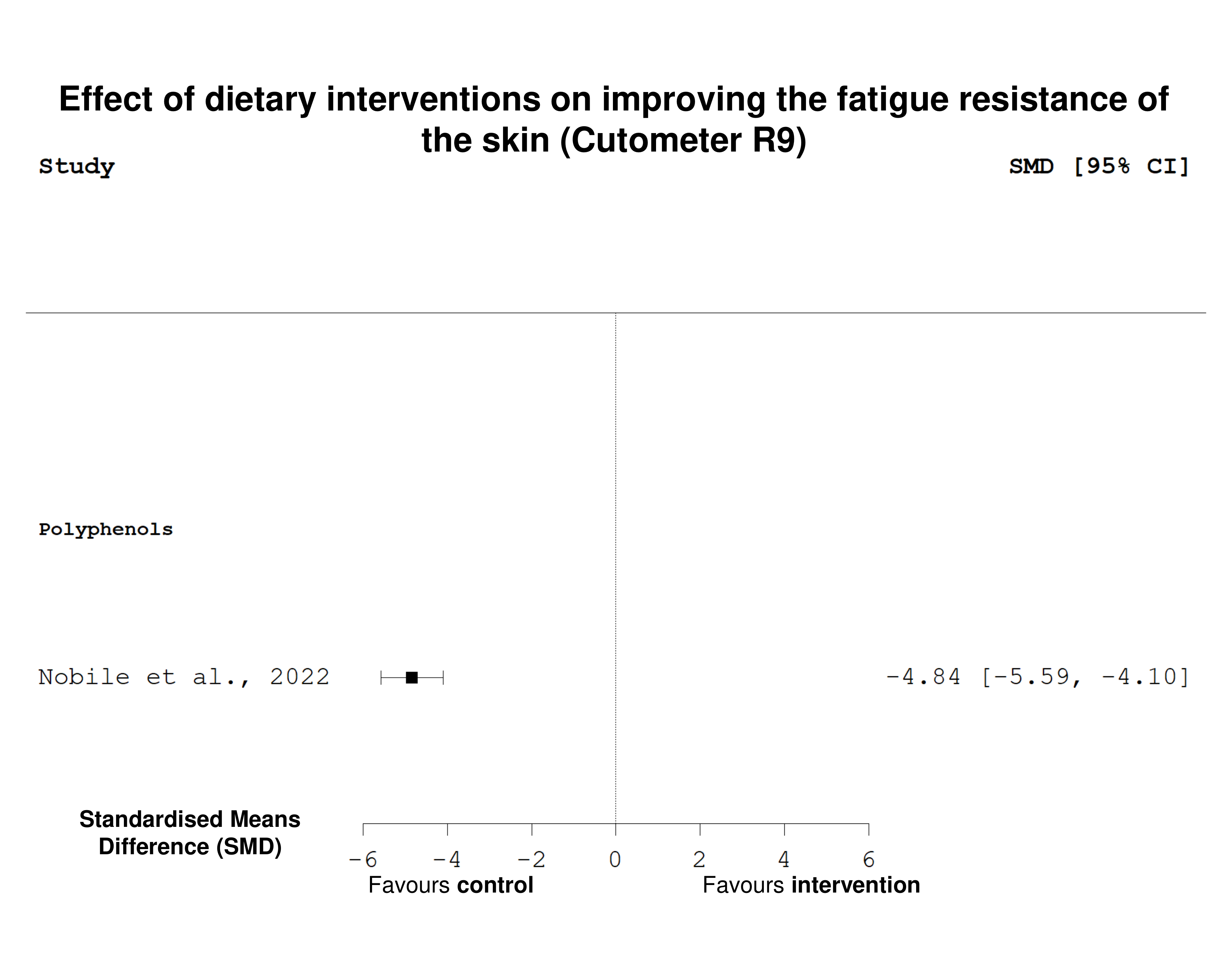

Supplement: Supplementary file 28 — Additional file 28: Forest plot summarising the effect sizes, quantified as Standardised Means Difference (SMD), for studies assessing the impact of dietary interventions on the fatigue resistance of the skin (i.e., R-parameter R9 on the Cutometer) compared to non-interventional controls. Each circle represents a study's effect size. The size of each circle is proportional to its weight in the meta-analysis. Horizontal lines denote 95% confidence intervals. SMD for each study was calculated using Cohen’s d for paired samples (i.e., before vs after dietary intervention). The vertical dotted line indicates the line of no effect (SMD=0). A positive SMD indicates that the dietary intervention favours a greater fatigue resistance (i.e., R-parameter R9 on the Cutometer) when compared to non-interventional controls. The pooled effect estimate and pooled 95% Confidence Interval (CI) are computed based on a Random Effects Model and shown as a diamond, in which the diamond’s width represents the range of the 95% CI. The I2 statistic quantifies the proportion of total variation in results across studies investigating the same dietary intervention that is due to heterogeneity rather than chance. An I2 value of 0% indicates no observed heterogeneity; the group of studies examining this dietary intervention are relatively homogeneous. Larger I2 values indicate greater heterogeneity. SMD: standardised means difference. CI: confidence interval. χ2: chi-square. df: degrees of freedom. p: chi-square test p-value. I2: heterogeneity statistic. [file 40101_2025_408_MOESM28_ESM.png]
